# Variation in Structure and Process of Care in Traumatic Brain Injury: Provider Profiles of European Neurotrauma Centers Participating in the CENTER-TBI Study

**DOI:** 10.1371/journal.pone.0161367

**Published:** 2016-08-29

**Authors:** Maryse C. Cnossen, Suzanne Polinder, Hester F. Lingsma, Andrew I. R. Maas, David Menon, Ewout W. Steyerberg, CENTER-TBI Investigators and Participants

**Affiliations:** 1 Center for Medical Decision Sciences, Department of Public Health, Erasmus Medical Center, Rotterdam, the Netherlands; 2 Department of Neurosurgery, Antwerp University Hospital and University of Antwerp, Edegem, Belgium; 3 Division of Anaesthesia, University of Cambridge/Addenbrooke’s Hospital, Cambridge, United Kingdom; Azienda Ospedaliero Universitaria Careggi, ITALY

## Abstract

**Introduction:**

The strength of evidence underpinning care and treatment recommendations in traumatic brain injury (TBI) is low. Comparative effectiveness research (CER) has been proposed as a framework to provide evidence for optimal care for TBI patients. The first step in CER is to map the existing variation. The aim of current study is to quantify variation in general structural and process characteristics among centers participating in the Collaborative European NeuroTrauma Effectiveness Research in Traumatic Brain Injury (CENTER-TBI) study.

**Methods:**

We designed a set of 11 provider profiling questionnaires with 321 questions about various aspects of TBI care, chosen based on literature and expert opinion. After pilot testing, questionnaires were disseminated to 71 centers from 20 countries participating in the CENTER-TBI study. Reliability of questionnaires was estimated by calculating a concordance rate among 5% duplicate questions.

**Results:**

All 71 centers completed the questionnaires. Median concordance rate among duplicate questions was 0.85. The majority of centers were academic hospitals (n = 65, 92%), designated as a level I trauma center (n = 48, 68%) and situated in an urban location (n = 70, 99%). The availability of facilities for neuro-trauma care varied across centers; e.g. 40 (57%) had a dedicated neuro-intensive care unit (ICU), 36 (51%) had an in-hospital rehabilitation unit and the organization of the ICU was closed in 64% (n = 45) of the centers. In addition, we found wide variation in processes of care, such as the ICU admission policy and intracranial pressure monitoring policy among centers.

**Conclusion:**

Even among high-volume, specialized neurotrauma centers there is substantial variation in structures and processes of TBI care. This variation provides an opportunity to study effectiveness of specific aspects of TBI care and to identify best practices with CER approaches.

## Introduction

Traumatic Brain Injury (TBI) is an important threat to public health with a crude incidence rate of up to 849 per 100,000 people in European countries [1, 2]. TBI is emerging as one of the leading causes of death and disability worldwide resulting in huge personal suffering and far-reaching socioeconomic consequences [3, 4].

Different perspectives on various aspects of care exist, and the evidence underpinning guideline recommendations for treatment of patients with TBI is weak [3, 5]. There is growing realization that randomized clinical trials alone will not be able to provide the evidence base that is needed to address these knowledge gaps [6]. Comparative effectiveness research (CER) has been proposed as a good complementary approach to strengthen the evidence base. CER has been defined as “the generation and synthesis of evidence that compares the benefits and harms of alternative methods to prevent, diagnose, treat, and monitor a clinical condition or to improve the delivery of care” [7]. CER exploits between-center differences in patient management by comparing centers that perform a certain intervention routinely to others that do not. This approach is expected to be particularly suitable for TBI since large between-center differences in both patient management and outcomes have been previously reported [8, 9].

The Collaborative European NeuroTrauma Effectiveness Research in Traumatic Brain Injury (CENTER-TBI) study is a large-scale observational multicenter study focusing on characterization and CER in TBI. The first step for CER is to provide an overview of variation in structures and processes of care in the participating centers (‘provider profiling’). Such an overview can be used to identify areas where large between-center variation exists, to guide future CER analyses. But it can also directly be used for CER. For example, treatment effectiveness of a certain intervention can be studied by comparing outcome in patients from centers that routinely perform the intervention to outcome in patients from centers that do not routinely perform the intervention. Therefore, the objective of the current study is to quantify variation in general structure and process characteristics among centers participating in the CENTER-TBI study and to identify topics for CER.

## Material and Methods

### CENTER-TBI study

CENTER-TBI is a prospective longitudinal observational study conducted in 72 centers from 20 countries across Europe and Israel [3]. One of the global aims is to “identify the most effective clinical care and provide high-quality evidence in support of treatment recommendations and guidelines” [3]. This will be pursued by CER approaches. For more information, see also www.center-tbi.eu. Before the patient inclusion started, a detailed inventory of center characteristics was performed by distributing a set of questionnaires on structures and process of TBI care: The Provider Profiling (PP) questionnaires (S1 File). This set of questionnaires was distributed among 71 centers, since two CENTER-TBI centers represented different departments from the same hospital with similar structures and processes.

### Development process of the Provider Profiling Questionnaires

The PP questionnaires went through a comprehensive developing process to warrant completeness and relevance of topics and face validity of questions. The neurotrauma evidencemap (http://neurotrauma.evidencemap.org/) was searched for gaps and inconsistencies in knowledge of optimal treatment and organization of TBI care, and used to define topics of interest. We included topics relevant for CER as well as topics relevant for descriptive analyses. Initial questions were formulated based on literature and suggestions from experts in the field. Available surveys and questionnaires in the field of TBI or critical care [10, 11] were searched for and used for the (re)formulation of (additional) questions.

Questions related either to structures or processes of general or TBI-specific care. Structure refers to the conditions under which patient care is provided (e.g. the number of beds, trauma center designation, hospital facilities), and process refers to activities that constitute patient care (e.g. general hospital or department policies) [12]. Structural information could be extracted from hospital databases, annual reports and local registries. Process information refers to general policies rather than individual treatment preferences of responsible physicians. General policy was defined as ‘the way the large majority of patients (>75%) with a certain indication would be treated’, recognizing that there might be exceptions. We included open questions and multiple-choice questions. All questions were presented with text boxes that contained definitions and a short explanation about the interpretation and completion of the question. The definitions used in this paper are summarized in the Supplemental material (S2 File).

Experts in the field provided feedback on the initial formulated questions and proposed new questions and topics in three subsequent phases. Consulted experts included neurosurgeons, (neuro)intensivists, neurologists, emergency department (ED) physicians, rehabilitation physicians, medical ethicists, health care economists and epidemiologists. Some of the consulted experts had previous experience with the design and conduct of surveys in the field of TBI or critical care. In a first phase, a small group of involved experts discussed the questionnaires during an email conversation and a group discussion. In a second phase, an international expert panel, consisting of 25 experts from 9 countries, was consulted per email. These experts provided feedback on one or more of the questionnaires. Decisions on proposed content and formulation were then made during a group discussion with a small group of involved experts. These draft PP questionnaires were then pilot-tested in 16 of the participating CENTER-TBI centers. Each center completed two or three questionnaires, such that each questionnaire was pilot-tested at least three times. All answers were checked for unexpected or missing values and ambiguous questions were subsequently reformulated or deleted. Pilot-testers additionally completed a form in which they were asked to provide feedback, which was incorporated accordingly. All these processes resulted in a final set of eleven questionnaires related to different phases of TBI care (see Table 1). In total, there were 321 questions included in the PP.

**Table 1:** Characteristics of the Provider Profiling questionnaires.

Questionnaire	No. of questions	Topics
1.General	41	Structural characteristics of the hospital, catchment area, volume, facilities, staffing characteristics, payment, equipment, costs
2.Medical ethics	17	Department of medical ethics, IRB approval, informed consent procedures
3. Prehospital trauma care	28	First aid initiatives, dispatch systems, emergency services, hospital reception and initial treatment
4. Emergency department	50	Structural characteristics of the ED, imaging, guidelines, ED overcrowding, treatment, admission policy, discharge policy, withdrawal of life support
5. Admission	22	Structural characteristics of the ward, admission policy, guidelines, observations, treatment policy, step down beds, discharge policy
6. Structural and organizational aspects of the ICU	27	Structural characteristics of the ICU(s), staffing characteristics, admission policy, ICU decision making
7. Treatment at the ICU	70	Protocol use, ICP- and CPP monitoring, sedation, non-surgical treatment of severe TBI patients, seizure prophylaxis, treatment of fever, DVT prophylaxis, mechanical ventilation
8. Ethical aspects of the ICU	20	Withdrawal of life support, age and ICU admission
9. Neurosurgery	21	Volume, staffing characteristics, decision making, protocols, surgical management of mass lesions
10. Rehabilitation	14	In-hospital rehabilitation facilities, referral to post-acute care
11. Country	11	Health care policy, dispatch systems, insurance

***Note*.** The provider profiling questionnaires consist of 11 separate questionnaires. Table shows number of questions and topics for each of the questionnaires.

*Abbreviations*. IRB = institutional review board, ED = emergency department, ICU = intensive care unit, ICP = intracranial pressure, CPP = cerebral perfusion pressure, TBI = traumatic brain injury, DVT = deep venous thrombosis prophylaxis

### Distribution of the questionnaires

During presentations and workshops at two consecutive CENTER-TBI investigators meetings, information on the PP questionnaires was provided. Local investigators, as the senior persons supervising the CENTER-TBI study in the centers, were extensively informed in person and per email about the aim of the study and we emphasized the confidentiality of their responses. Additionally, to achieve unequivocal responses, we instructed them on how to respond to the process questions. We emphasized that we were asking for general policies, rather than individual treatment preferences and stimulated discussions with colleagues to identify the general policy of their department/center. Questionnaires were completed using a web-based system (Quesgen Systems Inc.) An instruction video was made available and any questions from local investigators were answered per email.

The local investigators in each center were responsible for the completion process in their center. Staff members with the appropriate expertise and knowledge needed to complete one or more questions or questionnaires. The local investigators were responsible for monitoring progress and checking face validity of all answers. The first author (MC) reminded local investigators regularly and answered any questions by email.

We aimed to receive completed questionnaires before centers started recruiting patients. As CENTER-TBI had a phased start of the inclusion period, PP questionnaires were completed between December 2014 and April 2016.

### Questionnaire completion and data cleaning

A questionnaire was considered completed by a center if > 90% of the questions had been answered. Data from participating centers were included in the current paper if the center had completed the first PP questionnaire (‘general’), since the first questionnaire provides the general structure information necessary for provider profiles. The first author (MC) screened the completed questionnaires for missing values and contacted local investigators if any missings were present. They were asked to complete the missing data if possible or provide a reason for missingness. Data were further screened for outliers and local investigators were contacted to confirm values that were considered out of range.

### Statistical analyses

To estimate reliability of the questionnaires, we included 17 (5%) duplicate questions, including all question formats. We equally included structure and process questions in the duplicate questions. Concordance rates were estimated by calculating the percentage of overlap between duplicate questions, and presented as mean, median and range. For open questions (e.g. what is the number of intensivist in your center), a maximum difference of 10% was considered concordant. For all hospital characteristics in this paper, frequencies and percentages were presented for categorical variables and medians and interquartile ranges (IQR) were presented for continuous variables. For a more in-depth understanding of the variation among centers, we checked whether there were differences between relatively high- and middle-income countries versus relatively lower-income countries, and also if there were differences between countries from different geographic locations (North and West Europe versus South and East Europe and Israel). We used the Chi-square test, and if appropriate, Fisher’s exact test to examine whether differences between groups were statistically significant (p < .05). The designation into relatively lower-income countries was based on a 2007 report by the European Commission [13]. Bosnia Herzegovina, Bulgaria, Hungary, Latvia, Lithuania, Romania and Serbia were subsequently classified as relatively lower-income countries. The subdivision into geographic location was based on the classification by the United Nations. Austria, Belgium, Denmark, Finland, France, Germany, Lithuania, the Netherlands, Norway, Sweden and the United Kingdom (UK) were subsequently classified as countries from West and North Europe, while all other countries were classified as countries from South and East Europe and Israel. Analyses were performed using the Statistical Package for Social Sciences (SPSS) version 21.

## Results

### Completion process

**Fig 1:**
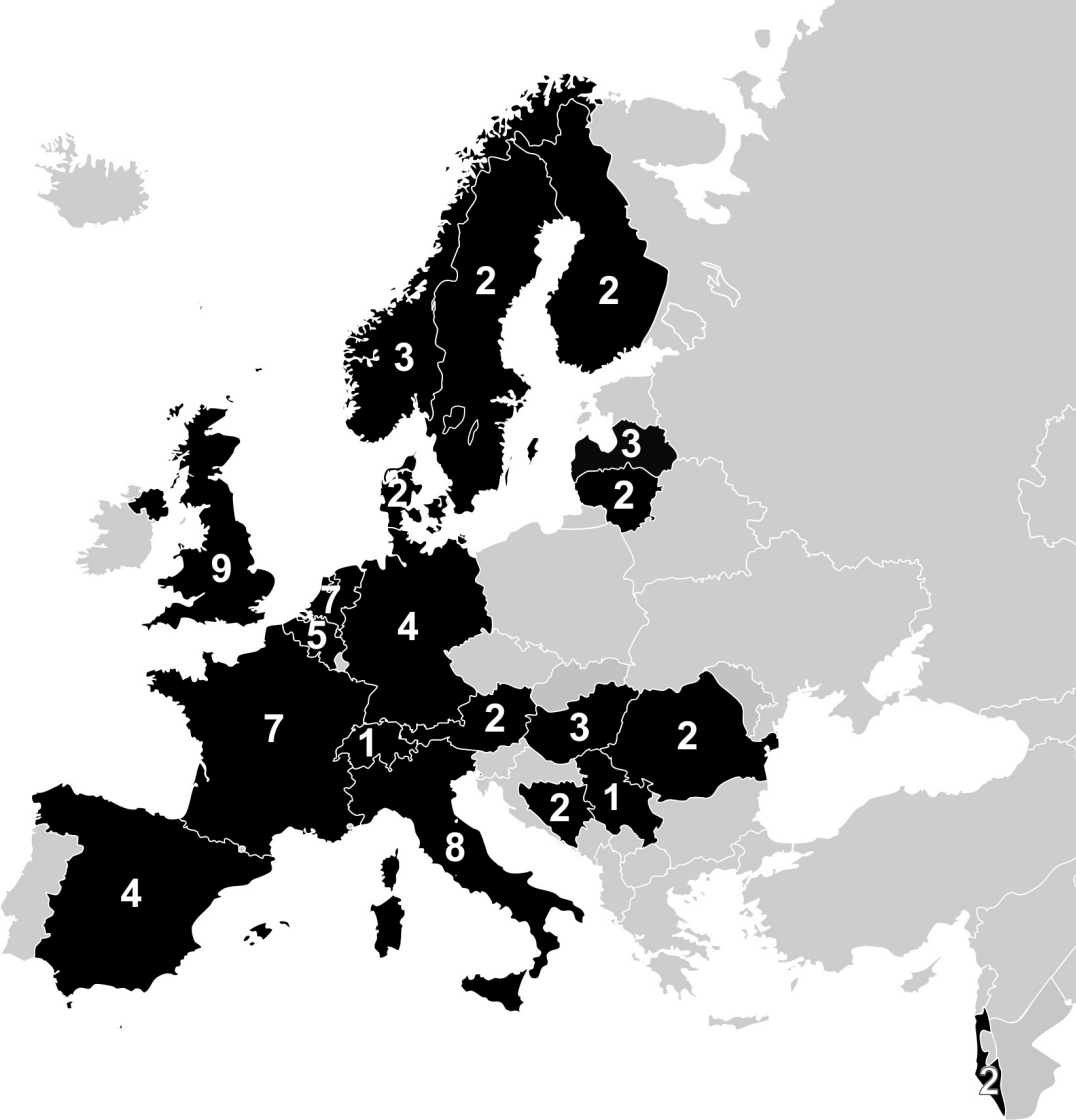
Centers and countries included in the Collaborative European NeuroTrauma Effectiveness Research in Traumatic Brain Injury (CENTER-TBI) study *Note*. Reprinted and updated from Maas et al. (2015). Collaborative European NeuroTrauma Effectiveness Research in Traumatic Brain Injury: a prospective longitudinal observational study. *Neurosurgery*, *76*:*67–80*, under a CC BY licence, with permission from professor A.I. Maas.

All 71 eligible centers completed the provider profiling questionnaire about general structural and process information. Questionnaires were completed by multiple persons per center, including neurologists, neurosurgeons, trauma surgeons, intensivists, research nurses and administrative staff members. The 71 centers were from 20 European countries (see Fig 1). Each country had 1 to 9 participating centers (median = 2.5). The United Kingdom (UK) had most centers participating (n = 9), while Serbia and Switzerland both had one participating center. Thirteen of the included centers were from relatively lower-income countries and 25 centers were from countries in South and East Europe (including Israel).

### Reliability of the questionnaires

The median concordance rate between duplicate questions was 0.85 (mean: 0.81; range 0.44–0.97), meaning that 85% of the responses were similar. Concordance rates were lowest for questions about treatment policy (e.g. on what indications would you admit a patient with mild TBI to the ward) and for open questions (e.g. what is the number of intensivists working at your center). Most multiple-choice questions about structure had concordance rates above 0.90.

### General structural characteristics

The participating centers were predominately academic centers (n = 65, 92%), designated as a level I or II trauma center (n = 54, n = 74%) and situated in an urban location (n = 70, 99%, see Table 2). The majority of participants indicated that they had access to a helicopter platform (n = 57, 80%) and an acute trauma team (n = 63, 89%). Around half of the centers (n = 40, 57%) had a dedicated neuro ICU. Centers from relatively high- and middle-income countries more often indicated that they have a dedicated neuro ICU (n = 35, 61%) than centers from relatively lower-income countries (n = 5, 39%, p = .13, S1 Table). The large majority of centers had participated previously in research about acute cerebral disorders. Fifty-one (72%) centers were involved in more than five neurotrauma research applications over the past five years (see Table 2).

**Table 2:** General structural characteristics of the participating centers (n = 71).

Characteristic	N completed	N (%)*
Academic hospital (vs. non-Academic)	71	65 (92%)
Trauma center designation	71	
- Level I		48 (68%)
- Level II		4 (6%)
- Level III		1 (1%)
- No designation / NA		18 (25%)
Urban location (vs. suburban and rural location)	71	70 (99%)
Helicopter platform	71	57 (80%)
Acute trauma team	71	63 (89%)
The availability of a dedicated neuro ICU	70	40 (57%)
Number of ICUs (median, IQR)	69	3 (2–5)
The availability of an in-hospital rehabilitation unit	70	36 (51%)
Neurotrauma research applications in the past 5 y	71	
- > 5		51 (72%)
- 3–5		13 (18%)
- 1–2		4 (6%)
- 0 or unknown		3 (4%)
Distance nearest trauma center that receives patients with severe TBI (km, median, IQR)	52	56 (17–100)

***Note*.** ICU = Intensive care unit; IQR = Interquartile Range

* Table presents number and percentage of centers unless otherwise specified

The median number of beds in the participating centers was 1000 (IQR 682–1395) of which 31 (IQR 22–44) were ICU beds (see Table 3 and S1 Fig). Centers had a median of 3 (IQR 2–6) resuscitation rooms at the ED and 24 (IQR 16–39) operating rooms. Three (IQR 2–4) of these were potentially available for TBI patients. The median number of annual ED visits was 53,428 (IQR 30,002–90,268). The median number of annual ICU admission was 1240 (IRQ 560–2019), of which 91 (IQR 52–160) were TBI patients.

**Table 3:** Volume characteristics of the participating centers (n = 71).

Characteristic	N completed	Median (IQR)
Number of beds		
Number of ED observational beds	69	16 (7–32)
Number of hospital beds	69	1000 (682–1395)
Number of ICU beds	71	31 (22–44)
Number of resuscitation and operating rooms		
Number of resuscitating rooms	69	3 (2–6)
Number of operating rooms	70	24 (16–39)
Number of operating rooms potentially available for TBI patients^A^	69	3 (2–4)
Number of patients		
Annual ED visits	63	53,428 (30,002–90,268)
Annual ICU admissions	65	1240 (560–2019)
Number of TBI patients		
Annual number of TBI patients at the ICU	63	91 (52–160)
Annual neurosurgical procedures to evacuate contusion	59	9 (4–21)
Annual decompressive craniectomies	56	13 (8–22)

***Note*.** IQR = interquartile range; ED = emergency department; ICU = intensive care unit; TBI = traumatic brain injury; SAH = subarachnoid hemorrhage

^A^ Operating rooms potentially available for TBI patients are the operating rooms that can be used for emergency and non-emergency TBI patients (e.g. trauma operating rooms, neurosurgical operating rooms etc). Rooms that are used for non-TBI surgery in TBI patients (e.g. orthopedic surgery in patients with multiple trauma) should be excluded here.

Seventy-five per cent (n = 53) of the centers had separate 24/7 emergency operation rooms. The majority of centers indicated that they had an electronic patient system at the ward (n = 57, 80%) and the ICU (n = 56, 79%). There was variation in the organization of the ICU in the participating centers; i.e. 45 (64%) centers had a closed ICU organization, 3 (4%) an open ICU organization and the remainder (n = 22, 32%) a mixed ICU organization. Centers from relatively high- and middle-income countries more often reported that they had a closed ICU structure (n = 40, 70%) compared to centers from relatively lower-income countries (n = 5, 39%). Step down beds were available in 71% (n = 50) of the centers. Centers from North and West Europe more often reported that they had a step down bed facility than centers from South and East Europe and Israel (n = 36, 80% vs. n = 14, 56%, p = .03, S1 Table). Maximum laboratorium turnaround times, the possibility for in-hospital coma stimulation and the location of TBI relevant facilities also varied widely among the included centers (see Table 4).

**Table 4:** Hospital facilities of the participating centers (n = 71).

Characteristic	N completed	N (%)
General		
Separate 24/7 emergency operation rooms	71	53 (75%)
Electronic patient system		
- Ward	71	57 (80%)
- ICU	71	56 (79%)
Facility for overnight observation	69	54 (78%)
Lab turnaround time ^A^	68	
- 0-30minutes		25 (36%)
- >30 minutes		26 (38%)
- NA. No lab SOP at the ED		17 (25%)
Organization of the ICU	70	
- Closed		45 (64%)
- Open		3 (4%)
- Mixed		22 (32%)
Step down beds	70	50 (71%)
In-hospital coma stimulation	70	34 (49%)
TBI related		
Location TBI facilities	71	
- Different buildings		20 (28%)
- Same building, different floors		45 (63%)
- Same building, same floors		6 (9%)

***Note*.** ICU = intensive care unit; NA = not applicable; SOP = Standard Operating Procedures; TBI = traumatic brain injury

^**A**^ The laboratory turnaround times that are record in the lab Standard Operating Procedures (SOP) at the emergency department for severely injured patients

On average 14 neurologists, 10 neurosurgeons, 17 intensivists, 4 trauma surgeons and 10 ED physicians were working in the centers (see Table 5). Nearly all centers (n = 69, 97%) had at least one residency program for trainees towards becoming a specialist. The specialist most often in charge of TBI patients at respectively the ED, ward and ICU were predominately ED physicians, neurosurgeons and intensivists. Most centers had 24/7 in-house availability of OR personnel (n = 62, 87%) and CT technicians (n = 66, 93%). Median intensivist-to-patient ratio, and ICU nurse-to-patient ratio were 1: 5 (IQR 1:3 to 1:8) and 1:2 (IQR 1:1 to 1:3). Night coverage at the ICU was performed by a certified intensivist in two-third of the centers (n = 44, 65%) and by a trainee or fellow in the remainder of centers. Almost all centers from the relatively lower-income countries (n = 12, 92%) reported that night coverage was performed by a certified intensivist, in comparison to 58% of the centers from the relatively high- and middle-income countries. Also, more centers from South and East Europe (n = 22, 88%) had night coverage by a certified intensivist, compared to centers from North and West Europe (n = 22, 51%, S1 Table).

**Table 5:** Staffing characteristics of the participating centers (n = 71).

Characteristic	N completed	N (%)*
Number of specialists (median, IQR) ^A^		
- Neurologist	71	14 (8–21)
- Neurosurgeon	68	10 (7–13)
- Intensivist	68	17 (10–28)
- Trauma surgeon	68	4 (0–10)
- ED physician	69	10 (3–19)
Residency programs		
- Neurologist	70	65 (93%)
- Neurosurgeon	71	67 (94%)
- Intensivist	71	64 (90%)
- Trauma surgeon	71	36 (51%)
Availability OR personnel	71	
- 24/7 in-house availability		62 (87%)
- On call within 30 minutes		9 (13%)
Availability CT technicians	71	
- 24/7 in-house availability		66 (93%)
- On call within 30 minutes		5 (7%)
Intensivist-to-patient ratio (median, IQR)	69	1: 5 (1: 3–1: 8)
ICU nurse-to-patient ratio (median, IQR)	69	1: 2 (1: 1–1: 3)
Night coverage ICU	68	
- Certified intensivist/ ICU physician		44 (65%)
- Trainee (in residency training)		20 (29%)
- Fellow in training for ICU		4 (6%)

***Note*.** IQR = interquartile range; ED = emergency department; OR = operating rooms; CT = computed tomography

* Table presents number and percentage of centers unless otherwise specified

^**A**^ Number of specialists is displayed per 40-hour workweek.

### General process characteristics

With regard to computed tomography (CT) scanning in patients with mild TBI at the ED, 79% of the centers (n = 54) indicated to use CT guidelines (see Table 6). In addition, seven centers (10%) from Austria, Denmark, France, Spain and Sweden routinely determine S100B as a prognostic biomarker for neurological deterioration at the ED. There was variation among centers in their ICU admission policy; i.e. 44 (64%) centers generally admit patients with moderate TBI (Glasgow Coma Scale (GCS) 9–12) and CT abnormalities to the ICU, while 25 (36%) centers only admit these patients to the ICU in the presence of other risk factors. This variation was also shown for moderate TBI patients without CT abnormalities and patients with mild TBI on anti-coagulant therapy. There was a trend towards a higher ICU admission rate in centers from relatively high- and middle-income countries than in centers from relatively lower-income countries (S2 Table).

**Table 6:** General process information of the participating centers (n = 71).

Characteristic	N Completed	N (%)
Emergency department		
Use of CT scan guidelines at the ED	68	54 (79%)
Routine use of S100B as prognostic biomarker at the ED	71	7 (10%)
ICU admission policy		
Patients with moderate TBI (GCS 9–12) without CT abnormalities are admitted to the ICU	69	
- No or only in the presence of other risk factors		50 (72%)
- General policy		19 (28%)
Patients with moderate TBI (GCS 9–12) with CT abnormalities are admitted to the ICU	69	
- No or only in the presence of other risk factors		25 (36%)
- General policy		44 (64%)
Patients with mild TBI (GCS 13–15) using anti-coagulant therapy are admitted to the ICU	69	
- No or only in the presence of other risk factors		53 (77%)
- General policy		16 (23%)
ICP monitoring		
ICP monitoring is performed in patients with GCS<9 and CT abnormalities	67	
- No or only in the presence of other risk factors		6 (9%)
- General policy		61 (91%)
ICP monitoring is performed in patients with GCS<9 without CT abnormalities	67	
- No or only in the presence of other risk factors		52 (78%)
- General policy		15 (22%)
ICP monitoring is performed in patients with intraventricular hemorrhages	67	
- No or only in the presence of other risk factors		46 (69%)
- General policy		21 (31%)
ICP sensors that are used at the ICU:	67	
- Parenchymal		21 (31%)
- Ventricular		6 (9%)
- Both		40 (60%)
Management of elevated ICP		
Threshold for medical management of elevated ICP	66	
- >15mmHg		3 (5%)
- >20mmHg		57 (86%)
- >25mmHg		6 (9%)
Threshold for decompressive craniotomy in elevated ICP	61	
- >20mmHg		7 (12%)
- >25mmHg		35 (57%)
- >30mmHg		19 (31%)
ICU policies		
Structural variation between (neuro)surgeons with regard to their decision to place an ICP sensor	69	33 (48%)
General policy with regard to the management of extremity fractures in patients with sTBI	68	
- Damage control		58 (85%)
- Definitive care		10 (15%)

***Note*.** CT = computed tomography; ED = emergency department; ICU = intensive care unit; ICP = intracranial pressure; BTF = Brain Trauma Foundation; GCS = Glasgow Coma Scale; sTBI = severe traumatic brain injury

The large majority of participants (n = 61, 91%) indicated that their general policy is to insert intracranial pressure (ICP) monitors in patients with GCS <9 and CT abnormalities. However, centers vary in whether they would place an ICP monitor in patients with GCS <9 without CT abnormalities and patients with intraventricular haemorrhages. Variation in ICP monitoring is also reported within the centers, since half of the centers indicated that there is structural variation between (neuro)surgeons in their center with regard to the decision to place an ICP monitor. The threshold for medical management of elevated ICP was 20 mmHg in the large majority of centers (n = 57, 87%). However, centers varied widely in their threshold for decompressive craniotomy; i.e. in 12% (n = 7) the threshold was 20 mmHg, in 57% (n = 35) the threshold was 25 mmHg and in 31% (n = 19) the threshold was 30 mmHg.

### Insurance and payment systems

In the majority of countries (n = 16, 80%), a health care insurance was compulsory for all inhabitants. In 45% of the countries (n = 9), patients nevertheless had to pay a part of the delivered care themselves via either a co-payment (5 countries) or a deductible (4 countries). Most centers were funded by the government (n = 60; 85%). Centers typically got reimbursed by all-in amounts per patient rather than by payment for individual interventions. Most doctors received a fixed monthly salary (n = 58, 82%). In 11% (n = 8) of the centers, doctors received an additional fee for services. Twenty-three (32%) centers received additional payment for the treatment of privately insured patients.

## Discussion

We found considerable variation in general structure and process characteristics among 71 specialized neurotrauma centers participating in the CENTER-TBI study. Most of these centers were high-volume academic level I trauma centers situated in an urban location. Centers varied widely in their ICU organization, hospital facilities and admission- and treatment policies. The effectiveness of these structures and interventions can therefore adequately be studied with CER.

Our provider profiling questionnaires have strengths and limitations. One of the strengths is the comprehensive development process, which consisted of several stages and involved many experts. As a consequence, the questionnaires address all aspects relevant to TBI care. Secondly, local investigators were extensively informed about the aim, procedures and practical issues during presentations, workshops and emails. This might explain the 100% response rate. The length of our questionnaires can be regarded as a limitation. Long questionnaires have been associated with lower data quality [14, 15], an effect that is often due to fatigue and boredom [15]. Since the questionnaires could be spread over time and over different persons, the negative effect of length was however confined.

Another limitation of our study concerns the generalizability of our findings. The included centers comprise a group of neurotrauma centers participating in a European multicenter study. Our findings therefore cannot be generalized to all centers caring for neurotrauma patients in Europe. Furthermore, our study provides information on what centers reported rather than characteristics that were directly observed. Therefore, we cannot exclude that some of our findings provide a too optimistic picture. For example, almost all centers indicated that they would insert an ICP monitor in patients with severe TBI and CT abnormalities, which is recommended by Brain Trauma Foundation guidelines. However, a systematic review about guideline adherence reported that ICP monitoring guidelines were only followed in one-third of the patients [5]. Later, results from the ongoing CENTER-TBI study will provide insight into discrepancies between reported and actual policies in the participating centers.

The concordance rate between duplicate questions (median: 0.85), indicates a certain degree of subjectivity in the responses. The concordance rate was especially low for process questions, which indicates that there might be differences in policy among wards and doctors, no clear policy at all or difficulties in understanding and interpreting the questions. It might also indicate that some of the doctors that completed the questionnaire might not be representative of their department or center. Although our concordance rate was very similar to a 2001 survey study among European countries [11], results on process characteristics should be interpreted with caution. The reported concordance rate does not account for chance concordance since no statistical measures are available that do account for chance and can also provide one figure for different outcomes (dichotomous, categorical and continuous) that we had in our questionnaire. When interpreting the concordance rate, it should however be acknowledged that some answers might be similar by chance.

Finally, there were only 13 centers from a relatively lower-income country and 25 centers from South and East Europe (including Israel). We therefore had limited power to detect differences between centers from relatively high-and middle-income countries versus centers from relatively lower-income countries and centers from different geographic locations.

Although we studied a sample of highly specialized centers, we found substantial differences in important structural and process characteristics. Largest differences were seen in the specialization and organization of the ICU, i.e. half of the centers indicated to have a dedicated neuro ICU and 64% indicated to have a closed ICU organization. Additionally, rehabilitation facilities varied widely, with half of the centers having an in-hospital rehabilitation unit and the possibility for coma stimulation. We also found large differences in the reported policies regarding ICU admission and ICP monitoring across centers. The variation in structure and process among specialized neurotrauma centers was in line with previous survey studies [11, 12]. Enblad and associates [11] included European centers with a particular interest in neuro ICU and brain monitoring in their survey study. They also found large between-center differences in structures of care (e.g. 76% had a separate NICU, 50% had a neurosurgeon as ICU director). Checkley and associates [12] reported similar findings. They conducted a survey in 69 centers participating in the United States critical illness and injury outcome study. The majority of their centers were teaching hospitals with critical care training. However, 58% of their centers had a closed ICU organization and their annual hospital admission rate ranged from 1,170 to 56,330, indicating large between-center differences in volume. Also there were large differences in the protocols available at their surveyed ICUs.

Although in this study we only reported on general structure and process characteristics, it is clear that the between-center variation is substantial and provides an opportunity for CER. Variation among centers and countries comprises an important prerequisite for CER and enables between-center and between-country comparisons of effective structures and processes of care. We can for example study the influence of a dedicated neuro ICU on outcome in severe TBI patients by studying patients’ outcome in the 40 centers with a dedicated neuro ICU and in the 30 centers without a dedicated neuro ICU. This requires outcome data on patient level, which are currently collected in the CENTER-TBI study. In such a comparison it is important to correct for differences in other structural and process characteristics between these centers, which can potentially be accomplished with advanced statistical modelling. Other potential interesting topics for CER based on the current study include the effectiveness of an in-hospital rehabilitation unit, the effectiveness of high-volume vs. low-volume hospitals, the effectiveness of closed vs. mixed ICU organization, and the effectiveness of admission- and ICP monitoring policies.

## Conclusion

Even among high-volume, specialized neurotrauma centers there is substantial variation in structures and processes of TBI care. This variation provides an opportunity to study effectiveness of specific aspects of TBI care and to identify best practices with CER approaches.

## Supporting Information

S1 Fig Distribution of number of beds. (PDF)

S1 File The Provider Profiling Questionnaires. (PDF)

S2 File Definitions. Note. Table presents all definitions used in the paper in the order that they are used in the results section of the paper. TBI = traumatic brain injury; ICU = intensive care unit
(PDF)

S1 Table Structural characteristics that show substantial variation among the participating centers. ^A^ P-value for the difference between high/middle and low income countries ^B^ P-value for the difference between North-West and South-East Europe and Israel
(PDF)

S2 Table Process characteristics that show substantial variation among the participating centers. ^A^ P-value for the difference between high/middle and low income countries ^B^ P-value for the difference between North-West and South-East Europe and Israel
(PDF)
