# Supplementary figures and images for: Variation in Structure and Process of Care in Traumatic Brain Injury: Provider Profiles of European Neurotrauma Centers Participating in the CENTER-TBI Study
Source: PLoS One. 2016 Aug 29;11(8):e0161367. doi: 10.1371/journal.pone.0161367 (PMC5003388; doi:10.1371/journal.pone.0161367)

## 1. Number of ED beds

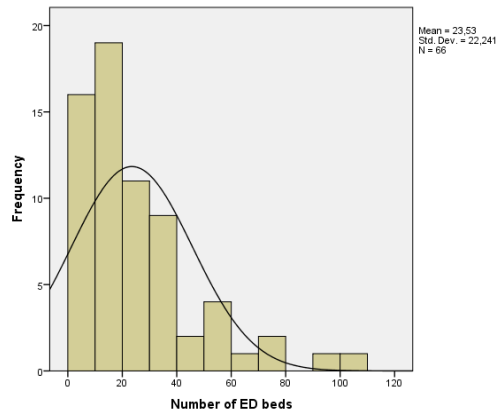

## 2. Number of hospital beds

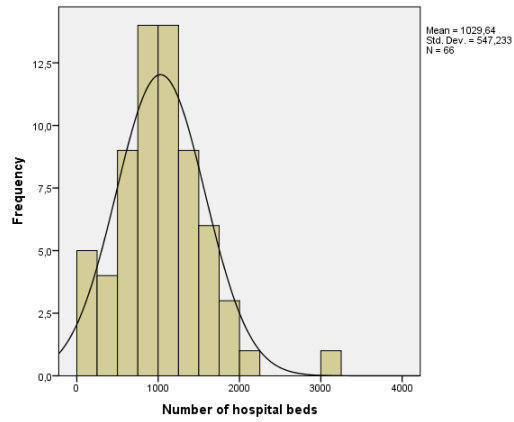

## 3. Number of ICU beds

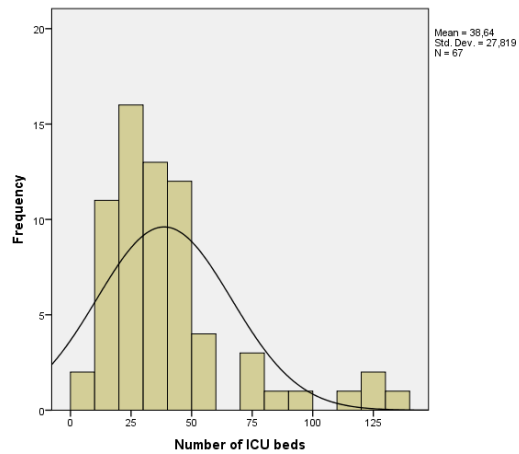

Supplement: S1 Fig — (PDF) [file pone.0161367.s001.pdf]
