# Supplementary material for: Variation in Structure and Process of Care in Traumatic Brain Injury: Provider Profiles of European Neurotrauma Centers Participating in the CENTER-TBI Study
Source: PLoS One. 2016 Aug 29;11(8):e0161367. doi: 10.1371/journal.pone.0161367 (PMC5003388; doi:10.1371/journal.pone.0161367)
Supplement: S1 File — (PDF) [file pone.0161367.s002.pdf]

Provider Profiling Questionnaire

CENTER-TBI

Questionnaire 1: General Questions

This questionnaire can be completed by the CENTER-TBI local investigator

The Local investigator is the senior clinician(s) at your hospital involved in supervision of CENTER TBI

For the completion of this questionnaire, we advise you to ask help from a data manager, administrative staff member and/or someone from the financial department in your hospital, since we ask for hospital data in this questionnaire. It is very important that this information is accurate, and searched for in annual reports, registries and other data sources rather than estimated.

This questionnaire also includes questions about the general policy in your hospital. The responses to these questions should represent, as best as practicable, a general consensus on treatment at your centre, rather than individual management preferences. Consequently, you should provide responses that describe not what you would do personally, but how the majority of patients would generally be treated in your centre.

There are no 'right' or 'wrong' answers so please give us a realistic and honest view of how the care in your hospital is organized. Your answers will only be used to answer the scientific questions in CENTER TBI and no information in any form will be reported on individual centre level. Some of the questions may seem similar, but please answer all questions.

If you have any questions or problem, please contact:
Maryse Cnossen, PhD student (m.c.cnossen@erasmusmc.nl)

Information about the completer of the questionnaire

Other than the CENTER-TBI investigator, which of the following individuals was involved in completion of this questionnaire?
Select all that apply

- ☐ Neurologist
- ☐ Neurosurgeon
- ☐ Trauma Surgeon
- ☐ Emergency Department (ED) physician
- ☐ Administrative staff member / data manager / financial department
- ☐ Other, please specify…………….
- ☐ NA. The questionnaire was completed solely by the CENTER TBI local investigator

Structural characteristics of the hospital

1.What type of hospital is your hospital?

- o Academic / University hospital
- o Nonacademic hospital

Academic / University Hospital = when your hospital is part of or affiliated to a University. An Academic/University hospital aims not only to deliver high-standard patient care, but also contributes to research and education

2. What is the hospital location?

- o Urban
- o Suburban
- o Rural

Urban location = a hospital in or very near to a city; the area is crowded<br>Suburban location = in between urban and rural location.<br>Rural location = a hospital in a location in or very near to the countryside, the area is not crowded

3. How are important wards / facilities in your hospital for Traumatic Brain Injury (TBI) patients (e.g. Emergency Department (ED), Intensive Care Unit (ICU), hospital ward, surgery rooms, scanning) situated?

- o All facilities that are related to TBI patients are in the same building and on same floor
- o Facilities are distributed over different buildings
- o Facilities are located in the same building but on different floors

Some hospitals consist of different buildings. Only tick the different buildings or different floor option if this affects TBI patients. So think of the typical course of a TBI patient (ED, ICU, hospital ward, surgery, CT scan, MRI scan etc.). Are these facilities situated in different buildings or on different floors?

4. Is your hospital officially designated as trauma centre?

- o No
- o Yes
- o N/A in our country/region

In some countries, hospitals designated as trauma centres are categorized, for example level 1 or 2 trauma centre. In other countries, these categories do not apply. If you live in a country that does not explicitly designate trauma centres, please select the N/A box.

4b. If your hospital <u>is</u> a designated Trauma Centre, what is the designation level?

- o Level I
- o Level II
- o Level III

A **Level I trauma centre** is a regional resource centre and generally serves large cities or population-dense areas. A level I trauma centre is expected to manage large numbers of severely injured patients (at least 1,200 trauma patients yearly or have 240 admissions with an Injury Severity Score of more than 14). It is characterized by 24-hour in-house availability of an attending surgeon and the prompt availability of other specialties (e.g. neurosurgeon, trauma surgeon).<br><br>A **Level II trauma centre** provides comprehensive trauma care in either a population-dense area in which a level II trauma centre may supplement the clinical activity and expertise of a level I institution or occur in less population-dense areas. In the latter case, the level II trauma centre serves as the lead trauma facility for a geographic area when a level I institution is not geographically close enough to do so. It is characterized by 24-hour in-house availability of an attending surgeon and the prompt availability of other specialties (e.g. neurosurgeon, trauma surgeon).<br><br>A **level III trauma centre** has the capacity to initially manage the majority of injured patients and have transfer agreements with a level I or II trauma center for seriously injured patients whose needs exceed the facility's resources. A level III trauma centre must have continuous general surgical coverage

4c. If your hospital is a designated Trauma Centre how far away is the next nearest designated trauma centre?

- …………………..km
- ……………………miles

5. What Glasgow Coma Scale (GCS) scores are considered as mild, moderate and severe Traumatic Brain Injury (TBI) in your hospital:

	Lowest GCS value	Highest GCS values
Mild TBI	…………………..	…………………..
Moderate TBI	…………………..	…………………..
Severe TBI	…………………..	…………………..

There are differences between countries and hospitals in how they classify mild, moderate and severe TBI. Please give the lowest and highest GCS values that is considered as mild, moderate and severe. For example: severe TBI might have a lowest value of 3 and a highest value of 8.<br><br>You can use hospital or national guidelines here. If these are not available, we would recommend, for example, an email exchange with colleagues to check that the answer that you provide us here represents the view of most of the persons in your department.

6. What specialty is most often responsible (in charge) for the treatment of Traumatic Brain Injury (TBI) patients in your hospital? Distinguish Emergency Department (ED), Hospital ward (ward where TBI patients are generally admitted) and Intensive Care Unit (ICU) stay?

Please specify per stratum, what specialism is most often in charge. This does not automatically have to be the person with the most contact hours with the patients.<br><br>Only select N/A if it is not possible to select one specialty that is most often in charge.

	ED	Ward	ICU
ED physician	☐	☐	☐
Neurologist	☐	☐	☐
Neurosurgeon	☐	☐	☐
Intensivist	☐	☐	☐
Trauma Surgeon	☐	☐	☐
General or Orthopaedic surgeon	☐	☐	☐
N/A. Care for TBI patients Is always	☐	☐	☐

multidisciplinary			
Other, please specify………………………		☐	☐

Catchment Area

7. How many other hospitals within a 5 and 10 km radius of your hospital receive and treat patients with severe Traumatic Brain Injury (TBI)?
5 km ………… hospitals
10 km ………… hospitals

This question refers to other hospitals that have the capacity to treat severe TBI patients. Exclude hospitals that receive severe TBI, but only stabilize and transfer patients to another hospital

Volume
8. How many beds does your hospital have in total?
……………………………………..

9. How many beds does your hospital have in the following wards and how many of these beds are potentially available for Traumatic Brain Injury (TBI) patients?

Ward	Total number of beds	Number of beds available for TBI patients
Emergency department	……………………………	……………………………
Trauma ward	……………………………	……………………………
Trauma surgery ward	……………………………	……………………………
Neurology ward	……………………………	……………………………
Neurosurgery ward	……………………………	……………………………
Intensive Care Unit	……………………………	……………………………
Surgical ward	……………………………	……………………………
Another ward where TBI patients may be referred to. Please specify……………..	……………………………	……………………………

Please provide the total amount of beds in your hospital per ward in the first column (total number of beds) and then search for the number of beds that are potentially available for TBI patients. This is the total number of beds minus the beds that are already scheduled for something else (for example planned operations).<br><br>If you do not have one of the mentioned wards, then answer 0 under total number of beds and number of beds available for TBI patients.<br><br>It might be possible that in your hospital, some of these wards are combined, for example a combination between the neurology and neurosurgery beds (clinical neuroscience). If that is the case, type 0 as the entry for the neurology and neurosurgery rows, and type: ‘clinical neuroscience’ under ‘another ward’. Please also explain briefly what your entry means: E.g. “clinical neuroscience = beds for neurology and neurosurgery patients”

Surgical facilities
10. How many operation rooms does your hospital have in total?
…………………………………………………….

This question refers to the total number of operation rooms in your hospital, not just the operating rooms that might be related to the care of TBI patients

11. How many operation rooms are potentially available for Traumatic Brain Injury (TBI) patients?

…………………………………………………..

Here, we want to know how many rooms are available for TBI patients. You will first have to find out which operation rooms are used for surgery in TBI patients (trauma operating rooms, neurosurgical operating rooms etc.), and then count these numbers.<br>Exclude operating rooms that are used for non-TBI surgery in TBI patients – e.g. orthopaedic surgery in patients with multiple trauma

12. Does your hospital have separate 24/7 emergency operating rooms?
  - No
  - Yes

The response to this question should address operating rooms that are exclusively used for emergency surgery, and **never** used for planned or elective surgery

13. Are Operating Room (OR) personnel available 24/7?
Tick one option here
  - No.
  - Yes. There is 24/7 in-house availability of OR personnel
  - Yes. OR personnel are on call and would arrive in response to a call out within 30 minutes
  - Yes. OR personnel are on call and would arrive in response to a call out, but not within 30 minutes

The term "OR personnel" in this question refers to Operating Room nurses, anesthesiologists, anesthesiology assistants, etc.

Staffing

14. Do you have a residency program for the following clinical specialties in your hospital? If the answer is 'yes', how many years do these residency programs take, and how many trainees are appointed to the start of the program in <u>your</u> hospital?

Specialism:	Yes / No:	Number of years :	Number of trainees that start annually (maximum):
Trauma surgeon	Yes / No	….. years	……….. trainees
Neurosurgeon	Yes / No	….. years	……….. trainees
Neurologist	Yes / No	….. years	……….. trainees
Intensivist	Yes / No	….. years	……….. trainees

In this response, the term 'trauma surgeon' refers to an individual who specializes in trauma surgery, not a general surgeon or orthopaedic surgeon who happens to perform damage control surgery as part of wider responsibilities.

15. How many hours a week is a Full Time Equivalent (FTE) in your hospital:
For doctors …………. hours
For nurses ……………hours

FTE = Full time equivalent. This will provide the basis for calculation of staffing resources in subsequent questions. In these subsequent questions '1 FTE' may be constituted by one person who works on a fulltime basis, but can also refer to two persons who work half-time.<br><br>We are aware that many physicians undertake a great deal of overtime work (weekend, night shifts) and often work more than is stated in their contract. For this question we are not interested in how many hours they actually work, but in how many hours they work according to their contract.

16. How many neurosurgeons (in FTE) work at your hospital?
……………….FTE neurosurgeons

……………… FTE neurosurgery trainees in residency training
…………….. FTE neurosurgery trainees not in residency training

The amount of FTEs do not have to be a whole number. If the amount of FTE is, for example, 3.3, please write down '3.3' here and not '3'!<br>If there are persons with out of hours work that is contracted and paid for, you can count them as > 1 FTE. For example, if there is a physician that is paid for 60 hours a week and 48 hours a week is considered as a FTE for a doctor in your hospital, you can count this physician as 60/48 = 1.25 FTE<br><br>The term 'trainee not in residency training' refers to a clinician working in your hospital who is not qualified as a specialist, but is also not part of a formal training scheme towards becoming a specialist (neurosurgeon in this case).

17. How many neurologists (in FTE) work at your hospital?
……………….FTE neurologists
……………….FTE neurologist trainees in residency training
……………….FTE neurologist trainees not in residency training

The amount of FTEs do not have to be a whole number. If the amount of FTE is, for example, 3.3, please write down '3.3' here and not '3'!<br>If there are persons with out of hours work that is contracted and paid for, you can count them as > 1 FTE. For example, if there is a physician that is paid for 60 hours a week and 48 hours a week is considered as a FTE for a doctor in your hospital, you can count this physician as 60/48 = 1.25 FTE<br><br>The term 'trainee not in residency training' refers to a clinician working in your hospital who is not qualified as a specialist, but is also not part of a formal training scheme towards becoming a specialist (neurologist in this case)

18. How many trauma surgeons (in FTE) work at your hospital?
……………….FTE trauma surgeons
……………….FTE trauma surgeon trainees in residency training
……………….FTE trauma surgeon trainees not in residency training

The amount of FTEs do not have to be a whole number. If the amount of FTE is, for example, 3.3, please write down '3.3' here and not '3'!<br>If there are persons with out of hours work that is contracted and paid for, you can count them as > 1 FTE. For example, if there is a physician that is paid for 60 hours a week and 48 hours a week is considered as a FTE for a doctor in your hospital, you can count this physician as 60/48 = 1.25 FTE<br><br>The term 'trainee not in residency training' refers to a clinician working in your hospital who is not qualified as a specialist, but is also not part of a formal training scheme towards becoming a specialist (trauma surgeon in this case)<br><br>In this response, the term 'trauma surgeon' refers to an individual who specializes in trauma surgery, not a general surgeon or orthopaedic surgeon who happens to perform damage control surgery as part of wider responsibilities

19. How many intensivists (in FTE) work at the Intensive Care Unit (ICU) in your hospital? (all ICUs together if you have multiple):
……………….FTE intensivists
……………….FTE intensivist trainees in residency training
……………….FTE intensivist trainees not in residency training

The amount of FTEs do not have to be a whole number. If the amount of FTE is, for example, 3.3, please write down '3.3' here and not '3'!<br>If there are persons with out of hours work that is contracted and paid for, you can count them as > 1 FTE. For example, if there is a physician that is paid for 60 hours a week and 48 hours a week is considered as a FTE for a doctor in your hospital, you can count this physician as 60/48 = 1.25 FTE<br>The term 'trainee not in residency training' refers to a clinician working in your hospital who is not qualified as a specialist, but is also not part of a formal training scheme towards becoming a specialist (intensivist in this case)

20. How many Emergency Department (ED) physicians (in FTE) work at your ED?
……………………FTE ED physicians
……………………FTE trainees in residency training
……………………FTE trainees not in residency training

The amount of FTEs do not have to be a whole number. If the amount of FTE is, for example, 3.3, please write down '3.3' here and not '3'!
If there are persons with out of hours work that is contracted and paid for, you can count them as > 1 FTE. For example, if there is a physician that is paid for 60 hours a week and 48 hours a week is considered as a FTE for a doctor in your hospital, you can count this physician as 60/48 = 1.25 FTE

The term 'trainee not in residency training' refers to a clinician working in your hospital who is not qualified as a specialist, but is also not part of a formal training scheme towards becoming a specialist (ED physician in this case)

Payments

21. What is the system of funding that supports your hospital?
   - o Government / Public
   - o Private (includes both for-profit and non-profit institutions)

For the purposes of this question:
A government or public hospital is one that is owned by the government and receives government funding.
A private hospital is owned by a commercial organization or a non-profit organization and privately funded through payment for medical services by patients themselves, by health care insurers, or by the government through national health insurance schemes.

22. How are doctors who treat Traumatic Brain Injury (TBI) patients paid in your hospital?
   - o Doctors have a fixed monthly salary
   - o Fee for services (Doctors are paid per activity; for example, per operation, per investigation, per hospital day, etc.)
   - o Doctors are paid per patient (they receive a fixed amount of money per diagnosis)
   - o Doctors have a fixed monthly salary and are paid an extra fee for services
   - o Other, please specify____

This question seeks information on all doctors that treat TBI patients in your hospital, such as ED physicians, trauma surgeons, neurologists, neurosurgeons and intensivists.

If there is a difference between doctors, please select other and explain what doctors receive what form of payment. For example: "Intensivists and trauma surgeons have a fixed monthly salary, neurosurgeons are paid per operation"

A standard salary supplement for out of hours work, either resident in hospital or on call, should be interpreted as part of a fixed salary rather than extra fee for service

23. Does the salary of the doctor depend on a patient's insurance coverage?
   - o No
   - o Yes
   - o NA. all inhabitants in our country are insured

For example: If a patient is uninsured or insured by a particular insurance company, the doctor may receive less payment for the same operation in comparisons to patients who are insured or insured by another insurance company

24. Do doctors (or the department) receive additional payments for treatment of "private" patients and/or for patients who opt for extra facilities such as a single room?
   - o No
   - o Yes

"additional payments" do not need to be paid directly to the doctor as part of his/her personal salary, but may be paid into a departmental or personal fund which could include, for example, reimbursement of professional costs, including attendance at conferences, travel, dinner expenses etc.

Equipment

25. Do you have an electronic patient record in your hospital as a whole (not just confined to the ICU)?
- o No
- o Yes

"Electronic patient record" refers to a system that stores all patient information (for example laboratory values, CT scans, observatory notes, letters to the GP) electronically and not in a paper format.

26. Do you have an electronic data system in your Intensive Care Unit (ICU)?
- o No
- o Yes

Please notice: this question is only related to your ICU and not to your hospital as a whole (see previous question)

27. Is a Computed Tomography (CT) technician/radiographer available 24/7 to perform a CT scan?
- o No.
- o Yes. There is 24/7 in-house availability of a resident CT technician/radiographer to perform CT scans
- o Yes. CT technician/radiographers are on call and would arrive within 30 minutes to perform CT scans
- o Yes. CT technician/radiographers are on call, but would only arrive after 30 minutes to perform CT scans

28. Is a Magnetic Resonance Imaging (MRI) technician/radiographer available 24/7 to perform a MRI scan?
- o No.
- o Yes. There is 24/7 in-house availability of a resident MRI technician/radiographer to perform MRI scans
- o Yes. MRI technician/radiographers are on call and would arrive within 30 minutes to perform MRI scans
- o Yes. MRI technician/radiographers are on call, but would only arrive after 30 minutes to perform MRI scans

29. Is S100B routinely determined as a prognostic biomarker for neurological deterioration?
- o No
- o Yes

Hospital Costs

30. What are the average daily reimbursement costs for a severe Traumatic Brain Injury (TBI) patient in your hospital?

…………………euro

Reimbursement costs are the total costs that can be claimed from the insurance provider/the patient/public or government funds<br><br>If your country uses another monetary unit than the Euro, please calculate as Euros using a current exchange rate!

31. What are the average reimbursement costs of an Emergency Department visit?

………… euro

Reimbursement costs are the total costs that can be claimed from the insurance provider/the patient/public or government funds<br><br>If your country uses another monetary unit than the Euro, please calculate as Euros using a current exchange rate!

32. What are the average reimbursement costs of one day hospitalization at the ward?

……………euro

Reimbursement costs are the total costs that can be claimed from the insurance provider/the patient/public or government funds<br><br>If your country uses another monetary unit than the Euro, please calculate as Euros using a current exchange rate!

33. What are the average reimbursement costs one day hospitalization at the Intensive Care Unit (ICU)? (excluding the coronary care unit)?

……………..euro

A coronary care unit refers to a unit or some ICU beds that are specialized in patients with acute heart or coronary diseases. Reimbursement costs are the total costs that can be claimed from the insurance provider/the patient/public or government funds.<br><br>If your country uses another monetary unit than the Euro, please calculate as Euros using a current exchange rate!

34. What are the reimbursement costs for a routine clinical (not research) CT scan of the head for an ambulant patient in your hospital?
……………………..euro

Reimbursement costs are the total costs that can be claimed from the insurance provider/the patient/public or government funds<br><br>If your country uses another monetary unit than the Euro, please calculate as Euros using a current exchange rate!

35. What are the reimbursement costs for a routine clinical (not research) MRI scan of the head for an ambulant patient in your hospital?
……………………euro

Reimbursement costs are the total costs that can be claimed from the insurance provider/the patient/public or government funds<br><br>If your country uses another monetary unit than the Euro, please calculate as Euros using a current exchange rate!

36. What are the reimbursement costs for an ICP monitoring catheter in your hospital?
…………… euro for a parenchymal ICP monitoring catheter (device only)
…………… euro for ventricular catheter (device only)

Reimbursement costs are the total costs that can be claimed from the insurance provider/the patient/public or government funds<br><br>If your hospital never use respectively a parenchymal or ventricular catheter, you can type NA in the boxes.<br><br>If your country uses another monetary unit than the Euro, please calculate as Euros using a current exchange rate!

37. What is the average fulltime (1FTE) gross yearly base salary for a:

- Newly qualified nurse ………………Euro
- Senior nurse in charge of a ward during shifts ……………Euro

- Senior nurse in charge of an intensive care during shifts ………….Euro
- First year neurosurgery trainee in residency program: …………………………. Euro
- Final year neurosurgery trainee in residency program:……………………………… Euro
- First year intensive care trainee in residency program…………………..Euro
- Final year intensive care unit trainee in residency program…………………..Euro
- First year emergency medicine trainee in residency program…………………..Euro
- Final year emergency medicine trainee in residency program…………………..Euro

Please provide us the base salary only (without compensation fees, bonuses etc).<br>With a senior nurse in charge of a ward/ICU during shifts we refer to a senior nurse who is in charge of respectively the ward and the ICU during a part of the day (morning shift, night shift), but not in managerial charge of the ward/ICU as an organization.<br>A first year trainee in residency program is someone who finished medical school and just started with the training towards becoming a specialist. There may be differences among different specialisms (trauma surgeon, neurologist), so please give us an estimate here.<br>The last year trainee in residency program is someone who is almost ready with the residency program and who will become a specialist (eg neurosurgeon, trauma surgeon) in the next year.<br>If your country uses another monetary unit than the Euro, please calculate as Euros using a current exchange rate!

38. Where did you find this information?
……………………………………………………..

Name the source: for example annual report, registry

Acute Trauma care

39. Is there an in-hospital multidisciplinary team which will be alerted if a serious trauma victim comes in?

- ☐ No
- ☐ Yes

Some hospitals have a multidisciplinary team that is called when a serious trauma victim is expected. The function is early triage and treatment

40. Do you have a helicopter platform for helicopter trauma medical services?

- ☐ No
- ☐ Yes

40b. . How often is the helicopter platform approximately used per month?
…………. times

Teleconsulting and transfer

41. Which specialist usually makes the decision about transfer of Traumatic Brain Injury (TBI) patients to your hospital?

- ☐ Intensivist
- ☐ Neurosurgeon
- ☐ Trauma surgeon
- ☐ General or orthopaedic surgeon
- ☐ Neurologist
- ☐ Other, please specify…………………………………………..

This is the specialist who is often contacted by telephone or teleconsult and who decides that a TBI patient in another hospital will be transferred to your hospital<br><br>In this response, the term ‘trauma surgeon’ refers to an individual who specializes in trauma surgery, not a general surgeon or orthopaedic surgeon who happens to perform damage control surgery as part of wider responsibilities.

Provider Profiling Questionnaire

Questionnaire 2: Medical Ethics

The questionnaire can be completed by the CENTER-TBI Local investigator

The Local investigator is the senior clinician(s) at your hospital involved in supervision of CENTER TBI
For the completion of this questionnaire, we advise you to ask help from a data manager, administrative staff member and/or someone from the financial department in your hospital, since we ask for hospital data in this questionnaire. It is very important that this information is accurate, and searched for in annual reports, registries and other data sources rather than estimated.

This questionnaire also includes questions about the general policy in your hospital. The responses to these questions should represent, as best as practicable, a general consensus on treatment at your centre, rather than individual management preferences. Consequently, you should provide responses that describe not what you would do personally, but how the majority of patients would generally be treated in your centre.

There are no 'right' or 'wrong' answers so please give us a realistic and honest view of how the care in your hospital is organized. Your answers will only be used to answer the scientific questions in CENTER TBI and no information in any form will be reported on individual centre level

If you have any questions or problem, please contact:
Maryse Cnossen, PhD student (m.c.cnossen@erasmusmc.nl)

Information about the completer of the questionnaire

Other than the CENTER-TBI investigator, which of the following individuals was involved in completion of this questionnaire?

Tick all that apply

- ☐ Neurologist
- ☐ Neurosurgeon
- ☐ Trauma Surgeon
- ☐ Emergency Department (ED) physician
- ☐ Administrative staff member / data manager / financial department
- ☐ Other, please specify……………..
- ☐ NA. The questionnaire was completed soley by the CENTER TBI local investigator

Medical Ethics

1.Does your institute have a department of medical ethics?

- o No
- o Yes
- o Unknown

With a department of medical ethics we mean an academic department of ethics and philosophy or medicine, and not an office for institutional reviews of research documents. The department of medical ethics stands for research and education in medical ethics and philosophy of medicine. Usually the ethicists and faculty members in the department collaborate with clinicians on topics in different research areas in biomedical ethics

2.Is a medical ethicist available in your institution for consultation on patient care?

- o No
- o No, but a cleric (for example a priest, spiritual care) with ethical knowledge
- o No, but a physician with ethical expertise
- o Yes, a certified medical ethicist (involved in ethics consultation, research and education)
- o Yes, but only for deliberation on end-of-life issues
- o Other, please specify………………………………………………

3.Does your country have a *central* ethics committee for evaluation of research proposals?

- o No
- o Yes
- o Unknown

3b.If yes, what type of studies should be submitted to this committee?
Tick all that apply

- ☐ Phase I clinical trials (healthy volunteers)
- ☐ Phase II clinical trials testing experimental therapeutic interventions
- ☐ Phase III clinical trials testing therapeutic interventions
- ☐ Observational studies in mentally incapacitated patients
- ☐ Observational studies in emergency situations
- ☐ Intervention studies in mentally incapacitated patients
- ☐ Therapeutic studies (e.g. involving drugs) in children
- ☐ Non-therapeutic studies in children
- ☐ Studies involving genetics

- ☐ Studies involving vaccines

4.Can an Institutional Review Board (IRB) approval which is obtained in one centre be used to facilitate approval of submissions in other participating centres in your country?

- No
- Yes

An IRB (Institutional review board) is a multidisciplinary committee that evaluates research protocols and provides a binding judgment on whether the project is permissible on ethical grounds

4b.If yes,

- Yes, approval will follow without detailed evaluation
- Yes, but additional detailed evaluation is required
- Unknown

5.Does your IRB make any distinction in decision making for studies on acutely mentally incapacitated patients (e.g. TBI, stroke, cardiac arrest) or chronically mentally incapacitated patients (e.g. serious psychiatric disorders, dementia)?

- No
- Yes
- Unknown

6.Is there a consistent IRB policy for evaluations and approvals of studies in your country or are there to your knowledge variations between regions/cities/centres?

- Consistent policy
- Mainly consistent but there may be some variations
- Substantial variations exist between different states or cities
- Large variations exist strongly dependant on local IRB's

7.How many IRB applications have you / your department submitted over the past five years?

- Zero
- 1-2
- 3-5
- > 5
- Unknown

8.How many IRB applications have you / your department submitted over the past five years for studies of patients with acute cerebral disorders (involving mentally incapacitated patients in emergency situations)?

- Zero
- 1-2
- 3-5
- > 5
- Unknown

8b.Did you obtain approval in all submissions?

- No
- Yes
- Unknown

9.What is the average turnover time in your centre for IRB evaluations?

- 1 month
- 2 months

- 3 months
- 4 months
- 5 months
- 6 months
- > 6 months

10.Is proxy consent by a close relative considered valid by your IRB in acutely mentally incapacitated patients (e.g. TBI)?

- No
- Yes
- Unknown

11.Is proxy consent by a close relative considered valid by your IRB in chronic mentally incapacitated patients (e.g. psychiatric patients)?

- No
- Yes
- Unknown

12.Is clinical research involving mentally incapacitated patients in emergency situations regulated by law in your country?

- Yes, it is incorporated in national law
- No, it is not regulated by law
- No, it is not regulated by law, but we follow European Union regulations
- No, but we follow Good Clinical Practice or Declaration of Helsinki guidelines
- No, it is not regulated by law, but IRBs have the authority to approve
- Other, please specify……………………………………………
- I don't know

13.Can consent by waived or deferred in emergency clinical research in your **country**?

- Yes, this is regulated by law
- Yes, after prior approval of an IRB
- No
- I don't know

14.Is proxy consent by family members considered valid for mentally incapacitated patients in your **country**?

- No
- Yes
- Unknown

15.Is consent by an independent physician for participation of a mentally incapacitated patient considered valid in your **country**?

- No
- Yes
- Unknown

16.Do you have experience with obtaining IRB approval for emergency research involving mentally incapacitated patients?

- No
- Yes

16b.If yes, have the IRBs in your **country** made objections to emergency research involving mentally incapacitated patients without prior consent of patient or legal representatives?

- No
- Sometimes
- Yes

16c.If the IRB objected to emergency research involving mentally incapacitated patients without prior patient- or proxy consent, on which grounds or argumentation?

- It is against the law in this country
- Only a judge or other official juridical organ can approve such kind of research
- Only a patient him/herself can consent for inclusion in a study
- Prior written consent of a legal representative is always necessary before inclusion in a study
- Other, please specify ……………………………………

17.Do you think that emergency research involving mentally incapacitated patients without prior patient- or proxy consent is ethically feasible?

- No
- Yes

The responses to this question should represent, as best as practicable, a general consensus, rather than your personal opinion

17b.If yes, on which grounds?

- The emergency nature of the situation and possible harm to the patient
- Obtaining consent from relatives in distress is not valid
- Valuable possible therapeutic time is lost with the obligation to obtain consent

Other, please specify………………

Provider Profiling Questionnaire

Questionnaire 3: Pre-hospital Trauma Care

This questionnaire can be completed by an emergency physician

For the completion of this questionnaire, we advise you to ask help from a data manager, administrative staff member and/or someone from the financial department in your hospital, since we ask for hospital data in this questionnaire. It is very important that this information is accurate, and searched for in annual reports, registries and other data sources rather than estimated.

This questionnaire also includes questions about the general policy in your hospital. The responses to these questions should represent, as best as practicable, a general consensus on treatment at your centre, rather than individual management preferences. Consequently, you should provide responses that describe not what you would do personally, but how the majority of patients would generally be treated in your centre.

There are no 'right' or 'wrong' answers so please give us a realistic and honest view of how the care in your hospital is organized. Your answers will only be used to answer the scientific questions in CENTER TBI and no information in any form will be reported on individual center level.

If you have any questions or problem, please contact:

Maryse Cnossen, PhD student (m.c.cnossen@erasmusmc.nl)

Information about the completer of the questionnaire

Other than the CENTER-TBI investigator, which of the following individuals was involved in completion of this questionnaire?

Tick all that apply

- ☐ Neurologist
- ☐ Neurosurgeon
- ☐ Trauma Surgeon
- ☐ Emergency Department (ED) physician
- ☐ Administrative staff member / data manager / financial department
- ☐ Other, please specify…………….
- ☐ NA. The questionnaire was completed solely by the CENTER TBI local investigator

The Local investigator is the senior clinician(s) at your hospital involved in supervision of CENTER TBI

Provider profiling: Pre-hospital Trauma Care

General Public

1.Are first aid training initiatives available for the general public in the region?

Select all that apply

- ☐ No first aid training initiatives
- ☐ Obligatory for selected groups such as students, taxi drivers, (public) transport drivers
- ☐ Obligatory for the majority of the population
- ☐ Only for interested persons
- ☐ Don't know

2.Have there been information campaigns about first aid after trauma in the media in your region during the last 12 months?

Select all that apply

- ☐ No information campaigns in the media
- ☐ On television
- ☐ Radio
- ☐ Newspaper
- ☐ Posters
- ☐ Flyers/brochures
- ☐ Magazines
- ☐ Others …………………………………………….
- ☐ Don't know

3.Have there been information campaigns about cardiac resuscitation in the media in your region during the last 12 months?

Select all that apply

- ☐ No information campaigns in the media
- ☐ On television
- ☐ Radio
- ☐ Newspaper
- ☐ Posters
- ☐ Flyers/brochures
- ☐ Magazines
- ☐ Others …………………………………………….

- ☐ Don't know

Dispatch system

4.Is there a central telephone number for all emergency services or are separate calls required for ambulance, fire brigade and police?

- o Central number. Please specify number: …………………………………….
- o No central telephone access

5.Which system is used for dispatching ambulances?

- o Automatic dispatching (ambulance stationary on post closest to the scene is mobilized)
- o Dynamic ambulance management (ambulance which is vacant closest to the scene is mobilized)
- o Selective dispatching (depends on the nature and urgency of the reported incidence).
- o Others: ………………………….

6.What type of training is required for the dispatching personnel in your country?

- o No special training
- o Nursing
- o Specialized training for medical dispatching
- o Medical training
- o Nursing + specialized training
- o Others ……………………………..

7.Can a medical mobile team/MERIT team (with emergency physician or anesthetist) be dispatched 24/7 in your region? (Not obligatory a hospital-based team)

- o Always
- o Often
- o Occasionally
- o Never

8.What are the criteria for sending a mobile medical team/ MERIT team (with emergency physician) to the scene?
Select all that apply

- ☐ At each road traffic accident
- ☐ The presumption of central nervous damage
- ☐ Severe bleeding
- ☐ Low Glasgow coma scale / Unresponsive patient
- ☐ Respiratory distress
- ☐ Entrapment
- ☐ Major incident/multiple casualties
- ☐ The presumption of cardiac arrest
- ☐ Sports trauma
- ☐ Elderly person with fall incident
- ☐ Others: …………………………………………
- ☐ Not applicable (no MMT available)

9.Is a helicopter routinely used in the region of your hospital to transport the MMT to the scene of the incident?

- o Always
- o Often

- Occasionally
- Never

10.Is a helicopter <u>routinely</u> used in the region of your hospital to transport the patient to the hospital?

- Always
- Often
- Occasionally
- Never

11.If a helicopter is used, on which indications?
Select all that apply

- ☐ At each road traffic accident
- ☐ The presumption of central nervous damage
- ☐ Severe bleeding
- ☐ Unresponsive patient
- ☐ Low Glasgow coma scale
- ☐ Respiratory distress
- ☐ Entrapment
- ☐ Major incident/multiple casualties
- ☐ The presumption of cardiac arrest
- ☐ Sports trauma
- ☐ Elderly person with fall incident
- ☐ Expectation of prolonged transport time on land
- ☐ specify: cut off point = ……. minutes
- ☐ Others: ………………………………………

12.Who is allowed to make the decision whether a mobile medical team or helicopter with emergency doctor will be sent to the scene?

- ☐ The dispatcher
- ☐ The ambulance at the scene
- ☐ A doctor at the scene
- ☐ The first responders
- ☐ Trauma cell (a team of professionals who are specialized in trauma care)
- ☐ N/A we do not have a mobile medical team and/or helicopter service
- ☐ Other ………………………………………….

<u>Emergency services</u>

13.What is the specialism of the emergency physician on the MMT or helicopter service?
Select all that apply

- ☐ Anesthesiologist
- ☐ Orthopedic surgeon
- ☐ General surgeon
- ☐ Trauma surgeon
- ☐ Neuro surgeon
- ☐ Emergency physician
- ☐ General practitioner
- ☐ Cardiologist
- ☐ Other: …………………………..

14.What is the basic type of response which is send to the trauma scene if there is no indication for a MMT/MERIT?

- ☐ Basic Life Support (BLS) crew
- ☐ Advanced Life Support (ALS) crew
- ☐ Crew without medical training
- ☐ Other: …………………………………………

15.What type of training is required for the ambulance personnel?

- o Nursing with additional training in critical care
- o Nursing
- o Training in Basic Life Support – first aid
- o Emergency Medical Technician – basic
- o Emergency Medical Technician - paramedic
- o No training
- o Others …………………………………………

16. Do the ambulance drivers receive specialized training?

- o Specialized driving skills training
- o Specialized driving skills training and the required training for ambulance personnel
- o Specialized driving skills training and Basic Life Support training
- o Only the required training for ambulance personnel
- o No training
- o Others ……………………………………………………………………………………………………….

17.What is the target time between the call for help to the emergency services and arrival on scene (the general target time, if categorized the response time for category A (most urgent)?

- o No target time
- o ≤ 5 minutes
- o ≤ 10 minutes
- o ≤ 15 minutes
- o ≤ 20 minutes
- o ≤ 25 minutes
- o ≤ 30 minutes
- o Others: …………………

18.Which actions may be performed by the ambulance personnel in the basic type of response? *Select all that apply*

- ☐ transportation of the patient
- ☐ BLS, sustain vital functions without specialized medical equipment
- ☐ Administering medication
- ☐ Intubation without drugs
- ☐ Placing chest drainage
- ☐ Infusion of fluids
- ☐ Cardioversion (automatic defibrillator – manual defibrillator) (underline which one)
- ☐ Preparation of medical interventionsl (infusions, medication …)
- ☐ Others ……………………………………………

19.What is the policy at the scene of trauma?

- o Always Scoop and run: the point of focus is the speed of response and transport, the aim is to transport the patient as fast as possible to hospital
- o Always Stay and play: the point of focus is to initiate primary treatment and stabilize the patient before transport
- o Mostly scoop and run, stay and play on indication
- o Mostly stay and play, scoop and run on indication

- Others ……………………………………………………………………………………………

20.Which method is used to evaluate the neurological status of the patient at the scene?
Select all that apply

- ☐ Glasgow coma scale
- ☐ AVPU (Alert Voice Pain Unresponsive)
- ☐ ACDU (Alert Confused Drowsy Unresponsive)
- ☐ Simplified motor score: response of the patient is characterized in obeys command, localizes pain, withdrawal or lesser response to pain
- ☐ FOUR score
- ☐ RLS85
- ☐ Other: ……………

21.What is the target time working at the scene to stabilize the patient for transport?

- No target time
- ≤5 minutes
- ≤10 minutes
- ≤15 minutes
- ≤20 minutes
- ≤25 minutes
- ≤30 minutes
- Others: ……………

22.What is the target time between stabilization on scene and arrival at the ED?

- No target time
- ≤5 minutes
- ≤10 minutes
- ≤15 minutes
- ≤20 minutes
- ≤25 minutes
- ≤30 minutes
- Others: …………………

Hospital reception and treatment

23.To which center is the patient transferred?

- Mostly to the hospital closest to the scene
- Mostly to a trauma center/ major hospital
- Selected patient population is sent to the trauma center, the others are sent to the closest hospital

24.How are patients selected for transport to the trauma center/major hospital?

- ☐ Structured approach to triage using
- ☐ Revised Trauma Score (RTS) under 11 – 10 – 9 – 8 (circle the correct one)
- ☐ Glasgow Coma Sum Score (GCS): cut off value: ……. (3-15); (if scored lower, patient is sent to trauma center)
- ☐ Others …………………………………………………………..
- ☐ Preference of patient or family
- ☐ Choice of ambulance personnel
- ☐ Choice of doctor at the scene
- ☐ Others ……………………………………

25.Which method is used to evaluate the neurological status of the patient at ED admission? (Tick all that apply)

- ☐ Glasgow coma scale
- ☐ AVPU
- ☐ ACDU
- ☐ Simplified motor score
- ☐ FOUR score

- RLS85
- Others …………………………………………

26.Are ATLS principles implemented and followed in the ED?
- Yes
- No

27.Which discipline is in charge when a multi-trauma patient arrives at the ED?
- Anesthesiologist
- Orthopedic surgeon
- General surgeon
- Trauma surgeon
- Neuro surgeon
- Emergency physician
- Nursing staff
- Pediatric
- Others ………………………………

28. Is a consultant specialist present within 5 minutes of arrival of a major trauma victim?
- Always
- Only during normal working hours
- Seldom
- never

Provider Profiling Questionnaire

Questionnaire 4: Emergency Department (ED)

This questionnaire can be completed an ED physician

For the completion of this questionnaire, we advise you to ask help from a data manager, administrative staff member and/or someone from the financial department in your hospital, since we ask for hospital data in this questionnaire. It is very important that this information is accurate, and searched for in annual reports, registries and other data sources rather than estimated.

This questionnaire also includes questions about the general policy in your hospital. The responses to these questions should represent, as best as practicable, a general consensus on treatment at your centre, rather than individual management preferences. Consequently, you should provide responses that describe not what you would do personally, but how the majority of patients would generally be treated in your centre.

There are no 'right' or 'wrong' answers so please give us a realistic and honest view of how the care in your hospital is organized. Your answers will only be used to answer the scientific questions in CENTER TBI and no information in any form will be reported on individual centre level. Some of the questions may seem similar, but please answer all questions.

If you have any questions or problem, please contact:
Maryse Cnossen, PhD student (m.c.cnossen@erasmusmc.nl)

Information about the completer of the questionnaire

Other than the CENTER-TBI investigator, which of the following individuals was involved in completion of this questionnaire?

Select all that apply

- ☐ Neurologist
- ☐ Neurosurgeon
- ☐ Trauma Surgeon
- ☐ Emergency Department (ED) physician
- ☐ Administrative staff member / data manager / financial department
- ☐ Other, please specify……………..
- ☐ NA. The questionnaire was completed solely by the CENTER TBI local investigator

The Local investigator is the senior clinician(s) at your hospital involved in supervision of CENTER TBI

General

1.How many acute resuscitation rooms (resuscitation beds) do you have in your Emergency Department (ED) ?

………………………

2. Do you have a special facility for overnight observation (this does not refer to admission for observation or overnight stay in a normal inpatient ward)?

- o No
- o Yes

2b. If yes: how many observation beds do you have?

…………………………………………..

3. What is the maximal observation time in this facility?

- o ≤ 6 hours
- o 7 - 12 hours
- o 13 - 24 hours
- o Overnight
- o Other, please specify………………….

4. Does your hospital have separate 24/7 emergency operation rooms?

- o No
- o Yes

The response to this question should address operating rooms that are exclusively used for emergency surgery, and **<u>not</u>** used for planned or elective surgery

5. How many Emergency Department (ED) physicians (in FTE) work at your ED?

……………………FTE ED physicians

……………………FTE trainees in residency training

……………………FTE trainees not in residency training

FTE = Full time equivalent. '1 FTE' may be constituted by one person who works on a fulltime basis, but can also refer to two persons who work half-time.

The amount of FTEs do not have to be a whole number. If the amount of FTE is, for example, 3.3, please write down '3.3' here and not '3'!
If there are persons with out of hours work that is contracted and paid for, you can count them as > 1 FTE. For example, if there is a physician that is paid for 60 hours a week and 48 hours a week is considered as a FTE for a doctor in your hospital, you can count this physician as 60/48 = 1.25 FTE

The term 'trainee not in residency training' refers to a clinician working in your hospital who is not qualified as a specialist, but is also not part of a formal training scheme towards becoming a specialist (ED physician in this case)

6. What is the total number of Emergency Department (ED) visits in your hospital annually?
2012: ……………………………………..
2013: ……………………………………..

The response to this question should include all ED attendees – not just patients with TBI

7. What is the total number of Traumatic Brain Injury (TBI) patients (all severities) visiting the Emergency Department (ED) in your hospital annually?
2012: ……………………………………..
2013: ……………………………………..

8. Where did you find this information?
……………………………………………………….

Name the source: for example annual report, registry

Definition of mild, moderate and severe Traumatic Brain Injury (TBI) in your hospital

9. What Glasgow Coma Scale (GCS) scores are considered as mild, moderate and severe TBI in your hospital?

	Lowest GCS value	Highest GCS values
Mild TBI	…………………..	…………………..
Moderate TBI	…………………..	…………………..
Severe TBI	…………………..	…………………..

There are differences between countries and hospitals in how they classify mild, moderate and severe TBI. Please give the lowest and highest GCS values that you consider as mild, moderate and severe. For example: severe TBI might have a lowest value of 3 and a highest value of 8.
You can use hospital or national guidelines here. If these are not available, we would recommend, for example, an email exchange with colleagues to check that the answer that you provide us here represents the view of most of the persons in your department.

This questionnaire includes many questions that are specifically about mild, moderate and/or severe TBI. Please ensure that you answer these questions in the context of TBI severity specified, and based on the GCS classification you have provided here.

Acute trauma care

10. Are severe Traumatic Brain Injury (TBI) patients usually directly transferred to your hospital or most often indirectly after initial resuscitation and/or early Computed Tomography (CT) elsewhere?

- Directly
- Indirectly
- Both

Direct transfer = when a patient is directly transferred from the place where the accident occurred to your hospital. A patient is not seen in another hospital before he/she is referred to your hospital
Secondary transfer = when a patient is first seen in another hospital. The patients is transferred from the place where the accident occurred to another hospital. After this, the patient is referred to your hospital. This can occur directly after arrival in the other hospital or after some treatment and diagnostics.

This question refers to the majority of TBI patients, recognizing that there may be exceptions. You can read this as: how would >75% of the severe TBI patients be referred to your hospital.

Only select both if both direct and secondary transfer for severe TBI patients can be mentioned as general policy.

11. Is there an in-hospital multidisciplinary team which will be alerted if a serious trauma victim comes in?

- o No
- o Yes

Some hospitals have a multidisciplinary team that is alerted when a serious trauma victim is expected. The function is early triage and treatment

11b. If the response to the previous question is yes:
What specialties are standard represented in the trauma team (present upon reception of the patient)?

	Staff member	Trainee (not) in residency training
ED physician	☐	☐
Anesthesiologist	☐	☐
Trauma surgeon	☐	☐
Neurologist	☐	☐
Neurosurgeon	☐	☐
General or orthopaedic surgeon	☐	☐
Radiologist	☐	☐
Orthopaedic	☐	☐
Other, please specify …………………………………………..	☐	☐

This question refers to the specialties that are routinely part of the trauma team and not to the specialties that are consulted if necessary. The members are physically present upon reception.
In this response, the term 'trauma surgeon' refers to an individual who specializes in trauma surgery, not a general surgeon or orthopaedic surgeon who happens to perform damage control surgery as part of wider responsibilities.

Imaging

These questions are about mild traumatic brain injury only! See question 9 for your centre specific definition of mild TBI

Computed Tomography (CT) scan

12. Are (inter)national or local guidelines used to determine which mild Traumatic Brain Injury (TBI) patients should have an initial head CT in your Emergency Department (ED)?

- ☐ We do not use guidelines for determining who should get an initial CT
- ☐ NICE guidelines
- ☐ Canadian CT head rule
- ☐ New Orleans criteria

- ☐ CHIP rule
- ☐ Scandinavian guidelines for initial management of minimal, mild and moderate head injury
- ☐ Other international guideline, please specify …………………………………………………..
- ☐ Other national guideline, please specify………………………………………………………….
- ☐ Other local, regional or hospital guideline, please specify ……………………………………………
- ☐ No guideline

12b. If you selected other local, regional or hospital guideline:
If available, please send a pdf/internet link of your protocol/guideline

13. How are guidelines and protocols regarding CT scanning implemented at your Emergency Department (ED)?
Select all that apply

- ☐ No formal implementation of guidelines
- ☐ Verbal direction from clinical managers/ clinical directors/senior doctors
- ☐ Written protocols and algorithms
- ☐ Training organized by an external organisation
- ☐ Training organized by your own hospital / department
- ☐ E-learning
- ☐ flowchart / algorithms / protocols in the patient data management system of your ED
- ☐ Periodic feedback on adherence to the guideline
- ☐ Structural attention for protocol adherence during clinical rounds Other (please specify) ……………………………………………………………………………….

14. Is there a group or individuals who oversee guideline development and maintenance at your Emergency Department (ED)?

- ☐ Neither
- ☐ Individual
- ☐ Group
  - o Single discipline: ED physicians / trauma surgeons / neurosurgeons / neurologists (please circle correct response)
  - o Multidisciplinary
- ☐ N/A. Guidelines are not implemented at our ED

15. Have there been audits to check for adherence to guidelines at your Emergency Department (ED)?

- o Not in the last five years
- o Once in the last five years
- o Approximately 2-4 times in the last five years
- o On a yearly basis
- o Several times a year
- o N//A. Guidelines are not implemented at our ED

An audit is a process by which your hospital / ED assesses how well guidelines are followed

16. How do you consider the adherence to the CT guidelines at your Emergency Department (ED)?

- o Guidelines are used in (almost) no cases (0-25%)
- o Guidelines are used in some cases (25-50%)

- Guidelines are used in most cases (50-75%)
- Guidelines are used in (almost) all cases (75-100%)
- N/A. Guidelines are not implemented at our ED

The responses to this question should represent, as best as practicable, a general consensus, rather than your personal opinion.

If CT guidelines are implemented at your ED (Question 12 is not "We do not use guidelines for determining who should get an initial CT":
17. What do you judge as being the reasons for nonadherence to CT guidelines in patients with mild Traumatic Brain Injury (TBI)?

	Never (0-10%)	Rarely (10-30%)	Sometimes (30-70%)	Frequently (70-90%)	Always (90-100%)
Lack of knowledge among clinicians about CT scan guidelines	☐	☐	☐	☐	☐
Every patient is unique; whether a CT scan needs to be performed should be managed by clinical judgment rather than by a guideline	☐	☐	☐	☐	☐
Inadequate time to consult CT guidelines for urgent decisions	☐	☐	☐	☐	☐
Guidelines on TBI do not apply due to extracranial trauma or comorbidity	☐	☐	☐	☐	☐
Inadequate resources to apply guidelines (no CT scanner available, lack of personnel)	☐	☐	☐	☐	☐
Defensive medicine leads to performing a CT scan even if not required by the guidelines*	☐	☐	☐	☐	☐
Other, please specify………………	☐	☐	☐	☐	☐

*Defensive medicine refers to the practice of performing a CT scan that is not necessarily the best option for the patient, but an option that mainly serves the function to protect the physician against the patient as potential plaintiff.

The responses to this question should represent, as best as practicable, a general consensus, rather than your personal opinion.

If CT guidelines are implemented at your ED (Question 12 is not "We do not use guidelines for determining who should get an initial CT"):
18. Who answered the above mentioned questions about adherence to guidelines?

- ED physician
- ED trainee in residency training
- Trauma surgeon
- Email exchange in multidisciplinary ED team
- Other, please specify…………………………………

19. In which of the following situations would you perform a CT scan in a <u>mild</u> Traumatic Brain Injury (TBI) patient?

This question is about indications in which you would perform a CT scan in a patient with mild TBI.

Select NEVER in factors considered not important in the treatment decision whether someone should get a CT scan.

Select ONLY IN THE PRESENCE OF OTHER RISK FACTORS if the factor is never solely a reason for a CT scan, but it might be a reason in combination with one or more other risk factors. For example: a hospital may consider headache, intoxication and the use of anticoagulant drugs in isolation as risk factors that are not sufficient to perform a CT scan. However, if these present together, their combined presence might constitute an indication for CT scanning. Respondents from such a hospital should tick 'only in the presence of other risk factors' after headache, intoxication and the use of anticoagulant drugs.

Select OFTEN / PARTIAL is the risk factor is often seen as a reason for CT scanning in your hospital. However, it is not general practice, because not everyone in your hospital agrees or CT scanning is only general policy in a subset of the patients. For example, it might be general policy to scan patients aged over 70 when presenting to your ED, but not patients aged 60-70. You can complete age => 60 with OFTEN/PARTIAL.

Select ALWAYS/GENERAL POLICY when the criteria are, in general, a reason for CT scanning in your hospital (>75% of the patients with this indication). When you select ALWAYS/GENERAL POLICY this must represent a general consensus among colleagues, rather than individual preference.
Where you are in doubt whether this is the appropriate response to the question, we would recommend, for example, either a verbal discussion or an email exchange with colleagues to check consensus.

	Never	Only in the presence of other risk factors	Often / Partial	Always / General Policy
Prior loss of consciousness	☐	☐	☐	☐
Headache	☐	☐	☐	☐
Vomiting	☐	☐	☐	☐
Age >=60	☐	☐	☐	☐

Any anticoagulant therapy (not including anti-platelet therapy)	☐	☐	☐	☐
Any antiplatelet therapy (not including anticoagulant therapy)	☐	☐	☐	☐
Use of SSRI drugs	☐	☐	☐	☐
Intoxication (alcohol / drugs)	☐	☐	☐	☐
Seizure	☐	☐	☐	☐
Vulnerable road user (pedestrian or cyclist)	☐	☐	☐	☐
Fall from any elevation	☐	☐	☐	☐
PTA >= 4 hours	☐	☐	☐	☐
Alternation of consciousness	☐	☐	☐	☐
Any neurologic deficit	☐	☐	☐	☐
Clinical signs of fracture skull base or vault	☐	☐	☐	☐
Physical evidence of trauma to head / skull	☐	☐	☐	☐
Signs of facial fracture	☐	☐	☐	☐
Contusion of the face	☐	☐	☐	☐
In children: suspicion of non-accidental injury	☐	☐	☐	☐
Increased serum levels of S100B	☐	☐	☐	☐

20. Does your Emergency Department (ED) – in general- have a liberal or restrictive policy regarding CT scanning?

- ○ Liberal
- ○ Restricted
- ○ Unknown

> The responses to this question should represent, as best as practicable, a general consensus on treatment at your centre, rather than individual management preferences.

21. What percentage of all mild Traumatic Brain Injury (TBI) patients attending at your Emergency Department (ED) do get a CT scan? Can you give us an estimate?

- ………….%
- Unknown

The responses to this question should represent, as best as practicable, a general consensus, rather than individual thoughts.

22. Who generally orders the CT for patients with mild TBI in your Emergency Department (ED)?
Select one answer here.

- ED physician
- Neurologist
- Neurosurgeon
- (Trauma)surgeon
- Other, please specify………………………………

The responses to this question should represent your general policy. You can read this question as: Who would order a CT scan in >75% of the mild TBI patients at your ED.<br>If there are multiple persons ordering a CT scan in the majority of patients, select other and then list the physicians who order a CT scan in >75%

23. Is your Emergency Department (ED) more restrictive in performing CT examinations in children with Traumatic Brain Injury (TBI) than in adults?

- No
- Yes
- Unknown

The responses to this question should represent, as best as practicable, a general consensus on treatment at your centre, rather than individual management preferences.

Magnetic Resonance Imaging (MRI) scan

24. What are indications for MRI scanning in patients with Traumatic Brain Injury (TBI)?
Select all that apply

- ☐ Discrepancy between clinical symptomatology and (lack of) CT abnormalities
- ☐ Suspicion non-metal foreign object
- ☐ Instead of CT (limiting radiation exposure)
- ☐ Suspicion of spinal cord lesion
- ☐ Other, please specify …………………………………………………

The responses to this question should represent, as best as practicable, a general consensus on treatment at your centre, rather than individual management preferences.

25. How often is the MRI scanner used as primary investigation (instead of the CT scanner) in patients with (suspected) Traumatic Brain Injury (TBI)?

- Never (0-10%)
- Rarely (10-30%)
- Sometimes (30-70%)
- Frequently (70-90%)
- Always (90-100%)

The responses to this question should represent, as best as practicable, a general consensus on treatment at your centre, rather than individual management preferences.

Consultation

26. When one wants to consult a specialist for patients with TBI, what specialty is most often consulted in the following situations?
Select one specialist in every severity level.

	Neurosurgeon	Neurologist	Trauma Surgeon	Other specialist. Please specify ……..	No consultation
Mild TBI	☐	☐	☐	☐	☐
Moderate TBI	☐	☐	☐	☐	☐
Severe TBI	☐	☐	☐	☐	☐
Any polytrauma patient, irrespective of Glasgow Coma Scale (GCS) score	☐	☐	☐	☐	☐

See question 9 for your centre specific definition of mild, moderate and severe TBI.<br>Select the specialist that is in most (>75%) of the patients consulted. When there is no consultation in most of the patients, select no consultation.<br>Consultation refers to a situation in which the specialist physically examines the patient and provides an advice about further treatment, imaging, admission, and/or discharge.<br><br>In this response, the term 'trauma surgeon' refers to an individual who specializes in trauma surgery, not a general surgeon or orthopaedic surgeon who happens to perform damage control surgery as part of wider responsibilities.

27. Is S100B routinely determined as a prognostic biomarker for neurological deterioration?
- No
- Yes

Laboratory turnaround time

28. What maximum laboratory turnaround times are recorded in the lab Standard Operating Procedures (SOP) at your Emergency Department (ED) for a severely injured patient?
- 15 minutes
- 15-20 minutes
- 20-30 minutes
- 30-45 minutes
- 45-60 minutes
- More than 60 minutes
- ☐ NA. There is no lab SOP that determines the maximum laboratory turnaround time for severely injured patients

Note here the time that is recorded in the SOP and not the average actual time

Management of Emergency Department overcrowding

Overcrowding is defined as a situation in which there are more patients in the ER than the ER can handle (due to lack of beds, lack of personnel, access block etc)

29. How often does it occur that patients are placed in the hallway?

- Multiple times a day
- Approximately once a day
- On a weekly basis
- On a monthly basis
- Seldom
- It hasn't occurred in the last five years

30. How often does it occur that it takes > 2 hours to get to a ward once the decision to admit has been made (access block)?

- Multiple times a day
- Approximately once a day
- On a weekly basis
- On a monthly basis
- Seldom
- It hasn't occurred in the last five years

31. What is the average number of patients per week leaving the Emergency Department (ED) without being seen or treated?

…….

32. What is the average time until triage at your Emergency Department (ED) (including all presenting diagnoses)?

…………

33. Is overcrowding considered as a problem in your Emergency Department (ED)?

- No. Never
- Sometimes
- We consider ED overcrowding as a frequent problem in our ED

The response to this question could include the following considerations:

- Ability of ambulances to offload patients,
- Number of patients who leave without being seen or treated,
- Time until triage,
- Frequency of ED occupancy rate >100%,
- Time until physician first see a patient,
- ED boarding time,
- Number of patients boarding in the ED,
- Lab turn-around times,
- Time to imaging.

The responses to this question should represent, as best as practicable, a general consensus, rather than an individual opinion.

34. What are the rates for ambulance diversion?

Can you give an estimate of the last year:

- Never
- <6/year
- 6/year – 1/month
- 1/month – 1/week.
- 1/week – 2/week
- > 2/week

Ambulance diversion refers to a situation in which an ambulance that arrived at the hospital has to go to another hospital in the area as a result of overcrowding.

Anticoagulation

These questions are about <u>mild</u> traumatic brain injury only. (See question 9 for your centre specific definition of mild TBI)

35. In patients with <u>mild</u> Traumatic Brain Injury (TBI), oral anticoagulation is reversed:
Select all that apply
- ☐ in all patients irrespective of presence of CT abnormalities
- ☐ in patients with demonstrated CT abnormalities
- ☐ if surgery is considered/indicated
- ☐ NA. Oral anticoagulation is never reversed in mild TBI patients

36. Coagulopathy is treated with:
Select all that apply
- ☐ FFP
- ☐ Platelets
- ☐ Fibrinogen
- ☐ Novo 7 (recombinant factor VII)
- ☐ Vitamin K
- ☐ PCC (Prothrombin Complex Concentrate)
- ☐ Other, please specify…………….

The responses to this question should represent, as best as practicable, a general consensus on treatment at your centre, rather than individual management preferences.

Hospital admission after Emergency Department (ED)

37. Do you use guideline/protocols to decide whether patients with <u>mild</u> Traumatic Brain Injury (TBI) should be admitted to hospital?
- ○ No
- ○ Yes

See question 10 for your centre specific definition of mild TBI

38. In which of the following situations would you admit a patient with <u>mild</u> Traumatic Brain Injury (TBI) to the hospital ward?
Please provide us the general clinical practice at your centre. This does not have to be the same as stated in the guidelines you use

This question is about indications for hospital ward admission in patients with mild TBI.<br><br>See question 9 for your centre specific definition of mild TBI.<br><br>Select NEVER in factors considered not important in the decision whether mTBI patients should be admitted to the ward.<br><br>Select ONLY IN THE PRESENCE OF OTHER RISK FACTORS if the factor is never solely a reason for ward admission, but it might be a reason in combination with one or more other risk factors. For example: a hospital may consider severe headache and drugs or alcohol intoxication in isolation as risk factors that are not sufficient to admit a patient to the ward. However, if these present together, their combined presence might be considered an indication. Respondents from such a hospital should tick 'only in the presence of other risk factors' after severe headache and drugs or alcohol intoxication.

Select OFTEN / PARTIAL is the risk factor is often seen as a reason for ward admission in your hospital. However, it is not general practice, because not everyone in your hospital agrees or admission is only general policy in a subset of the patients. For example, it might be general policy to admit patients with drugs intoxication to the ward, but not those with alcohol intoxication. You can complete 'drugs or alcohol intoxication' with OFTEN/PARTIAL

Select ALWAYS/GENERAL POLICY when the criteria are, in general, a reason for ward admission in your hospital. When you select ALWAYS/GENERAL POLICY this must represent a general consensus among colleagues, rather than individual preference. Where you are in doubt whether this is the appropriate response to the question, we would recommend, for example, either a verbal discussion or an email exchange with colleagues to check consensus.

	Never	Only in the presence of other risk factors	Often / Partial	Always / General Policy
Patients with new, clinically significant abnormalities on imaging	☐	☐	☐	☐
Computed Tomography (CT) progression	☐	☐	☐	☐
Patients whose Glasgow Coma Scale (GCS) score has not returned to 15 after imaging, regardless of the imaging results	☐	☐	☐	☐
When a patient has indications for CT scanning but this cannot be done within the appropriate period, either because CT is not available or because the patient is not sufficiently cooperative to allow scanning	☐	☐	☐	☐
Persistent vomiting	☐	☐	☐	☐
Severe headaches	☐	☐	☐	☐
Clinician is concerned (without specific reason)	☐	☐	☐	☐
Drugs or alcohol intoxication	☐	☐	☐	☐
Other injuries	☐	☐	☐	☐
Shock (hypotension/tachycardia)	☐	☐	☐	☐
Suspected non-accidental injury	☐	☐	☐	☐

Meningism	☐	☐	☐	☐
Cerebrospinal fluid leak	☐	☐	☐	☐
Patient or family demands it	☐	☐	☐	☐
There is no responsible adult available to check on the patient regularly	☐	☐	☐	☐
TBI as a result of a suicide attempt	☐	☐	☐	☐
Preinjury anticoagulation	☐	☐	☐	☐
Preinjury antiplatelets	☐	☐	☐	☐
Homeless patients	☐	☐	☐	☐
Planned surgery	☐	☐	☐	☐
	☐	☐	☐	☐
Other, please specify…………….	☐	☐	☐	☐

39. When would you admit moderate Traumatic Brain Injury (TBI) to the hospital ward (exclude moderate TBI patients in which ICU admission is indicated)?

- o Never
- o Only in the presence of other risk factors (like premorbid anticoagulant use, older age, CT progression)
- o It is our general policy to admit all moderate TBI patients to the hospital ward (based on Glasgow Coma Scale)

> The responses to this question should represent, as best as practicable, a general consensus on treatment at your centre, rather than individual management preferences.

40. In which of the following situations would you admit a patient with moderate Traumatic Brain Injury (TBI) to the Intensive Care Unit (ICU)?
Please provide us the general clinical practice at your centre. This does not have to be the same as stated in the guidelines you use

> This question is about indications for ICU admission in patients with moderate TBI.
> See question 9 for your centre specific definition of moderate TBI.
>
> Select NEVER in factors considered not important in the decision whether moderate TBI patients should be admitted to the ICU
>
> Select ONLY IN THE PRESENCE OF OTHER RISK FACTORS if the factor is never solely a reason for ICU admission, but it might be a reason in combination with one or more other risk factors. For example: a hospital may consider CT progression, persistent vomiting and severe headache in isolation as risk factors that are not sufficient to admit a patient to the ICU. However, if these present together, their combined presence might be considered an indication. Respondents from such a hospital should tick 'only in the presence of other risk factors' after CT progression, persistent vomiting and headache.

Select OFTEN / PARTIAL is the risk factor is often seen as a reason for ICU admission in your hospital. However, it is not general practice, because not everyone in your hospital agrees or admission is only general policy in a subset of the patients. For example, it might be general policy to admit patients intoxicated with drugs to the ICU but not patients intoxicated with alcohol. Respondents from such a hospital should tick 'often/partial' in 'drugs or alcohol intoxication'.

Select ALWAYS/GENERAL POLICY when the criteria are, in general, a reason for ICU admission in your hospital. When you select ALWAYS/GENERAL POLICY this must represent a general consensus among colleagues, rather than individual preference. Where you are in doubt whether this is the appropriate response to the question, we would recommend, for example, either a verbal discussion or an email exchange with colleagues to check consensus.

	Never	Only in the presence of other risk factors	Often / Partial	Always / General Policy
All moderate TBI patients (according to their Glasgow Coma Scale (GCS) score)	☐	☐	☐	☐
Patients with new, clinically significant abnormalities on imaging	☐	☐	☐	☐
CT progression	☐	☐	☐	☐
Patients whose GCS has not returned to 15 after imaging, regardless of the imaging results	☐	☐	☐	☐
When a patient has indications for CT scanning but this cannot be done within the appropriate period, either because CT is not available or because the patient is not sufficiently cooperative to allow scanning	☐	☐	☐	☐
Persistent vomiting	☐	☐	☐	☐
Severe headaches	☐	☐	☐	☐
Clinician is concerned (without specific reason)	☐	☐	☐	☐
Drugs or alcohol intoxication	☐	☐	☐	☐
Shock (hypotension/tachycardia)	☐	☐	☐	☐
Meningism	☐	☐	☐	☐
Cerebrospinal fluid leak	☐	☐	☐	☐

Patient or family demands it	☐	☐	☐	☐
Other……………..	☐	☐	☐	☐

Discharge home

41. Do you use protocols/guidelines to decide when patients with <u>mild</u> Traumatic Brain Injury (TBI) are discharged from the Emergency Department (ED) ?

- No
- Yes

42. Is printed discharge information available in the Emergency Department (ED) to hand out to patients who are discharged?

- No
- Yes

43. What discharge information is routinely given verbally and/or written to the patient upon discharge?

	Verbally	Written
Details of the nature and severity of the injury	☐	☐
Symptoms that prompt patients to return for consultation	☐	☐
Details about the recovery process, including the fact some Patients may appear to make a quick recovery but later experience difficulties or complication	☐	☐
Contact details of community and hospital services in case of Delayed complication	☐	☐
Information about return to everyday activities, including school, work, sports and driving	☐	☐
Information about post concussion syndrome/persisting symptoms and what to do in this situation	☐	☐
Information about the use of pain killers and other medication	☐	☐
Details of support organization	☐	☐
Other, please specify……………………………………………………………… …………………………………………………………………………………………	☐	☐

What kind of follow-up treatment is scheduled when someone with mild Traumatic Brain Injury (TBI) is discharged home after Emergency Department (ED)?

Select all that apply

- ☐ No scheduled follow-up.
- ☐ Routinely scheduled outpatient follow up after …. Weeks/days
- ☐ Referred to general practitioner (regardless of persisting symptoms)
- ☐ The patient is advised to contact the general practitioner if symptoms persist

- ☐ The patient is advised to come back to the hospital if symptoms persist
- ☐ Other, please specify

Outcome

44. Does your hospital routinely assess the outcome at follow-up according to the Glasgow Outcome Scale (extended)?
   - o No
   - o Yes
   - o Unknown

44b. If yes: Is a structural interview used to assess the Glasgow Outcome Scale (extended)?
   - o No
   - o Yes
   - o Unknown

44c. If yes: Who usually assesses the GOS(E)?
Select all that apply
   - ☐ Research nurse
   - ☐ Nurse
   - ☐ Clinician
   - ☐ Other, please specify….

45. Which of the following reasons for disability does your hospital include or exclude in your assessment of the GOS(E)?

In the responses below, you can choose either “include” or “Exclude” as a response to each item. If you tick “Include” means that you assess all of the disability as part of categorizing the patient on the GOSE. If you tick “Exclude” this means that where the disability is thought to be the consequence of an injury other than TBI, it will not be included in the assessment, and you would assign the GOS(E) as if that disability did not exist.

	Include	Exclude
Effects of health conditions that existed before the injury, such as cognitive impairment or physical limitations.	☐	☐
Effects of injuries sustained on the same occasion to parts of the body other than the head, such as paralysis due to spinal cord injury or injuries to the limbs.	☐	☐
Effects of external damage to the head or injury to the skull, such as limitations in activities due to a missing bone flap.	☐	☐
Effects of illness arising after TBI treatment, such as pulmonary complications after ventilation.	☐	☐
Effects of a subsequent illness unconnected to TBI, such as pneumonia after flu.	☐	☐
Effects of a subsequent operation, such as hip replacement, that is unconnected to TBI.	☐	☐
Effects of changed social circumstances, such as lower income after injury.	☐	☐
Effects of depression that has arisen since the TBI.	☐	☐
Effects of post-traumatic stress disorder that has appeared since	☐	☐

the TBI.

Effects of post-injury anxiety states, such as the development of agoraphobia. ☐ ☐

Withdraw life support

46. Occasionally, in patients with severe trauma, the presence of an irretrievable intracranial injury may prompt a decision not to continue with active therapy. In these cases, how is the decision reached to withhold/withdraw life-sustaining measures (e.g. mechanical ventilation, vasoactive medication, CVVH, intravenous fluid administration)?

Select all that apply

- ☐ Based on objective medical criteria (as GCS, age, comorbidity)) during multidisciplinary deliberation in which one physician (veto, for example the most senior person or the ED director) has to agree
- ☐ Based on objective medical criteria (as GCS, age, comorbidity) during multidisciplinary deliberation in which the majority (more than 50%) has to agree
- ☐ Based on objective medical criteria (as GCS, age, comorbidity) during multidisciplinary deliberation in which there has to be unanimous consensus among all participating doctors
- ☐ Based on subjective opinion (among which objective medical criteria) of a senior physician
- ☐ Based on opinions and objective medical criteria (as GCS, age, comorbidity) during multidisciplinary deliberation in which the majority (more than 50%) has to agree
- ☐ Based on opinions and objective medical criteria (as GCS, age, comorbidity) during multidisciplinary deliberation in which there has to be unanimous consensus among all participating doctors
- ☐ We never withdraw treatment in the ED
- ☐ Other, please specify ……………………………………………………….

47. How is a decision reached to not treat patients surgically, because the primary brain damage is considered too devastating (poor prognosis)?

Select all that apply

- ☐ Based on objective medical criteria (as GCS, age, comorbidity) by one physician (veto)
- ☐ Based on objective medical criteria (as GCS, age, comorbidity) during multidisciplinary deliberation in which the majority (more than 50%) has to agree
- ☐ Based on objective medical criteria (as GCS, age, comorbidity) during multidisciplinary deliberation in which there has to be unanymous consensus among all participating doctors
- ☐ Based on subjective opinion (among which objective medical criteria) of one physician (veto)
- ☐ Based on opinions and objective medical criteria (as GCS, age, comorbidity) during multidisciplinary deliberation in which the majority (more than 50%) has to agree
- ☐ Based on opinions and objective medical criteria (as GCS, age, comorbidity) during multidisciplinary deliberation in which there has to be unanymous consensus among all participating doctors
- ☐ Other, please specify …………………………………………..

48. If the patient ‘appears’ brain dead (GCS 3, fixed dilated pupils, apnea), do you:

Select all that apply

- ☐ Stop all life-sustaining measures on the ED
- ☐ Arrange transfer to the ICU for further observation
- ☐ Arrange transfer to the ICU for possible organ donation

49. Do you admit very elderly (80 years and older) patients with severe Traumatic Brain Injury (TBI) on the ICU for treatment?

Select all that apply

- ☐ No, never
- ☐ Yes, if the patient is intubated and ventilated in the ED setting
- ☐ Yes, if the patient needs ICU treatment with the prospect of saving his/her life
- ☐ Yes, but highly depending on the severity of co-morbidity
- ☐ Yes, but only if the relatives ask me

Provider Profiling Questionnaire

Questionnaire 5: Admission

This questionnaire can be completed by a neurologist, neurosurgeon or another doctor familiar with the hospital ward

The topic of this questionnaire is admission. Admission refers to staying for at least one night at the hospital ward. We do NOT refer to staying at the Intensive Care Unit (ICU) here (see ICU questionnaire) or staying in the observation unit for one night (see Emergency Department questionnaire).

For the completion of this questionnaire, we advise you to ask help from a data manager, administrative staff member and/or someone from the financial department in your hospital, since we ask for hospital data in this questionnaire. It is very important that this information is accurate, and searched for in annual reports, registries and other data sources rather than estimated.

This questionnaire also includes questions about the general policy in your hospital. The responses to these questions should represent, as best as practicable, a general consensus on treatment at your centre, rather than individual management preferences. Consequently, you should provide responses that describe not what you would do personally, but how the majority of patients would generally be treated in your centre. Some of the questions may seem similar, but please answer all questions.

There are no 'right' or 'wrong' answers so please give us a realistic and honest view of how the care in your hospital is organized. Your answers will only be used to answer the scientific questions in CENTER TBI and no information in any form will be reported on individual centre level

If you have any questions or problem, please contact:
Maryse Cnossen, PhD student (m.c.cnossen@erasmusmc.nl)

Information about the completer of the questionnaire

Other than the CENTER-TBI investigator, which of the following individuals was involved in completion of this questionnaire?
Select all that apply

- ☐ Neurologist
- ☐ Neurosurgeon
- ☐ Trauma Surgeon
- ☐ Emergency Department (ED) physician
- ☐ Administrative staff member / data manager / financial department
- ☐ Other, please specify…………….
- ☐ NA. The questionnaire was completed solely by the CENTER TBI local investigator

The Local investigator is the senior clinician(s) at your hospital involved in supervision of CENTER TBI

General

1.To what hospital ward(s) are patients with Traumatic Brain Injury (TBI) who do not require Intensive Care Unit (ICU) care <u>most often</u> admitted?
Select all that apply

- ☐ Neurological hospital ward
- ☐ General surgery hospital ward
- ☐ Trauma surgery hospital ward
- ☐ Neurosurgical hospital ward
- ☐ Other ward, please specify…………………………………….

Please answer the following questions about the ward(s) that you selected here.

2. Do you have an electronic patient record in your hospital as a whole (not just confined to the ICU)?

- o No
- o Yes

"Electronic patient record" refers to a system that stores all patient information (for example laboratory values, CT scans, observatory notes, letters to the GP) electronically and not in a paper format.

Definition of mild, moderate and severe Traumatic Brain Injury (TBI) in your hospital

3. What Glasgow Coma Scale (GCS) scores are considered as mild, moderate and severe Traumatic Brain Injury (TBI) in your hospital:

	Lowest GCS value	Highest GCS values
Mild TBI	…………………..	…………………..
Moderate TBI	…………………..	…………………..
Severe TBI	…………………..	…………………..

There are differences between countries and hospitals in how they classify mild, moderate and severe TBI. Please give the lowest and highest GCS values that is considered as mild, moderate and severe. For example: severe TBI might have a lowest value of 3 and a highest value of 8.<br><br>You can use hospital or national guidelines here. If these are not available, we would recommend, for example, an email exchange with colleagues to check that the answer that you provide us here represents the view of most of the persons in your department.

4. In which of the following situations would you admit a patient with mild Traumatic Brain Injury (TBI) to the hospital ward?

Please provide us the general clinical practice at your centre. This does not have to be the same as stated in the guidelines you use

This question is about indications for hospital ward admission in patients with mild TBI.
See the above question for your centre specific definition of mild TBI.

Select NEVER in factors considered not important in the decision whether mTBI patients should be admitted to the ward.

Select ONLY IN THE PRESENCE OF OTHER RISK FACTORS if the factor is never solely a reason for ward admission, but it might be a reason in combination with one or more other risk factors. For example: a hospital may consider severe headache and drugs or alcohol intoxication in isolation as risk factors that are not sufficient to admit a patient to the ward. However, if these present together, their combined presence might be considered an indication. Respondents from such a hospital should tick 'only in the presence of other risk factors' after severe headache and drugs or alcohol intoxication.

Select OFTEN / PARTIAL is the risk factor is often seen as a reason for ward admission in your hospital. However, it is not general practice, because not everyone in your hospital agrees or admission is only general policy in a subset of the patients. For example, it might be general policy to admit patients with drugs intoxication to the ward, but not those with alcohol intoxication. You can complete 'drugs or alcohol intoxication' with OFTEN/PARTIAL

Select ALWAYS/GENERAL POLICY when the criteria are, in general, a reason for ward admission in your hospital. When you select ALWAYS/GENERAL POLICY this must represent a general consensus among colleagues, rather than individual preference. Where you are in doubt whether this is the appropriate response to the question, we would recommend, for example, either a verbal discussion or an email exchange with colleagues to check consensus.

	Never	Only in the presence of other risk factors	Often / Partial	Always / General Policy
Patients with new, clinically significant abnormalities on imaging	☐	☐	☐	☐
Computed Tomography (CT) progression	☐	☐	☐	☐
Patients whose Glasgow Coma Scale (GCS) score has not returned to 15 after imaging, regardless of the imaging results	☐	☐	☐	☐
When a patient has indications for CT scanning but this cannot be done within the appropriate period, either because CT is not available or because the patient is not sufficiently cooperative to allow scanning	☐	☐	☐	☐
Persistent vomiting	☐	☐	☐	☐

Severe headaches	☐	☐	☐	☐
Clinician is concerned (without specific reason)	☐	☐	☐	☐
Drugs or alcohol intoxication	☐	☐	☐	☐
Other injuries	☐	☐	☐	☐
Shock (hypotension/tachycardia)	☐	☐	☐	☐
Suspected non-accidental injury	☐	☐	☐	☐
Meningism	☐	☐	☐	☐
Cerebrospinal fluid leak	☐	☐	☐	☐
Patient or family demands it	☐	☐	☐	☐
There is no responsible adult available to check on the patient regularly	☐	☐	☐	☐
TBI as a result of a suicide attempt	☐	☐	☐	☐
Preinjury anticoagulation	☐	☐	☐	☐
Preinjury antiplatelets	☐	☐	☐	☐
Homeless patients	☐	☐	☐	☐
Planned surgery	☐	☐	☐	☐
	☐	☐	☐	☐
Other, please specify…………….	☐	☐	☐	☐

5. Would you admit patients with isolated <u>moderate</u> Traumatic Brain Injury (TBI) to the hospital ward (exclude moderate TBI patients in which ICU admission is indicated)?

- Never
- Only in the presence of other risk factors (like premorbid anticoagulant use, older age, CT progression)
- It is our general policy to admit all moderate TBI patients to the hospital ward (based on GCS)

The responses to this question should represent, as best as practicable, a general consensus on treatment at your centre, rather than individual management preferences.

Guidelines

6. Are guidelines / protocols implemented for patients with Traumatic Brain Injury (TBI) at your hospital ward (for example about CT scanning, timing of discharge, neurological examination)?

- o No, we do not have guidelines for TBI patients
- o We do not have specific guidelines for TBI patients, but we do have other guidelines that are applied to TBI patients (e.g. neurological examination guidelines, acquired brain injury guidelines)
- o Yes, we do have specific guidelines for TBI patients

If available, please send a pdf / internet link of your protocol(s)

Observation

7. How often is the Glasgow Coma Scale (GCS) usually assessed at the hospital ward

- o Half-hourly for 2 hours, then 1-hourly for 4 hours; then 2-hourly
- o Less often. Please specify your scheme:…………………………………………………………………………………………………..
- o More often. Please specify your scheme:…………………………………………………………………………………………………..
- o Other, Please specify your scheme……………………………………………………………………….

8. Is posttraumatic amnesia systematically assessed at your hospital ward?

- o No
- o Yes, Galveston amnesia and orientation test
- o Yes, other approach. Please specify……………………………………………………………………………

Routinely repeated Computed Tomography (CT) scans

9. Are routinely repeated CT scans used in patients with Traumatic Brain Injury (TBI) at your hospital ward?

Select all that apply

- ☐ No
- ☐ Yes in mild TBI patients
- ☐ Yes in moderate TBI patients
- ☐ Yes in severe TBI patients

With routine repeat CT scan we mean CT scans that are performed at predetermined time points regardless of clinical symptoms.

This question refers to the general policy in your hospital. You can read this as: would you perform routine repeat CT scans in the majority (>75%) of the patients?

See for the definition of mild, moderate and severe TBI your answer on question 3.

9b. If yes: What are indications for routine repeat Computed Tomography (CT) scans in Traumatic Brain Injury (TBI):

Select all that apply

- ☐ For all TBI patients that are admitted to the ward, routine repeat CT scans are scheduled
- ☐ Any abnormality on initial CT scan
- ☐ Glasgow coma scale < 15

- ☐ Substance abuse prior to the TBI
- ☐ Patient on anticoagulants/antiplatelet agents
- ☐ Other, please specify…………………………………………………………………………………………

The responses to this question should represent, as best as practicable, a general consensus on treatment at your centre, rather than individual opinions.

9c. If routine repeat CT scans are scheduled, do you have a protocol of how often and over what time period patients are scanned?
- o No
- o Yes

9d. If yes: please specify the time period
Scan 2: …………………………… hours after initial CT scan
Scan 3:. …………………………… hours after initial CT scan
Scan 4: …………………………… hours after initial CT scan
Etc.

Note here what is stated in the protocol.

10. Is S100B routinely determined as a prognostic biomarker for neurological deterioration?
- o No
- o Yes

Management of confused patients
11. What is the treatment policy in patients who show confusion:
Select all that apply
- ☐ Analgesics / pain killers
- ☐ Anxiolytics
- ☐ Antipsychotics (e.g. haloperidol)
- ☐ Restraints
- ☐ Verbal interaction
- ☐ Other, please specify…………………………..
- ☐ A combination, please specify……….

The responses to this question should represent, as best as practicable, a general consensus on treatment at your centre, rather than individual opinions.

Treatment
12. Anti-seizure prophylaxis is used in our centre:

Please first rank how often you use anti-seizure prophylaxis in TBI patients in general. You can choose between never, rarely, sometimes, frequently and always. The percentages can help you define what we mean by never till always. After that you can answer this question for subgroups of TBI patients.

The responses to this question should represent, as best as practicable, a general consensus on treatment at your centre, rather than individual management preferences.

	Never (0-10%)	Rarely (10-30%)	Sometimes (30-70%)	Frequently (70-90%)	Always (90-100%)
Glasgow Coma Scale score < 10	☐	☐	☐	☐	☐

Cortical contusion	☐	☐	☐	☐	☐
Depressed skull fracture	☐	☐	☐	☐	☐
Subdural hematoma	☐	☐	☐	☐	☐
Epidural hematoma	☐	☐	☐	☐	☐
Intracerebral hematoma	☐	☐	☐	☐	☐
Penetrating head wound	☐	☐	☐	☐	☐
Seizure within 24h of injury	☐	☐	☐	☐	☐
Other – please specify ……………………………….. …………………………………	☐	☐	☐	☐	☐

13. What is the general policy for a Traumatic Brain Injury (TBI) with an early seizure* ?

- Anti epileptic drugs are prescribed in all TBI patients with an early seizure
- Anti epileptic drugs are prescribed in TBI patients with an early seizure AND a CT abnormality
- We never prescribe anti epileptic drugs in TBI patients with an early seizure
- Other, please specify…………..

*Early posttraumatic epileptic insults are defined as seizures occurring within 7 days of trauma.

The responses to this question should represent, as best as practicable, a general consensus on treatment at your centre, rather than individual opinions.

14. What is the general policy regarding the use of antibiotics in patients who have CSF leak following Traumatic Brain Injury (TBI)?

- Routine antibiotics
- We only prescribe antibiotics when the patient has fever
- Watchful waiting*
- Other, please specify………………………….

Watchful waiting refers to an approach in which time is allowed to pass before a medical intervention (e.g. antibiotics) is used. During this time, repeated testing may be performed.

The responses to this question should represent, as best as practicable, a general consensus on treatment at your centre, rather than individual opinions.

Intermediate/Step-down Care:

An intermediate or step-down bed or a medium care facility is a facility in between the Intensive Care Unit (ICU) and the hospital ward. It is often used for patients who improved at the ICU and no longer need the intensivity of care delivered by the ICU, but are also not well enough for a routine hospital ward. The care provided at the stepdown beds/intermediate care unit is less intensive than the care provided at the ICU but more intensive than hospital ward care

15. Do you have step down beds / medium care facilities for patients with traumatic brain injury?
   - No
   - Yes

15b. If yes, how many step down beds do you have for patients with traumatic brain injury?
……………………………………….

16. What are the main reasons for medium care admission (instead of ward admission or ICU admission) in patients with isolated Traumatic Brain Injury (TBI)?
Select all that apply
- ☐ Decreased consciousness
- ☐ Intracranial complications
- ☐ To monitor vital functions
- ☐ Frequent GCS assessments
- ☐ Confusion
- ☐ Other, please specify………………………………….

17. What specialty is generally responsible for Traumatic Brain Injury (TBI) patients in the intermediate care or step down unit?
- Neurologist
- Intensivist
- General or orthopaedic surgeon
- Neurosurgeon
- Trauma surgeon
- ☐ Other, please specify……………………………….

In this response, the term 'trauma surgeon' refers to an individual who specializes in trauma surgery, not a general surgeon or orthopaedic surgeon who happens to perform damage control surgery as part of wider responsibilities

Discharge

18. Is printed discharge information available at the ward to hand out to patients discharged?
- No
- Yes

19. What discharge information is usually given verbally and/or written to the patient upon ward discharge:

	Verbally	Written
details of the nature and severity of the injury	☐	☐
symptoms that mean patients need to return for consultation	☐	☐
details about the recovery process, including the fact some patients may appear to make a quick recovery but later	☐	☐

experience difficulties or complication		
contact details of community and hospital services in case of delayed complication	☐	☐
information about return to everyday activities, including school, work, sports and driving	☐	☐
information about post concussion syndrome / persisting symptoms and what to do in this situation	☐	☐
Information about the use of pain killers and other medication	☐	☐
Details of support organization	☐	☐
Other, please specify………………………………………………………………<br>………………………………………………………………………………………	☐	☐

20. What kind of follow-up treatment is usually scheduled after ward admission for Traumatic Brain Injury (TBI):

- ☐ No scheduled follow-up.
- ☐ Routinely scheduled outpatient follow up after …. Weeks/days
- ☐ Referred to general practitioner (regardless of persisting symptoms)
- ☐ The patient is advised to contact the general practitioner if symptoms persist
- ☐ The patient is advised to come back to the hospital if symptoms persist
- ☐ Other, please specify………………………….

Outcome

21. Does your hospital routinely assess the outcome at follow-up according to the Glasgow Outcome Scale (extended)?

- o No
- o Yes
- o Unknown

21b. If yes: Is a structural interview used to assess the Glasgow Outcome Scale (extended)?

- o No
- o Yes
- o Unknown

21c. If yes: Who usually assesses the GOS(E)?
Select all that apply

- ☐ Research nurse
- ☐ Nurse
- ☐ Clinician
- ☐ Other, please specify….

22. Which of the following reasons for disability does your hospital include or exclude in your assessment of the GOS(E)?

In the responses below, you can choose either “include” or “Exclude” as a response to each item. If you tick “Include” means that you assess all of the disability as part of categorizing the patient on the GOSE. If you tick “Exclude” this means that where the disability is thought to be the consequence of an injury other than TBI, it will not be included in the assessment, and you would assign the GOS(E) as if that disability did not exist.

	Include	Exclude
Effects of health conditions that existed before the injury, such as cognitive impairment or physical limitations.	☐	☐
Effects of injuries sustained on the same occasion to parts of the body other than the head, such as paralysis due to spinal cord injury or injuries to the limbs.	☐	☐
Effects of external damage to the head or injury to the skull, such as limitations in activities due to a missing bone flap.	☐	☐
Effects of illness arising after TBI treatment, such as pulmonary complications after ventilation.	☐	☐
Effects of a subsequent illness unconnected to TBI, such as pneumonia after flu.	☐	☐
Effects of a subsequent operation, such as hip replacement, that is unconnected to TBI.	☐	☐
Effects of changed social circumstances, such as lower income after injury.	☐	☐
Effects of depression that has arisen since the TBI.	☐	☐
Effects of post-traumatic stress disorder that has appeared since the TBI.	☐	☐
Effects of post-injury anxiety states, such as the development of agoraphobia.	☐	☐

Provider Profiling Questionnaire

Questionnaire 6: Structural and organizational aspects of the Intensive Care Unit (ICU)

This questionnaire can be completed by a(n) (neuro)intensivist

For the completion of this questionnaire, we advise you to ask help from a data manager, administrative staff member and/or someone from the financial department in your hospital, since we ask for hospital data in this questionnaire. It is very important that this information is accurate, and searched for in annual reports, registries and other data sources rather than estimated.

This questionnaire also includes questions about the general policy in your hospital. The responses to these questions should represent, as best as practicable, a general consensus on treatment at your centre, rather than individual management preferences. Consequently, you should provide responses that describe not what you would do personally, but how the majority of patients would generally be treated in your centre.

There are no ‘right’ or ‘wrong’ answers so please give us a realistic and honest view of how the care in your hospital is organized. Your answers will only be used to answer the scientific questions in CENTER TBI and no information in any form will be reported on individual centre level. Some of the questions may seem similar, but please answer all questions.

If you have any questions or problem, please contact:
Maryse Cnossen, PhD student (m.c.cnossen@erasmusmc.nl)

Information about the completer of the questionnaire

Other than the CENTER-TBI investigator, which of the following individuals was involved in completion of this questionnaire?
Select all that apply

- ☐ Neurologist
- ☐ Neurosurgeon
- ☐ Trauma Surgeon
- ☐ Emergency Department (ED) physician
- ☐ Administrative staff member / data manager / financial department
- ☐ Other, please specify…………….
- ☐ NA. The questionnaire was completed solely by the CENTER TBI local investigator

The Local investigator is the senior clinician(s) at your hospital involved in supervision of CENTER TBI

General

1.How many Intensive Care Units (ICUs) (in separate locations / floors) does your hospital have (excluding ICUs that are exclusively for the coronary care)?
…………………………………………

Some hospitals may have one ICU where all severely ill patients are admitted to. Other hospitals may have different ICUs. Some patients (for example neurotrauma patients) are referred to one ICU while other patients (for example internal pathology) are referred to the other.<br>A coronary care unit refers to a unit or some ICU beds that are exclusively for patients with acute heart or coronary diseases

2. How many Intensive Care Unit (ICU) beds does your hospital have in total (excluding beds that are exclusively the coronary care)?
……………………………………..

3. Is there a fixed maximum number of beds available for neurological/neurosurgical patients at your Intensive Care Unit (ICU)?

- o No
- o Yes. If yes, how many beds? ………………………………….

4. How many patients can be ventilated at your Intensive Care Unit (ICU) at the same time?
……………………………………………….

5. Does your hospital have a dedicated neuro-intensive care?

- o No
- o Yes

5b. If yes, how many beds does the neuro-intensive care has?
………………………………………

If there is more than 1 Intensive Care Unit (ICU) in your hospital:
6. Are Traumatic Brain Injury (TBI) patients always admitted to the same ICU?

- o No
- o Yes
- o Not applicable, we have one ICU

If there is more than 1 Intensive Care Unit (ICU) in your hospital:
7. Is the admitting Intensive Care Unit (ICU) the same for patients with polytrauma Traumatic Brain Injury (TBI) as for isolated TBI?

- No
- Yes
- Not applicable, we have one ICU

Many patients with TBI suffer from polytrauma. For example patients who have been in a car accident are likely to have multiple problems and not only a head injury, in contrast to isolated TBI, were TBI is the only problem. Are these two patient categories treated at the same or separate ICUs?

8. Do you have step down beds/intermediate care facilities for patients with traumatic brain injury?

- No
- Yes

A step-down bed or a medium care facility is a facility in between the ICU and the hospital ward. It is often used for patients who improved at the ICU and no longer need the intensivity of ICU care, but are also not well enough to be cared for on a routine hospital ward. The care provided in stepdown/intermediate care beds is less intensive than the care provided at the ICU but more intensive than hospital ward care

8b. If yes, how many step down beds do you have?
………………………………………

9. What are the main reasons for intermediate care admission (instead of ward admission or ICU admission) in patients with isolated Traumatic Brain Injury (TBI) ?
Select all that apply

- ☐ Decreased consciousness
- ☐ Intracranial complications
- ☐ To monitor vital functions
- ☐ Frequent Glasgow Coma Scale (GCS) assessments
- ☐ Confusion
- ☐ Other, please specify………………………………

Staffing

10. How many intensivists (in FTE) work at your Intensive Care Unit (ICU) (all ICUs together if you have multiple):
……………………FTE Intensivists
……………………FTE Intensive care trainees in residency training
……………………FTE Intensive care trainees not in residency training

FTE = Full time equivalent. '1 FTE' may be constituted by one person who works on a fulltime basis, but can also refer to two persons who work half-time.<br><br>The amount of FTEs do not have to be a whole number. If the amount of FTE is, for example, 3.3, please write down '3.3' here and not '3'!<br>If there are persons with out of hours work that is contracted and paid for, you can count them as > 1 FTE. For example, if there is a physician that is paid for 60 hours a week and 48 hours a week is considered as a FTE for a doctor in your hospital, you can count this physician as 60/48 = 1.25 FTE

11. What is the intensivist-patient ratio at your Intensive Care Unit (ICU) during the day?
1: …………………………..

Record here the number of patients that are treated by one intensivist. If there are for example 3 intensivists working at the daily shift at your ICU and 9 beds, the ratio is 1:3.<br>During weekends/nights the ratio may be different, but this particular question seeks staffing numbers during an average daily shift.<br>If you have > 1 ICU in your hospital and the intensivist-patient ratios varies, please think of the ICU where severe TBI patients are most often referred to.

12. Is there 24/7 qualified intensivist coverage?

- o No
- o Yes. There is 24/7 resident availability of a qualified intensivist in the hospital
- o Yes. Qualified intensivists are on call at home and can attend the ICU within 30 minutes
- o Yes. Qualified intensivists are on call at home and can attend the ICU, but not within 30 minutes
- o Yes. There is 24/7 access to a qualified intensivist by telecommunication/phone. Qualified intensivists are however not able to attend the unit outside working hours

13. During the night, what is the most senior person <u>physically</u> present at your Intensive Care Unit (ICU)?

- o Trainee not in residency training
- o Trainee in residency training towards becoming ICU specialist
- o Trainee in residency training towards becoming a specialist other than intensivist
- o Fellow (specialist) in training for ICU
- o Certified intensivist / ICU physician
- o Other, please specify….

14. What is the average number of years work experience of this person?
……………………… years of ICU experience

15. How many nurses (in FTE) work at your Intensive Care Unit (ICU) (all ICUs together if you have multiple)?
…………………………………………..FTE

FTE = Full time equivalent. '1 FTE' may be constituted by one person who works on a fulltime basis, but can also refer to two persons who work half-time.<br><br>The amount of FTEs do not have to be a whole number. If the amount of FTE is, for example, 3.3, please write down '3.3' here and not '3'!<br>If there are persons with out of hours work that is contracted and paid for, you can count them as > 1 FTE. For example, if there is a nurse that is paid for 60 hours a week and 48 hours a week is considered as a FTE for a nurse in your hospital, you can count this physician as 60/48 = 1.25 FTE

16. What is the intensive care nurse-to-patient ratio at the Intensive Care Unit (ICU) during the day?
1:………………………..

Note here the number of patients that are treated by one nurse at night. If there are for example 9 nurses working at your ICU at the daily shift and 9 beds, the ratio is 1:1.<br>If you have > 1 ICU in your hospital and the nurse-patient ratios varies, please think of the ICU where severe TBI patients are most often referred to.

17. Do nurse-to-patient ratios vary with the time of the day?

- No. The nurse-patient ratio is always what you have filled in the previous question
- Yes it varies.

If yes: what is the nurse-to-patient ratio at night?
1: ……………………………..

Admission / Transfer

18. Is the admission policy to the Intensive Care Unit (ICU)influenced by shortage of ICU beds?

- Never
- Rarely (< 1/month)
- Sometimes (monthly)
- Frequently (weekly)
- Very frequently (at least a few times per week)

19. How often does it happen that patients are discharged from the Intensive Care Unit (ICU)/ transferred sooner than planned because of shortage of ICU beds?

- Never
- Rarely (< 1/month)
- Sometimes (monthly)
- Frequently (weekly)
- Very frequently (at least a few times per week)

Definition of mild, moderate and severe Traumatic Brain Injury (TBI) in your hospital

20. What Glasgow Coma Scale (GCS) scores are considered as mild, moderate and severe TBI in your hospital?

	Lowest GCS value	Highest GCS values
Mild TBI	…………………..	…………………..
Moderate TBI	…………………..	…………………..
Severe TBI	…………………..	…………………..

> There are differences between countries and hospitals in how they classify mild, moderate and severe TBI. Please give the lowest and highest GCS values that you consider as mild, moderate and severe. For example: severe TBI might have a lowest value of 3 and a highest value of 8.
> You can use hospital or national guidelines here. If these are not available, we would recommend, for example, an email exchange with colleagues to check that the answer that you provide us here represents the view of most of the persons in your department.

21. In which of the following situations would you admit a patient with <u>moderate</u> Traumatic Brain Injury (TBI) to the <u>Intensive Care Unit (ICU)?</u>
Please provide us the general clinical practice at your centre. This does not have to be the same as stated in the guidelines you use

> This question is about indications for ICU admission in patients with moderate TBI.
> See the question above for your centre specific definition of moderate TBI.
>
> Select NEVER in factors considered not important in the decision whether moderate TBI patients should be admitted to the ICU

Select ONLY IN THE PRESENCE OF OTHER RISK FACTORS if the factor is never solely a reason for ICU admission, but it might be a reason in combination with one or more other risk factors. For example: a hospital may consider CT progression, persistent vomiting and severe headache in isolation as risk factors that are not sufficient to admit a patient to the ICU. However, if these present together, their combined presence might be considered an indication. Respondents from such a hospital should tick 'only in the presence of other risk factors' after CT progression, persistent vomiting and headache.

Select OFTEN / PARTIAL if the risk factor is often seen as a reason for ICU admission in your hospital. However, it is not general practice, because not everyone in your hospital agrees or admission is only general policy in a subset of the patients. For example, it might be general policy to admit patients intoxicated with drugs to the ICU but not patients intoxicated with alcohol. Respondents from such a hospital should tick 'often/partial' in 'drugs or alcohol intoxication'.

Select ALWAYS/GENERAL POLICY when the criteria are, in general, a reason for ICU admission in your hospital. When you select ALWAYS/GENERAL POLICY this must represent a general consensus among colleagues, rather than individual preference. Where you are in doubt whether this is the appropriate response to the question, we would recommend, for example, either a verbal discussion or an email exchange with colleagues to check consensus.

	Never	Only in the presence of other risk factors	Often / Partial	Always / General Policy
All moderate TBI patients (according to their Glasgow Coma Scale (GCS) score)	☐	☐	☐	☐
Patients with new, clinically significant abnormalities on imaging	☐	☐	☐	☐
CT progression	☐	☐	☐	☐
Patients whose GCS has not returned to 15 after imaging, regardless of the imaging results	☐	☐	☐	☐
When a patient has indications for CT scanning but this cannot be done within the appropriate period, either because CT is not available or because the patient is not sufficiently cooperative to allow scanning	☐	☐	☐	☐
Persistent vomiting	☐	☐	☐	☐
Severe headaches	☐	☐	☐	☐
Clinician is concerned (without specific reason)	☐	☐	☐	☐
Drugs or alcohol intoxication	☐	☐	☐	☐

Shock (hypotension/tachycardia)	☐	☐	☐	☐
Meningism	☐	☐	☐	☐
Cerebrospinal fluid leak	☐	☐	☐	☐
Patient or family demands it	☐	☐	☐	☐
Other……………..	☐	☐	☐	☐

22. **Organization of your Intensive Care Unit (ICU)**
Which of the following best describes the model of ICU care at your hospital?
Please select one option only

- Closed ICU: critical care physicians (intensivists) assume primary responsibility for delivery of intensive care for Traumatic Brain Injury (TBI) patients. It is possible here that other specialists (e.g. a neurosurgeon) are consulted for advice when deterioration occurs. However, the intensivist remains responsible
- Open ICU: the admitting surgeon (neurosurgeon / trauma surgeon) assumes primary responsibility for care of TBI patients, including the provision of critical care services. This model of care may include elective consultation of an intensivist
- Mixed: the admitting surgeon (neurosurgeon / trauma surgeon) assumes primary responsibility for care of TBI patients. A certified physician in critical care (intensivists) coordinates the delivery of care

Decision making at your Intensive Care Unit (ICU)
23. Treatment decisions regarding neurosurgical interventions in Traumatic Brain Injury (TBI) patients in your Intensive Care Unit (ICU) are mainly determined by:

- Trauma surgeon
- Neurosurgeon
- General surgeon or orthopaedic surgeon
- Neurointensivist
- Neurologist
- General Intensivist

Only tick the person(s) that is responsible for the decision. It is possible that options are discussed in a multidisciplinary team, but for this question we want to know who is finally responsible for the decision.
In this response, the term 'trauma surgeon' refers to an individual who specializes in trauma surgery, not a general surgeon or orthopaedic surgeon who happens to perform damage control surgery as part of wider responsibilities

24. Treatment decisions regarding medical management in Traumatic Brain Injury (TBI) patients in your Intensive Care Unit (ICU) are mainly determined by:

- Trauma surgeon
- Neurosurgeon
- General surgeon or orthopaedic surgeon
- Neurointensivist
- Neurologist
- General Intensivist

Only tick the person(s) that is responsible for the decision. It is possible that options are discussed in a multidisciplinary team, but for this question we want to know who is finally responsible for the decision.
In this response, the term 'trauma surgeon' refers to an individual who specializes in trauma surgery, not a general surgeon or orthopaedic surgeon who happens to perform damage control surgery as part of wider responsibilities

25. Which disciplines are present during daily bedside rounds at your Intensive Care Unit (ICU) in patients with Traumatic Brain Injury (TBI)?

We would like to know which disciplines are present during daily bedside rounds at your ICU. It is possible that some disciplines are only present at indication or at some days in the week. That is why you can select how often the disciplines are present. It is not necessary that different disciplines visit the patients all at the same time, visits might also be sequentially over time.
If there are other disciplines present, please note this.

	Never (0-10%)	Rarely (10-30%)	Sometimes (30-70%)	Frequently (70-90%)	Always (90-100%)
Intensivist	☐	☐	☐	☐	☐
Intensivist trainee in residency training	☐	☐	☐	☐	☐
Neurointensivist	☐	☐	☐	☐	☐
Neurointensivist trainee in residency training	☐	☐	☐	☐	☐
Neurosurgeon	☐	☐	☐	☐	☐
Neurosurgery trainee in residency training	☐	☐	☐	☐	☐
Neurologist	☐	☐	☐	☐	☐
Neurology trainee in residency training	☐	☐	☐	☐	☐
Anesthesiologist	☐	☐	☐	☐	☐
Pharmacist	☐	☐	☐	☐	☐
Neuroradiologist	☐	☐	☐	☐	☐
Nurse	☐	☐	☐	☐	☐
Physician assistant / nurse practitioner	☐	☐	☐	☐	☐
Rehabilitation physician	☐	☐	☐	☐	☐
Physical therapist	☐	☐	☐	☐	☐
Occupational therapist	☐	☐	☐	☐	☐
Medical students	☐	☐	☐	☐	☐
Other, please specify………………….	☐	☐	☐	☐	☐

26. Who is formally in charge during daily rounds for Traumatic Brain Injury (TBI) patients?

- Intensivist
- (Neuro)-intensivist
- Neurosurgeon
- Neurologist
- Anesthesiologist
- Pharmacist
- Other , please specify……………………………………………………….

27. Do you have an electronic data system in your Intensive Care Unit (ICU)?

- No
- Yes

Provider Profiling Questionnaire

Questionnaire 7: treatment at the Intensive Care Unit (ICU)

This questionnaire can be completed by a(n) (neuro)intensivist in collaboration with a neurosurgeon

For the completion of this questionnaire, we advise you to ask help from a data manager, administrative staff member and/or someone from the financial department in your hospital, since we ask for hospital data in this questionnaire. It is very important that this information is accurate, and searched for in annual reports, registries and other data sources rather than estimated.

This questionnaire also includes questions about the general policy in your hospital. The responses to these questions should represent, as best as practicable, a general consensus on treatment at your centre, rather than individual management preferences. Consequently, you should provide responses that describe not what you would do personally, but how the majority of patients would generally be treated in your centre.

There are no 'right' or 'wrong' answers so please give us a realistic and honest view of how the care in your hospital is organized. Your answers will only be used to answer the scientific questions in CENTER TBI and no information in any form will be reported on individual centre level. Some of the questions may seem similar, but please answer all questions.

If you have any questions or problem, please contact:
Maryse Cnossen, PhD student (m.c.cnossen@erasmusmc.nl)

Information about the completer of the questionnaire

Other than the CENTER-TBI investigator, which of the following individuals was involved in completion of this questionnaire?

Select all that apply

- ☐ Neurologist
- ☐ Neurosurgeon
- ☐ Trauma Surgeon
- ☐ ED physician
- ☐ Administrative staff member / data manager / financial department
- ☐ Other, please specify……………..
- ☐ NA. The questionnaire is solely completed by the CENTER TBI local investigator

The Local investigator is the senior clinician(s) at your hospital involved in supervision of CENTER TBI

General patient statistics

What is the number of patients treated in your Intensive Care Unit (ICU) annually?

1. 2012: ……………………………………..
2. 2013: ……………………………………..

What is the number of Traumatic Brain Injury (TBI) patients treated in your Intensive Care Unit (ICU) annually?

3. 2012: ……………………………………..
4. 2013: ……………………………………..

5.In how many patients with Traumatic Brain Injury (TBI) did you perform ICP monitoring last year (2013)?

……………………………………………..

6.Where did you find this information?

………………………………………………………..

Name the source: for example annual report, registry

Indications for admission to your Intensive Care Unit (ICU)

7. Which Traumatic Brain Injury (TBI) patients are admitted to your Intensive Care Unit (ICU)?

Please provide us the general clinical practice at your centre. This does not have to be the same as stated in the guidelines you use

This question is about indications for ICU admission in patients with TBI.<br><br>Select CONSIDERED NOT IMPORTANT IN DECISION MAKING in factors that are never a reason for ICU admission.<br><br>Select ONLY IN THE PRESENCE OF OTHER RISK FACTORS if the factor is never solely a reason ICU admission, but it might be a reason in combination with one or more other risk factors. For example: a hospital may only admit patients with a GCS ≤ 8 if they have an abnormal head CT as well. Respondents from such a hospital should tick 'only in the presence of other risk factors' after GCS ≤ 8

Select GENERAL POLICY when the criteria are, in general, a reason for ICU admission in your hospital. When you select GENERAL POLICY this must represent a general consensus among colleagues, rather than individual preference.
Where you are in doubt whether this is the appropriate response to the question, we would recommend, for example, either a verbal discussion or an email exchange with colleagues to check consensus.

	Considered not important in decision making	Only in the presence of other risk factors	General Policy
GCS ≤ 8	☐	☐	☐
GCS 9-12 with CT abnormalities	☐	☐	☐
GCS 9-12 without CT abnormalities	☐	☐	☐
GCS 13-15 with CT abnormalities but no large structural lesion	☐	☐	☐
GCS 13-15 with small EDH or ASDH	☐	☐	☐
GCS 13-15 with contusional brain damage	☐	☐	☐
anti-coagulant therapy in patients with GCS 13-15	☐	☐	☐
After intracranial surgery	☐	☐	☐
Mild TBI with concomitant extracranial injuries that in isolation, would not necessitate ICU admission	☐	☐	☐

Use of protocols / guidelines

8. With reference to guidelines for Intensive Care Unit (ICU) management of Traumatic Brain Injury (TBI), does your ICU:

- Not have specific guidelines for management
- Follow the Brain Trauma Foundation Guidelines
- Follow National Guidelines (Please specify: ……………………………………………)
- Have institutional guidelines which are broadly based on BTF and/or National Guidelines
- Have separate guidelines which you have developed independently

(if #4 or #5 above, please upload a copy of your guidelines)

9. If guidelines for Traumatic Brain Injury (TBI) management exist in your Intensive Care Unit (ICU), how are they implemented?
Select all that apply

- ☐ No formal implementation of guidelines
- ☐ Verbal direction from clinical managers/ clinical directors/senior doctors
- ☐ Written protocols and algorithms
- ☐ Training organised by an external organisation
- ☐ Training organised by your own hospital / ICU
- ☐ E-learning
- ☐ flowchart / algorithms / protocols in the patient data management system of your ICU
- ☐ Periodic feedback on adherence to the guideline
- ☐ Structural attention for protocol adherence during clinical rounds
- ☐ Other (please specify) ……………………………………………………………………………….

10. Is there a group or individuals who oversee guideline development and maintenance?

- ☐ Neither
- ☐ Individual
- ☐ Group
  - o Single discipline: ICU/neurosurgery/neurology (please circle correct response)
  - o Multidisciplinary

11. Have there been audits to check guideline adherence in your Intensive Care Unit (ICU)?

- o Not in last five years
- o Once in the last five years
- o Annually
- o Several times per year

An audit is a process in which the hospital/ICU assesses how well guidelines are followed.

12. Please estimate Traumatic Brain Injury (TBI) guideline adherence for:

	Never (0-10%)	Rarely (10-30%)	Sometimes (30-70%)	Frequently (70-90%)	Always (90-100%)
For medical management	☐	☐	☐	☐	☐
For surgery	☐	☐	☐	☐	☐

The responses to this question should represent, as best as practicable, a general consensus, rather than individual thoughts.

13. What are reasons for non-adherence to guidelines in patients with Traumatic Brain Injury (TBI) ?

	Never (0-10%)	Rarely (10-30%)	Sometimes (30-70%)	Frequently (70-90%)	Always (90-100%)
Lack of knowledge among clinicians	☐	☐	☐	☐	☐

Every patient is unique and should be managed by clinical judgment	☐	☐	☐	☐	☐
Inadequate time to consult guidelines for urgent decisions	☐	☐	☐	☐	☐
Guidelines on TBI do not apply due to extracranial trauma or comorbidity	☐	☐	☐	☐	☐
Inadequate resources to apply guidelines (ICU beds, personnel, equipment)	☐	☐	☐	☐	☐
Other, please specify………………	☐	☐	☐	☐	☐

The responses to this question should represent, as best as practicable, a general consensus, rather than individual thoughts.

Monitoring

Routinely repeated CT scans

In this context "routine repeat CT scan" refers to CT scans that are scheduled at the beginning of the ICU admission, and undertaken at predetermined time points regardless of clinical situation.

14. Do you use routinely repetitive CT scanning in patients with Traumatic Brain Injury (TBI)?

- No
- Yes

14b. If yes: What are indications for routinely repetitive CT scanning in TBI patients admitted to the ICU? *Please provide us the general clinical practice at your centre. This does not have to be the same as stated in the guidelines you use*

This question is about indications for routinely repetitive CT scanning in TBI patients.

Select CONSIDERED NOT IMPORTANT IN DECISION MAKING in factors that are never a reason for routinely repetitive CT scanning.

Select ONLY IN THE PRESENCE OF OTHER RISK FACTORS if the factor is never solely a reason for routinely repetitive CT scanning, but it might be a reason in combination with one or more other risk factors. For example: a hospital may schedule routinely repetitive CT scanning in patients with severe TBI on coagulant medication. Respondents from such a hospital should tick 'only in the presence of other risk factors' after GCS ≤ 8 and 'patients on anticoagulants/antiplatelet medication'.

Select GENERAL POLICY when the criteria are, in general, a reason for routinely repetitive CT scanning in your hospital. When you select GENERAL POLICY this must represent a general consensus among colleagues, rather than individual preference.

Where you are in doubt whether this is the appropriate response to the question, we would recommend, for example, either a verbal discussion or an email exchange with colleagues to check consensus.

	Considered not important in decision making	Only in the presence of other risk factors	General Policy
Routinely in all TBI cases on the ICU (e.g. 24 or 48 hours post ICU admission)	☐	☐	☐
In all patients with intracranial lesions	☐	☐	☐
In all patients with admission GCS ≤ 8	☐	☐	☐
Patients on anticoagulants/antiplatelet medication	☐	☐	☐
Following intracranial surgery	☐	☐	☐
A history of substance abuse	☐	☐	☐

14c. If you use routinely repetitive CT scanning in TBI patients. What is the typical time scheme?

Scan 1: …….hours after the first CT scan
Scan 2: …….hours after the first CT scan
Scan 3: …….hours after the first CT scan
Scan 4: …….hours after the first CT scan
Scan 5: …….hours after the first CT scan
Scan 6: …….hours after the first CT scan

ICP monitoring

15. What are indications for ICP monitoring in your hospital?
Please provide us the general clinical practice at your centre. This does not have to be the same as stated in the guidelines you use

This question is about indications for ICP monitoring in your centre.

Select CONSIDERED NOT IMPORTANT IN DECISION MAKING in factors that are never a reason for ICP monitoring.

Select ONLY IN THE PRESENCE OF OTHER RISK FACTORS if the factor is never solely a reason for ICP monitoring, but it might be a reason in combination with one or more other risk factors. For example: a hospital may perform ICP monitoring in patients with a GCS ≤ 8 without CT abnormalities if there are other risk factors present. Respondents from such a hospital should tick 'only in the presence of other risk factors' after GCS ≤ 8.

Select GENERAL POLICY when the criteria are, in general, a reason for ICP monitoring in your hospital. When you select GENERAL POLICY this must represent a general consensus among colleagues, rather than individual preference.

Where you are in doubt whether this is the appropriate response to the question, we would recommend, for example, either a verbal discussion or an email exchange with colleagues to check consensus.

	Considered not important in decision making	Only in the presence of other risk factors	General Policy
GCS ≤ 8 and CT abnormalities	☐	☐	☐
GCS ≤ 8 without CT abnormalities	☐	☐	☐
GCS 9-12 with contusion	☐	☐	☐
Inability to assess a patient with CT abnormalities clinically (e.g. sedation, surgery etc.)	☐	☐	☐
Intraventricular haemorrhage	☐	☐	☐
Other, please specify……………………….	☐	☐	☐

16. What are reasons for NOT monitoring ICP at your Intensive Care Unit (ICU)?

	Never (0-10%)	Rarely (10-30%)	Sometimes (30-70%)	Frequently (70-90%)	Always (90-100%)
Glasgow Coma Scale (GCS) > 8	☐	☐	☐	☐	☐
No radiological signs of raised ICP	☐	☐	☐	☐	☐
Risk of raised ICP considered low	☐	☐	☐	☐	☐

Patient considered unsalvageable	☐	☐	☐	☐	☐
Coagulopathy (non-drug related)	☐	☐	☐	☐	☐
Use of anticoagulants or platelet aggregation inhibitors	☐	☐	☐	☐	☐
No device available	☐	☐	☐	☐	☐
Not local policy to monitor ICP	☐	☐	☐	☐	☐
We adhere to a protocol in which treatment is based on imaging and clinical examination	☐	☐	☐	☐	☐
Too costly	☐	☐	☐	☐	☐
Other, please specify……	☐	☐	☐	☐	☐

The responses to this question should represent, as best as practicable, a general consensus on treatment at your centre, rather than individual management preferences or opinions.

17. Is there structural variation between (neuro)surgeons within your hospital with regard to the decision to place an ICP sensor?

- ○ No
- ○ Yes

Structural variation refers to a situation in which one or more of the neurosurgeons are generally more likely to place an ICP sensor than others.

18. When a patient with polytrauma and minor intracranial pathology (which would not otherwise indicate ICP monitoring) requires <u>extracranial surgery</u> which is not life-saving, in the acute phase after trauma, do you:

Select all that apply

- ☐ Place an ICP monitor and allow surgery to proceed
- ☐ Repeat a CT scan before/after surgery
- ☐ Undertake a sedation hold before and/or after surgery
- ☐ Postpone surgery if at all possible
- ☐ Other, please specify…..

The responses to this question should represent, as best as practicable, a general consensus on treatment at your centre, rather than individual management preferences.

19. In polytrauma patients with a Glasgow Coma Scale (GCS) >8 and small but not severe initial CT abnormalities, who require <u>mechanical ventilation</u> for a number of days because of extracranial injuries, we apply ICP monitoring:

- ○ Never
- ○ Only sometimes
- ○ Often
- ○ Always

The responses to this question should represent, as best as practicable, a general consensus on treatment at your centre, rather than individual management preferences.

20. What kind of ICP sensors are used in your hospital?

- o Parenchymal monitors without optional ventricular drainage
- o Ventricular catheters
- o Both
- o Not applicable since ICP sensors are not used

20b. In case you answered 'both' in the previous question:
If you use parenchymal and ventricular catheters in your hospital, when would you use ventricular/ventricular+ sensor monitors (instead of parenchymal monitoring)?

please rank the top 3 reasons:
1: …………..
2: ………….
3: …………..

- ☐ Routine in our department
- ☐ Not routine, but enlarged ventricles
- ☐ External CSF drainage
- ☐ No parenchymal device available
- ☐ Low cost
- ☐ Other, please specify…………………………………

20c. If you use parenchymal and ventricular catheters in your hospital, when would you use parenchymal monitors (instead of ventricular/ventricular+sensor)?

1: …………..
2: ………….
3: …………..

- ☐ Routine in our department
- ☐ Not routine, but small ventricles
- ☐ Mainly motivated by time of day
- ☐ No OR available for placement ventricular catheter
- ☐ Failed implantation ventricular cathether
- ☐ Other, please specify………………………………..

20d. When deciding to monitor ICP we routinely use additional ventricular CSF drainage:

- o No, never or seldom
- o As second tier therapy to control ICP
- o Only if ventricles are enlarged
- o Yes, always initially

20e. If you use a ventricular drain:

During the use of the ventricular drain, is the drain open or closed during the most of the time?

- ☐ Ventricular drain is open to drain CSF
  - o If this answer option is selected: At what level? ……………………………………………
- ☐ Ventricular drain is closed mostly and opened intermittently
  - o If this answer option is selected: At what pressure is this opened? And for how long?

☐ Other, please specify……………………………….

Intensive Care Unit (ICU) practice around ICP monitoring

	Never (0-10%)	Rarely (10-30%)	Sometimes (30-70%)	Frequently (70-90%)	Always (90-100%)	N/A, we do not have this technique
21. Are prophylactic antibiotics given prior to ICP monitor insertion?	Ventricular catheter:					
	○	○	○	○	○	○
	Parenchymal sensor:					
	○	○	○	○	○	○
22. Are prophylactic antibiotics continued after ICP monitoring insertion?	Ventricular catheter:					
	○	○	○	○	○	○
	Parenchymal sensor					
	○	○	○	○	○	○
23. Is a coagulation panel assessed prior to insertion of an ICP monitoring device?	Ventricular catheter:					
	○	○	○	○	○	○
	Parenchymal sensor					
	○	○	○	○	○	○

24. What is considered a minimum platelet count for insertion of a ventricular catheter in your Intensive Care Unit (ICU)?

- ○ >150K
- ○ >100K
- ○ > 80 K
- ○ >50K
- ○ Variable, depends on surgeon
- ○ No minimum
- ○ Other, please specify ……………………………………

25. What is consider the minimum INR for safe placement of a ventricular catheter in your Intensive Care Unit (ICU)?

- <1.4
- <1.3
- <1.2
- Variable, depending on surgeon
- No minimum
- Other, please specify…………………………………………………………….

26. Who inserts the catheter/probes for ICP monitoring?
Please provide us the general clinical practice at your centre. This does not have to be the same as stated in the guidelines you use

This question is about indications for ICP monitoring in your centre.

Select NEVER if the specialism has never inserted ICP monitors in TBI patients.

Select RARELY / EXCEPTIONAL if the specialism CAN insert ICP monitors and does this during exceptional circumstances (e.g. during the night, crisis, overcrowding).

Select GENERAL POLICY when the specialism usually inserts ICP monitors.

Where you are in doubt whether this is the appropriate response to the question, we would recommend, for example, either a verbal discussion or an email exchange with colleagues to check consensus.

	Never	Rarely / Exceptional	General Policy
Neurosurgeon	☐	☐	☐
Neurosurgical resident	☐	☐	☐
Intensivist	☐	☐	☐
Intensivist resident	☐	☐	☐
Neurointensivist	☐	☐	☐
Non-neurosurgical surgeon	☐	☐	☐
Physician assistant / nurse practitioner	☐	☐	☐
Other, please specify….	☐	☐	☐

27. At what level does your Intensive Care Unit (ICU) zero the ICP catheter?
- Foramen of Monro
- Same level as arterial blood pressure
- Other, please specify………………………………

28. At what level does your Intensive Care Unit (ICU) zero the transducer for arterial blood pressure (for calculation of CPP)?
- Right atrium
- Level of arterial catheter
- Foramen of Monroe
- Other, please specify………………..

29. How is the alignment of the transducer to the chosen reference level checked?
- Not formally checked
- By eye
- Spirit level

- o Laser indication
- o Other (please specify)………………..

29b. If the level is formally assessed, is this done:
- o At admission
- o Once each day
- o Once per nursing shift
- o More frequently

CPP monitoring

30. Please list the target Cerebral Perfusion Pressure utilized at your facility:
Select all that apply
- ☐ > 50 mmHg
- ☐ > 60 mmHg
- ☐ > 70 mmHg
- ☐ Individualized

31. For treating CPP, which types of IV fluids are used to augment intravascular volume?
Select all that apply
- ☐ Crystalloids
- ☐ Colloids – starches
- ☐ Colloids - albumin
- ☐ Other combinations

32. Which vasoactive drugs are used to support CPP in patients with Traumatic Brain Injury (TBI)?
Select all that apply
- ☐ Vasopressors
- ☐ Inotropes
- ☐ Other, please specify………

33. What monitoring devices are used to titrate vasoactive drugs?
Select all that apply
- ☐ MAP targets only
- ☐ Central venous pressure
- ☐ PICO
- ☐ Lidco
- ☐ Oesophageal Doppler monitor
- ☐ Pulmonary artery catheter
- ☐ Others, please specify ……

Advanced monitoring

34. Which of the following additional techniques are utilized at your Intensive Care Unit (ICU) for neuromonitoring?
Select all that apply

	Never (0-10%)	Rarely (10-30%)	Sometimes (30-70%)	Frequently (70-90%)	Always (90-100%)
Cerebral	☐	☐	☐	☐	☐

microdialysis					
Transcranial Doppler	☐	☐	☐	☐	☐
CBF probes	☐	☐	☐	☐	☐
Jugular Venous saturation monitors	☐	☐	☐	☐	☐
Near infrared monitors	☐	☐	☐	☐	☐
Brain tissue oxygenation	☐	☐	☐	☐	☐
Other, please specify…………	☐	☐	☐	☐	☐

Treatment of severe Traumatic Brain Injury (TBI) patients at the Intensive Care Unit (ICU)

35. At your Intensive Care Unit (ICU), the threshold for medical management of elevated ICP is:
- > 15 mmHg
- >20 mmHg
- >25 mmHg
- Other, please specify………………

36. At your Intensive Care Unit (ICU), the threshold for performing a decompressive craniectomy in patients with diffuse injury is:
- > 15 mmHg
- >20 mmHg
- >25 mmHg
- > 30 mm Hg
- NA. Decompressive craniectomy is (almost) never performed in our hospital

Sedation

37. Please rate the utilization of the following sedatives, neuromuscular blockers, and analgesics <u>as first line therapies</u> for ICU management of Traumatic Brain Injury (TBI) patients with increased ICP:

	Never (0-10%)	Rarely (10-30%)	Sometimes (30-70%)	Frequently (70-90%)	Always (90-100%)
Fentanyl	☐	☐	☐	☐	☐
Midazolam	☐	☐	☐	☐	☐
Morphine (or other opioids)	☐	☐	☐	☐	☐
Propofol	☐	☐	☐	☐	☐
Neuromuscular blocking agents	☐	☐	☐	☐	☐
Alfa 2 agonist (clonidine or dexmedetomidine)	☐	☐	☐	☐	☐
Barbiturates	☐	☐	☐	☐	☐

Other, please specify…..	☐	☐	☐	☐	☐

Hyperosmolar therapy (mannitol and/or hypertonic saline

Please provide us the general clinical practice at your centre. This does not have to be the same as stated in the guidelines you use

> This question is about the use of hyperosmotic therapy in patients with elevated ICP.
>
> Select NEVER in agents that are never used in your centre.
>
> Select RARELY / EXCEPTIONAL in agents that can be used but are no general policy.
>
> Select GENERAL POLICY when the agent is, in general, used to treat elevated ICP in your centre.
> When you select GENERAL POLICY this must represent a general consensus among colleagues, rather than individual preference.
> Where you are in doubt whether this is the appropriate response to the question, we would recommend, for example, either a verbal discussion or an email exchange with colleagues to check consensus.

	Never	Rarely / Exceptional	General Policy
38. Is mannitol utilized to treat patients with increased ICP in your ICU ?	☐	☐	☐
39. Is hypertonic saline utilized to treat patients with increased ICP in your ICU?	☐	☐	☐
40. Is hypertonic saline administered in conjunction with mannitol?	☐	☐	☐

Mannitol and Hypertonic Saline

	**Mannitol**	**Hypertonic saline**
41. How are these agents administered?	☐ N/A<br>☐ Dose titrated to ICP<br>☐ Fixed Bolus dosing<br>☐ Standard continuous infusion	☐ N/A<br>☐ Dose titrated to ICP<br>☐ Fixed Bolus dosing<br>☐ Standard continuous infusion
42. If administered in fixed bolus doses, how frequently is it given:	☐ N/A<br>☐ More often than 6 times per day<br>☐ 6 times per day (every 4 hours)	☐ N/A<br>☐ More often than 6 times per day<br>☐ 6 times per day (every 4 hours)

	☐ 4 times per day (every 6 hours)<br>☐ Less than 4 times per day<br>☐ Other, please specify……………..	☐ 4 times per day (every 6 hours)<br>☐ Less than 4 times per day<br>☐ Other, please specify…………………….
	If mannitol is administered, is serum osmolarity monitored?<br><br>☐ N/A<br>☐ No<br>☐ Yes<br><br>If yes, an upper limit of ……….. mOsm/liter	If hypertonic saline is administered as a continuous infusion, is there a serum sodium goal?<br>☐ N/A<br>☐ No<br>☐ Yes<br><br>If yes, a goal of …………………(mEq/L)

The responses to this question should represent, as best as practicable, a general consensus on treatment at your centre, rather than individual management preferences.

Second/third tier therapies for treatment of raised intracranial pressure

The responses to the following questions should represent, as best as practicable, a general consensus on treatment at your centre, rather than individual management preferences.

43. Are the following approaches used to treat <u>refractory</u> intracranial hypertension?

	Never (0-10%)	Rarely (10-30%)	Sometimes (30-70%)	Frequently (70-90%)	Always (90-100%)
Decompressive craniectomy	☐	☐	☐	☐	☐
Hypothermia (temperature < 36 degrees Celsius)	☐	☐	☐	☐	☐
Intensive hyperventilation (PCO2 < 4.0 kPa)	☐	☐	☐	☐	☐
Barbiturates	☐	☐	☐	☐	☐

43b. If hyperventilation is answered with rarely – always:
When is hyperventilation (PaCO2 < 4,0 kpa)) utilized in Traumatic Brain Injury (TBI) patients at the intensive care unit?

	Never (0-10%)	Rarely (10-30%)	Sometimes (30-70%)	Frequently (70-90%)	Always (90-100%)
prophylactic hyperventilation (PaCO2 < 35 mmHg)	☐	☐	☐	☐	☐
To manage intracranial pressure for less than six hours	☐	☐	☐	☐	☐
To manage intracranial pressure for more than six hours	☐	☐	☐	☐	☐
In cases of imminent herniation	☐	☐	☐	☐	☐
Is brain tissue oxygen monitoring (PbtO2) used to measure cerebral oxygenation during hyperventilation?	☐	☐	☐	☐	☐

43c. If hyperventilation is used, what is the target PaCO2 (as second/third tier therapy)?

- <35 mmHg
- <30 mmHg
- <25 mmHg

- Variable, dependent on patient
- Variable, dependent on surgeon/intensivist

43d. If hypothermia is used, what is the target temperature?
- >35°
- 35°
- 33 or 34°
- 32°
- Variable depending on patient
- Variable depending on physician

43e. If barbiturates are used, how is the dose targeted?
Select all that apply
- ☐ Continuous EEG monitoring
- ☐ Intermittent EEG recording
- ☐ Serum levels
- ☐ EEG and serum levels
- ☐ ICP control
- ☐ ICP control and EEG

Seizure prophylaxis and management

The responses to the following questions should represent, as best as practicable, a general consensus on treatment at your centre, rather than individual management preferences. These questions refer to the use of drugs to prevent seizures, rather than treat documented seizures

44. What are indications for anti-seizure prophylaxis in your centre?

	Never (0-10%)	Rarely (10-30%)	Sometimes (30-70%)	Frequently (70-90%)	Always (90-100%)
Glasgow Coma Scale (GCS) < 10	☐	☐	☐	☐	☐
Cortical contusion	☐	☐	☐	☐	☐
Depressed skull fracture	☐	☐	☐	☐	☐
Subdural hematoma	☐	☐	☐	☐	☐
Epidural hematoma	☐	☐	☐	☐	☐
Intracerebral hematoma	☐	☐	☐	☐	☐
Penetrating brain injury	☐	☐	☐	☐	☐
Other – please specify ……………………………….. …………………………………	☐	☐	☐	☐	☐

45. What is the duration of recommended anti-seizure <u>prophylaxis</u> at your hospital?
Select all that apply
- ☐ 1-3 days

- ☐ 4-7 days
- ☐ >7 days
- ☐ 3 weeks
- ☐ 3 months
- ☐ Variable depending on patient
- ☐ Variable depending on physician

46. Please rate the utilization of following agents used for seizure prophylaxis of Traumatic Brain Injury (TBI) patients:

	Always (90-100%)	Frequently (90-70%)	Sometimes (30-70%)	Rarely (10-30%)	Never (0-10%)
Phenytoin	☐	☐	☐	☐	☐
Levetiracetam	☐	☐	☐	☐	☐
Valproate	☐	☐	☐	☐	☐
Other, please specify………………..	☐	☐	☐	☐	☐

Treatment of seizures

The responses to the following questions should represent, as best as practicable, a general consensus on treatment at your centre, rather than individual management preferences.

47. Does your Intensive Care Unit (ICU) initiate anti-epileptic treatment after:

	Never (0-10%)	Rarely (10-30%)	Sometimes (30-70%)	Frequently (70-90%)	Always (90-100%)
A single seizure	☐	☐	☐	☐	☐
Two or more seizures	☐	☐	☐	☐	☐

48. Please rate your utilization of following agents used for seizure treatment of Traumatic Brain Injury (TBI) patients:

	Never (0-10%)	Rarely (10-30%)	Sometimes (30-70%)	Frequently (70-90%)	Always (90-100%)
Phenytoin	☐	☐	☐	☐	☐
Levetiracetam	☐	☐	☐	☐	☐
Valproate	☐	☐	☐	☐	☐
Other, please specify………………..	☐	☐	☐	☐	☐

Fever

The responses to the following questions should represent, as best as practicable, a general consensus on treatment at your centre, rather than individual management preferences.

49. Is fever (core temperature >38) in Traumatic Brain Injury (TBI) patients routinely treated in your unit?

- Never (0-10%)
- Rarely (10-30%)
- Sometimes (30-70%)
- Frequently (70-90%)
- Always (90-100%)

50. Please rate your utilization of the following approaches for management of fever in Traumatic Brain Injury (TBI) patients with increased ICP:

	Never (0-10%)	Rarely (10-30%)	Sometimes (30-70%)	Frequently (70-90%)	Always (90-100%)
Paracetamol	☐	☐	☐	☐	☐
NSAIDs	☐	☐	☐	☐	☐
External cooling (cold blankets, etc)	☐	☐	☐	☐	☐
Intravascular cooling	☐	☐	☐	☐	☐

	Never (0-10%)	Rarely (10-30%)	Sometimes (30-70%)	Frequently (70-90%)	Always (90-100%)
51. Are corticosteroids used for the primary management of TBI in patients with head injury?	☐	☐	☐	☐	☐
52. Are corticosteroids used for ICU management of other conditions in TBI patients (e.g.: vasopressor resistant hypotension)? Specify: ………………………………	☐	☐	☐	☐	☐

Deep venous thrombosis (DVT) prophylaxis

The responses to the following questions should represent, as best as practicable, a general consensus on treatment at your centre, rather than individual management preferences.

	Never (0-10%)	Rarely (10-30%)	Sometimes (30-70%)	Frequently (70-90%)	Always (90-100%)
53. How often is	☐	☐	☐	☐	☐

DVT prophylaxis used?

54. If you use DVT prophylaxis, when is DVT prophylaxis initiated?

	< 24 hrs	24-72 hrs	< 72 hrs	Never
In the absence of hemorrhagic lesions	☐	☐	☐	☐
In the presence of hemorrhagic lesion	☐	☐	☐	☐
After intracranial surgery	☐	☐	☐	☐

55. In patients who receive DVT prophylaxis, what medication is given?
- ☐ Subcutaneous unfractioned heparin
- ☐ Low-molecular weight heparin
- ☐ Other, please specify………………………………………………………………………

56. Coagulopathy related to the trauma is treated with :

	Never (0-10%)	Rarely (10-30%)	Sometimes (30-70%)	Frequently (70-90%)	Always (90-100%)
Fresh Frozen plasma (FFP)	☐	☐	☐	☐	☐
Platelets	☐	☐	☐	☐	☐
Fibrinogen	☐	☐	☐	☐	☐
Novo 7 (recombinant factor VII)	☐	☐	☐	☐	☐
Vitamin K	☐	☐	☐	☐	☐
PCC (Prothrombin Complex Concentrate)	☐	☐	☐	☐	☐
Other, please specify…	☐	☐	☐	☐	☐

Respiration and ventilation

The responses to the following questions should represent, as best as practicable, a general consensus on treatment at your centre, rather than individual management preferences.

Mechanical ventilation

57. Select initial PaO2 goal in mechanically ventilated Traumatic Brain Injury (TBI) patients:
- ○ > 8 kpa (60 mmHg)
- ○ > 10 kpha (75mmHg)

- >13 kpa (100 mmHg)
- Other, please specify…………………..

58. Select initial arterial oxygen saturation goal in mechanically ventilated Traumatic Brain Injury (TBI) patients:
- > 85%
- > 90 %
- > 95%
- Other, please specify………………………….

59. In patients remaining unconscious after Traumatic Brain Injury (TBI) , when would your ICU consider a tracheostomy?
- Within 1 week
- 1- 2 weeks
- > 2weeks

60. Select PaCO2 goal for management in mechanically ventilated Traumatic Brain Injury (TBI) patients:

	25-29 mmHg	30-35 mmHg	36-40 mmHg	41-45 mmHg
In the absence of (suspicion of) raised ICP	o	o	o	o
In the presence of raised ICP	o	o	o	o

General ICU treatments / protocols

Red blood cell policy

61. Does the Intensive Care Unit (ICU) protocol specify a target goal for hemoglobin concentration?
- No
- Yes, please specify …………………………………………..

62. Do you have a transfusion target in patients with Traumatic Brain Injury (TBI) in the acute phase?
- >100 g/l or 6 mmol/l
- Between 90g/l or 5.5 mmol/l and 100 g/l or 6 mmol/l
- between 80 g/l or 5 mmol/l and 90 g/l or 5.5 mmol/l
- Between 70 g/l or 4.0 mmol/l and 80 g/l or 5 mmol/l

63. What is your transfusion target in patients with non-neurological critical illness?
- >100 g/l or 6 mmol/l
- between 90g/l or 5.5 mmol/l and 100 g/l or 6 mmol/l
- between 80 g/l or 5 mmol/l and 90 g/l or 5.5 mmol/l
- Between 70 g/l or 4.0 mmol/l and 80 g/l or 5 mmol/l

Glucose

64. Is there a standard protocol for glucose management in Traumatic Brain Injury (TBI) in your Intensive Care Unit (ICU) ?
- No
- Yes

65. What therapy is used in glucose management at your Intensive Care Unit (ICU) ?
- No specific therapy

- ☐ Prophylactic insulin administration (buffered infusion)
- ☐ Insulin administration to correct hyperglycemias
- ☐ Tight glycemic control

Caloric intake / nutrition

66. How is nutrition managed at your Intensive Care Unit (ICU) ?
    - o Always parenteral route
    - o Always Enteral route
    - o Mostly parenteral route, enteral route on indication
    - o Mostly enteral route, parenteral route on indication
    - o Other……………………………………….

67. What caloric intake do you aim for in patients with Traumatic Brain Injury (TBI) at your Intensive Care Unit (ICU) ?
……………………… Kcal/kg/day
    - ☐ Unknown / no protocol

68. When do you usually start parenteral nutrition at your Intensive Care Unit (ICU) in Traumatic Brain Injury (TBI) patients?
    - o As soon as possible (directly after ICU admission)
    - o Within 24 hours post-injury
    - o Within 72 hours post-injury
    - o Within 7 days post-injury
    - o We do not have rules / guidelines for this

69. When do you usually aim for full caloric intake replacement?
    - o At 7 days post-injury
    - o < 7 days post-injury
    - o > 7 days post-injury

Outcome

70. Does your hospital routinely assess the outcome at follow-up according to the Glasgow Outcome Scale (extended)?
    - o No
    - o Yes
    - o Unknown

70b. If yes: Is a structural interview used to assess the Glasgow Outcome Scale (extended)?
- o No
- o Yes
- o Unknown

70c. If yes: Who usually assesses the GOS(E)?
Select all that apply
- ☐ Research nurse
- ☐ Nurse
- ☐ Clinician
- ☐ Other, please specify….

71. Which of the following reasons for disability does your hospital include or exclude in your assessment of the GOS(E)?

In the responses below, you can choose either “include” or “Exclude” as a response to each item. If you tick “Include” means that you assess all of the disability as part of categorizing the patient on the GOSE. If you tick “Exclude” this means that where the disability is thought to be the consequence of an injury other than TBI, it will not be included in the assessment, and you would assign the GOS(E) as if that disability did not exist.

	Include	Exclude
Effects of health conditions that existed before the injury, such as cognitive impairment or physical limitations.	☐	☐
Effects of injuries sustained on the same occasion to parts of the body other than the head, such as paralysis due to spinal cord injury or injuries to the limbs.	☐	☐
Effects of external damage to the head or injury to the skull, such as limitations in activities due to a missing bone flap.	☐	☐
Effects of illness arising after TBI treatment, such as pulmonary complications after ventilation.	☐	☐
Effects of a subsequent illness unconnected to TBI, such as pneumonia after flu.	☐	☐
Effects of a subsequent operation, such as hip replacement, that is unconnected to TBI.	☐	☐
Effects of changed social circumstances, such as lower income after injury.	☐	☐
Effects of depression that has arisen since the TBI.	☐	☐
Effects of post-traumatic stress disorder that has appeared since the TBI.	☐	☐
Effects of post-injury anxiety states, such as the development of agoraphobia.	☐	☐

Delirium

72. With regards to the assessment of delirium, does your ICU undertake:

- o No assessment of delirium
- o Assessment of delirium based on general clinical assessment
- o Assessment of delirium using an ICU scale for delirium, such as the CAM-ICU, ICDSC, DDS, Nu-DESC

The DSM criteria for delirium include a disturbance of consciousness with impaired attention; a change in cognitive function (eg, memory impairment, disorientation, or language disturbance) or a perceptual disturbance; the disturbance develops over a short period of time (hours to days) and fluctuates; and the history, physical examination, or laboratory data suggest that the abnormalities are caused by a general medical condition and are not better accounted for by a preexisting dementia. Delirious patients are classified into two behavioral subtypes: ‘‘hyperactive delirium,’’ in which patients are agitated, loud, combative, and likely to inflict significant harm on themselves or others, and ‘‘hypoactive delirium,’’ in which they are withdrawn and have minimal interaction with health providers or family.

Provider Profiling Questionnaire

Questionnaire 8: Ethical aspects of the ICU

This questionnaire can be completed by a(n) (neuro)intensivist in collaboration with a neurosurgeon / trauma surgeon

This questionnaire includes questions about the general policy in your hospital. The responses to these questions should represent, as best as practicable, a general consensus on treatment at your centre, rather than individual management preferences. Consequently, you should provide responses that describe not what you would do personally, but how the majority of patients would generally be treated in your centre.

There are no 'right' or 'wrong' answers so please give us a realistic and honest view of how the care in your hospital is organized. Your answers will only be used to answer the scientific questions in CENTER TBI and no information in any form will be reported on individual centre level. Some of the questions may seem similar, but please answer all questions.

If you have any questions or problem, please contact:
Maryse Cnossen, PhD student (m.c.cnossen@erasmusmc.nl)

Information about the completer of the questionnaire

Other than the CENTER-TBI investigator, which of the following individuals was involved in completion of this questionnaire?
Select all that apply

- ☐ Neurologist
- ☐ Neurosurgeon
- ☐ Trauma Surgeon
- ☐ Emergency Department (ED) physician
- ☐ Administrative staff member / data manager / financial department
- ☐ Other, please specify…………….
- ☐ NA. The questionnaire was completed solely by the CENTER TBI local investigator

The Local investigator is the senior clinician(s) at your hospital involved in supervision of CENTER TBI

Withdrawal of life support

1.Of all patients with severe neurological damage who die on the ICU, approximately, how many die after withdrawal of life-sustaining measures?

- o 0-25%
- o 26-50%
- o 51-75%
- o >75%

2. How is the decision reached to withhold/withdraw life-sustaining measures (e.g. mechanical ventilation, vasoactive medication, renal replacement therapy, intravenous fluid administration)?
Select all that apply

- ☐ One physician (e.g. the most senior person) decides following multidisciplinary discussion
- ☐ During multidisciplinary discussion in which the majority (more than 50%) has to agree
- ☐ During multidisciplinary discussion in which there has to be unanimous consensus among all participating doctors
- ☐ One physician decides (along with objective medical criteria) without multidisciplinary discussion (veto)
- ☐ Other, please specify …………………………….

3. How is a decision reached to not treat patients surgically, because the primary brain damage is considered too devastating (poor prognosis)?
Select all that apply

- ☐ One physician (e.g. the most senior person) decides following multidisciplinary discussion
- ☐ During multidisciplinary discussion in which the majority (more than 50%) has to agree
- ☐ During multidisciplinary discussion in which there has to be unanimous consensus among all participating doctors
- ☐ One physician decides (along with objective medical criteria) without multidisciplinary discussion (veto)
- ☐ Other, please specify …………………………….

4. To what extent do opinions of legal representatives/relatives play a role in decision-making about withdrawal/withholding of life-sustaining measures?

- o None, treatment decisions are always made by the medical staff
- o Treatment decisions are always made by medical staff but legal representatives/relatives can ask for a second opinion

- To some extent
- To a great extent, their opinions are leading in the decision making

5. If the decision is made to withdraw life-sustaining measures, do you preemptively administer sedatives and opioids?
    - No
    - Yes
    - Sometimes
    - Unknown

6. If the decision is made to withdraw life-sustaining measures and before actual withdrawal, do you initiate palliative therapy in anticipation of distressing symptoms (such as pain, terminal restlessness, death rattle, stridor, dyspnoea)?

- No, we withdraw all treatment, including administration of sedatives and/or opioids, and treat symptoms only if they occur
- Yes, we withdraw all treatment, but we provide anticipatory treatment of distressing symptoms only with continuation of sedatives and/or opioids in doses which were given before withdrawal
- Yes, we withdraw all treatment, but we provide anticipatory therapy only with continuation and subsequent increase of sedatives and/or opioids beyond (=dosed higher than strictly needed for) comfort
- Yes, we withdraw all treatment, but we provide anticipatory treatment with continuation of sedatives and opioids and give anticipated medication for stridor and death rattle and other distressing symptoms
- Yes, we withdraw all treatment, but we provide anticipatory treatment with continuation of sedatives and opioids and subsequent increase of sedatives and/or opioids beyond (=dosed higher than strictly needed for) comfort and give anticipated medication for stridor and death rattle and other distressing symptoms
- Other, please specify ………………………..

6b. If yes: is this formalized in a protocol?
- No
- Yes

7. If the decision is made to withdraw life-sustaining measures, do you remove the endotracheal tube?
- No, never
- Yes, always
- Sometimes
- Only on request of the relatives

8. If the decision is made to withdraw life-sustaining measures in a comatose severely injured TBI patent, which life-sustaining measures do you stop?
Select all that apply
- ☐ We stop mechanical ventilation
- ☐ We stop administration of vasoactive medication
- ☐ We stop renal replacement therapy
- ☐ We stop administration of intravenous fluids

- We stop nasogastric feeding

9. If the decision is made to withdraw life-sustaining measures, in a patient with high intracranial pressure, but who is not brain dead, would you remove the ventricular drain (for CSF drainage), but continue other life-sustaining measures in the hope that the patient will become brain dead and thereby becomes a suitable candidate for organ donation?
  - No, never
  - Yes, sometimes
  - Yes, always

10. If the patient has a GCS of 3 and non-reactive pupils and he/she is NOT a candidate for organ donation, do you:

Test brainstem reflexes (oculocephalic reflexes, oculovestibular reflexes, corneal reflexes, cough reflex) before withdrawal of mechanical ventilation?
  - No
  - Yes

When answered 'yes' do you withdraw ventilation only if the brainstem reflexes are negative?
  - No
  - Yes

Order ancillary tests, as electroencephalogram, transcranial Doppler, CT-angiography?
  - No
  - Yes

When answered 'yes' do you withdraw ventilation only if the ancillary tests are negative?
  - No
  - Yes

Test apnea?
  - No
  - Yes

11. When do you declare a patient brain dead?
  - With GCS 3, fixed dilated pupils and no confounding factors [e.g. hypothermia, barbiturates]
  - With GCS 3 and absent brain stem reflexes and no confounding factors
  - With GCS 3, absent brain stem reflexes and apnea and no confounding factors
  - With GCS 3, absent brain stem reflexes, apnea and ancillary test(s) (eg EEG or cerebral angiography) and absence of confounding factors
  - Other, please specify …………………………………….

12. Must the patient, who is not suitable for organ donation, be declared brain dead before withdrawal of life-sustaining measures?
Select all that apply
  - No, the prospect of a very poor prognosis can be enough
  - No, GCS 3 and fixed dilated pupils and no confounders is enough to stop treatment
  - Yes, this is mandatory by law in my country
  - Yes, it is not mandatory by law, but I always do that to be sure

13. Would you consider organ donation after circulatory arrest in a patient in whom mechanical ventilation will be withdrawn, but who is not brain dead?
  - No, this is forbidden in my country

- No, although it would be permitted, I would not do this
- Yes, sometimes
- Yes, always

14. After withdrawal of mechanical ventilation and after circulatory arrest, when exactly do you declare the patient dead in case of a circulatory death organ donor?

- directly after circulatory arrest determined after a ‘flatliner-ECG’ on the monitor
- after one minute ‘flatliner-ECG’ indicating circulatory arrest
- after two minutes ‘flatliner-ECG’
- after five minutes ‘flatliner-ECG’
- after ten minutes ‘flatliner-ECG’
- after loss of pulsatile arterial curve on the invasive arterial blood pressure tracing
- Other, please specify …………………………………….

15. After withdrawal of mechanical ventilation and after circulatory arrest, after how many minutes circulatory arrest do you declare the patient dead in cases not suitable as organ donor ?

- directly after circulatory arrest determined after a ‘flatliner-ECG’ on the monitor
- after one minute ‘flatliner-ECG’
- after two minutes ‘flatliner-ECG’
- after five minutes ‘flatliner-ECG’
- after ten minutes ‘flatliner-ECG’
- after loss of pulsatile arterial curve on the invasive arterial blood pressure tracing
- Other, please specify ……………………………….

16. At what time after injury would you consider to withdraw life support in a patient with TBI, who is in a very poor prognostic condition (based on CT scan, GCS, clinic, ICP etc), but not brain dead?

	Never	Sometimes	Often	Always
24 hours	☐	☐	☐	☐
2-4 days	☐	☐	☐	☐
4-7 days	☐	☐	☐	☐
>1week	☐	☐	☐	☐
>2weeks	☐	☐	☐	☐

17. Does the age of the patient influence your decision making about withholding and withdrawing life-sustaining treatment?

- Yes, always, independent of other criteria
- Yes, but only in combination with other criteria as CT scan, GCS, depth of coma
- No, I only decide on the severity of the injury and anticipated prognosis

Age and ICU admission

18. Do you admit very elderly (80 years and older) patients with severe TBI on the ICU for treatment?

- No, never
- Yes, if the patient is intubated and ventilated in the ER setting
- Yes, if the patient needs ICU treatment with the prospect of saving his/her life
- Yes, but highly depending on the severity of co-morbidity
- Yes, but only if the relatives ask me

19. What are possible reasons for admitting a very elderly patient with severe TBI to the ICU?

Select all that apply

- ☐ Age is not a criterion for triage
- ☐ Elderly patients can benefit from ICU admission
- ☐ Prognosis is unsure
- ☐ Good pre-injury quality of life
- ☐ Absence of pre-injury level of frailty and disability
- ☐ Absence of pre-injury co-morbidity
- ☐ Everybody, independent of age, deserves a chance

20. What are possible reasons for *not* admitting a very elderly patient with severe TBI to the ICU?

Select all that apply

- ☐ The patient is 80+
- ☐ Patients or family do not want any escalation of care
- ☐ Anticipated prognosis
- ☐ These patients are more prone to severe iatrogenic complications from invasive measures and monitoring
- ☐ Impaired pre-injury quality of life
- ☐ Pre-injury level of frailty and disability
- ☐ Pre-injury co-morbidity
- ☐ Scarcity of ICU beds

Provider Profiling Questionnaire

Questionnaire 9: Neurosurgery

This questionnaire can be completed by a neurosurgeon, trauma surgeon or other surgeon who performs surgery in TBI patients

For the completion of this questionnaire, we advise you to ask help from a data manager, administrative staff member and/or someone from the financial department in your hospital, since we ask for hospital data in this questionnaire. It is very important that this information is accurate, and searched for in annual reports, registries and other data sources rather than estimated.

This questionnaire also includes questions about the general policy in your hospital. The responses to these questions should represent, as best as practicable, a general consensus on treatment at your centre, rather than individual management preferences. Consequently, you should provide responses that describe not what you would do personally, but how the majority of patients would generally be treated in your centre.

There are no 'right' or 'wrong' answers so please give us a realistic and honest view of how the care in your hospital is organized. Your answers will only be used to answer the scientific questions in CENTER TBI and no information in any form will be reported on individual centre level. Some of the questions may seem similar, but please answer all questions.

If you have any questions or problem, please contact:
Maryse Cnossen, PhD student (m.c.cnossen@erasmusmc.nl)

Information about the completer of the questionnaire

Other than the CENTER-TBI investigator, which of the following individuals was involved in completion of this questionnaire?
Select all that apply

- ☐ Neurologist
- ☐ Neurosurgeon
- ☐ Trauma Surgeon
- ☐ Emergency Department (ED) physician
- ☐ Administrative staff member / data manager / financial department
- ☐ Other, please specify……………..
- ☐ NA. The questionnaire was completed solely by the CENTER TBI local investigator

The Local investigator is the senior clinician(s) at your hospital involved in supervision of CENTER TBI

Volume

1.In the year 2013, how many Traumatic Brain Injury (TBI) related surgeries have been performed with the following diagnoses?
Please find the exact number of surgeries performed in your annual report / registry

- Acute subdural hematoma n = ………
- Contusion/traumatic intracerebral hematoma n = ………
- Decompressive craniectomy for TBI – hemicraniectomy n = ………
- Decompressive craniectomy for TBI – bifrontal n = ………
- Decompressive craniectomy for TBI – removal previous bone flap n = ………
- Depressed skull fracture n = ………
- Epidural hematoma n = ………
- Ventriculostomy in TBI patients n = ………
- Cranioplasty n = ………

1b. Where did you find this information?
……………………………………………………..

Name the source: for example annual report, registry

Staffing

2. How many neurosurgeons (in FTE) work at your hospital?
……………….FTE neurosurgeons
……………….FTE neurosurgery trainees in residency training
……………….FTE neurosurgery trainees not in residency training

FTE = Full time equivalent. '1 FTE' may be constituted by one person who works on a fulltime basis, but can also refer to two persons who work half-time.<br>The amount of FTEs do not have to be a whole number. If the amount of FTE is, for example, 3.3, please write down '3.3' here and not '3'!<br>If there are persons with out of hours work that is contracted and paid for, you can count them as > 1 FTE. For example, if there is a physician that is paid for 60 hours a week and 48 hours a week is considered as a FTE for a doctor in your hospital, you can count this physician as 60/48 = 1.25 FTE<br><br>The term 'trainee not in residency training' refers to a clinician working in your hospital who is not qualified as a specialist, but is also not part of a formal training scheme towards becoming a specialist (neurosurgeon in this case).

3. How many trauma surgeons (in FTE) work at your hospital?
……………….FTE trauma surgeons

……………….FTE trauma surgeon trainees in residency training
……………….FTE trauma surgeon trainees not in residency training

FTE = Full time equivalent. '1 FTE' may be constituted by one person who works on a fulltime basis, but can also refer to two persons who work half-time.<br><br>The amount of FTEs do not have to be a whole number. If the amount of FTE is, for example, 3.3, please write down '3.3' here and not '3'!<br>If there are persons with out of hours work that is contracted and paid for, you can count them as > 1 FTE. For example, if there is a physician that is paid for 60 hours a week and 48 hours a week is considered as a FTE for a doctor in your hospital, you can count this physician as 60/48 = 1.25 FTE<br><br>The term 'trainee not in residency training' refers to a clinician working in your hospital who is not qualified as a specialist, but is also not part of a formal training scheme towards becoming a specialist (trauma surgeon in this case).<br><br>In this response, the term 'trauma surgeon' refers to an individual who specializes in trauma surgery, not a general surgeon or orthopaedic surgeon who happens to perform damage control surgery as part of wider responsibilities

4. Treatment decisions regarding neurosurgical interventions in Traumatic Brain Injury (TBI) patients in your Intensive Care Unit (ICU) are mainly determined by:
   - o Trauma surgeon
   - o Neurosurgeon
   - o General surgeon or orthopaedic surgeon
   - o Neurointensivist
   - o Neurologist
   - General Intensivists

Only tick the person(s) that is responsible for the decision. It is possible that options are discussed in a multidisciplinary team, but for this question we want to know who is finally responsible for the decision.<br>In this response, the term 'trauma surgeon' refers to an individual who specializes in trauma surgery, not a general surgeon or orthopaedic surgeon who happens to perform damage control surgery as part of wider responsibilities

5. In your hospital, what surgical discipline can be involved in the most urgent cranial surgical interventions in patients with life threatening intracranial space-occupying lesions (sometimes referred to as "super emergency" situations)?
Intracranial surgery does not include ICP monitor placement

	Never (0-10%)	Rarely (10-30%)	Sometimes (30-70%)	Frequently (70-90%)	Always (90-100%)
Neurosurgeon	☐	☐	☐	☐	☐
Trauma surgeon	☐	☐	☐	☐	☐
General / orthopaedic surgeon	☐	☐	☐	☐	☐
Other , please specify………..............	☐	☐	☐	☐	☐

A super-emergency situation refers to a situation in which a patients needs immediate TBI related surgery (knife to skin within 1 hour of arrival in the hospital).<br>In this response, the term 'trauma surgeon' refers to an individual who specializes in trauma surgery, not a general surgeon or orthopaedic surgeon who happens to perform damage control surgery as part of wider responsibilities

6. In your hospital, is there 24/7 qualified neurosurgical coverage?
Select all that apply

- ☐ No
- ☐ There is 24/7 in-house availability of a qualified neurosurgeon
- ☐ There is 24/7 in-house availability of a neurosurgical trainee in residency training
- ☐ Qualified neurosurgeons are on call and will arrive within 30 minutes
- ☐ Neurosurgical trainees in residency training are on call and will arrive within 30 minutes
- ☐ Qualified neurosurgeons are on call and will arrive in more than 30 minutes
- ☐ There is 24/7 access to a qualified neurosurgeon by telecommunication / phone. Qualified neurosurgeons are however not 24/7 in-house or on call
- ☐ Other, please specify …………………

7. What is the general policy with regard to management of extremity (limb) fractures in patients with severe Traumatic Brain Injury (TBI) ?
   - o Damage control: We focus on the TBI. All extremity fractures are stabilized, but definitive treatment delayed
   - o Definitive care: We try to operate/fixate the extremity fractures as soon as possible

The responses to this question should represent, as best as practicable, a general consensus on treatment at your centre, rather than individual management preferences.

8. Do you have guidelines, protocols or policy documents about this topic?
   - o No
   - o Yes, in favour of damage control
   - o Yes, in favour of definitive care

Surgical management of traumatic intracranial mass lesions

Again, the following questions should represent a general consensus on treatment at your centre, rather than individual management preferences.

9. How is the size of a focal post-traumatic mass lesion estimated on CT?
Select all that apply
   - ☐ Based on visual intuition by the neurosurgeon (e.g. no actual measurement)
   - ☐ Based on width, diameter and/or amount of midline shift of the mass lesion
   - ☐ Based on volume measurements with imaging software
   - ☐ Based on volume measurements with direct calculation (e.g. the formula for an ellipsoid)
   - ☐ Other, please specify ………………………

10. Are the Brain Trauma Foundation guidelines followed for:

	Never (0-10%)	Rarely (10-30%)	Sometimes (30-70%)	Frequently (70-90%)	Always (90-100%)
Epidural hematoma (EDH) management?	☐	☐	☐	☐	☐
Acute Subdural hematoma (SDH) management ?	☐	☐	☐	☐	☐

Management of intraparenchymal mass lesions (contusions)?	☐	☐	☐	☐	☐

Surgical management of acute SDH

	Never (0-10%)	Rarely (10-30%)	Sometimes (30-70%)	Frequently (70-90%)	Always (90-100%)
11. Is the decision on surgery in acute SDH influenced by age?	☐	☐	☐	☐	☐

12. Are there acute SDH volume/ thickness thresholds above which your protocol/institutional practice advises surgery (i.e. evacuation)?

- o No
- o Yes

12b. If yes, which minimum volume or thickness is used as threshold to operate?
……………………………………

13. Can decompressive craniectomy be added to the surgical evacuation of the acute SDH?

- o Yes, standard and routinely in every patient
- o Yes, but dependent on intraoperative findings
- o No, sometimes delayed in a second procedure in case of uncontrollable ICP
- o No, never

Surgical management of intraparenchymal mass lesions (contusions)

14. The general policy in your institute for management of intraparenchymal mass lesions (contusions) is:

- ☐ Pre-emptive surgery to prevent deterioration
- ☐ Delayed surgery only after deterioration including intracranial hypertension
- ☐ Variable, depending on surgeon
- ☐ Other, please specify…………………………..

15. Can decompressive craniectomy be added to the surgical evacuation of the intraparenchymal hematoma?

- o Yes, standard and routinely in every patient
- o Yes, but dependent on intraoperative findings
- o No, sometimes delayed in a second procedure in case of uncontrollable ICP
- o No

16. In case of refractory intracranial hypertension / pressure, how often do you use decompressive craniectomy in patients with severe Traumatic Brain Injury (TBI)?

- Never (0-10%)
- Rarely (10-30%
- Sometimes (30-70%)
- Frequently (70-90%)
- Always (90-100%)

If rarely – always is ticked:
16b. When is decompressive craniectomy mostly employed?

- Early – within 6-12 hours of refractory ICP
- Later – as a last effort to control ICP

17. Are there ICP thresholds (eg > 20, 25, 30 mm Hg), above which you would consider delayed decompressive craniectomy?

- No, I would never perform a decompressive craniectomy
- No, I do not use ICP values in the decision to perform a decompressive craniectomy
- Yes

The responses to this question should represent, as best as practicable, a general consensus on treatment at your centre, rather than individual management preferences.

17b. If yes, which threshold?
…………………………………………

17c. If yes, which other patient related parameters do you use to decide upon performing a decompressive craniectomy?
………………………………………..

Please list or state 'None'

17d. If no I do not use ICP values in the decision to perform a decompressive craniectomy:
What patient related parameters do you use to decide upon performing a decompressive craniectomy?
……………………………………

18. What are indications for decompressive craniectomy?
Select all that apply

- ☐ Pre-emptive approach to treatment of (suspected) raised ICP (not last resort)
- ☐ Raised ICP, refractory to medical management (last resort)
- ☐ ICP not monitored, but CT evidence of raised ICP
- ☐ Not directly planned, but decided on because of intra-operative brain swelling
- ☐ Routinely performed with every acute SDH or contusion evacuation

Neurosurgical decision making

19. Is there structural variation between (neuro)surgeons within your hospital with regard to the decision to place an ICP sensor?

- No
- Yes

Structural variation refers to a situation in which one or more of the neurosurgeons are generally more likely to place an ICP sensor than others.

20. Is there structural variation between (neuro)surgeons within your hospital with regard to the decision to evacuate a mass lesion?

- No
- Yes
- Both, depending on what type of mass lesion. Please elaborate…………………………………………………………

Structural variation refers to a situation in which one or more of the neurosurgeons are generally more likely to evacuate a mass lesion than others.

21. What factors influence surgical decision-making in patients with Traumatic Brain Injury (TBI) ?

	Never (0-10%)	Rarely (10-30%)	Sometimes (30-70%)	Frequently (70-90%)	Always (90-100%)
Age of the patient	☐	☐	☐	☐	☐
pupillary size and responses	☐	☐	☐	☐	☐
Time of the day	☐	☐	☐	☐	☐
Time from trauma to ED	☐	☐	☐	☐	☐
Persistent high ICP	☐	☐	☐	☐	☐
Guidelines / protocols	☐	☐	☐	☐	☐
Expertise of the surgeon	☐	☐	☐	☐	☐
Opinion of other disciplines (.e.g intensivists, nurses)	☐	☐	☐	☐	☐
Whether the TBI was a consequence of a suicide attempt	☐	☐	☐	☐	☐
Other, please specify……….	☐	☐	☐	☐	☐

Provider Profiling Questionnaire

Questionnaire 10: Rehabilitation

This questionnaire can be completed by a neurologist, neurosurgeon or trauma surgeon with knowledge about inhospital rehabilitation facilities and referral.

This questionnaire includes questions about the general policy in your hospital. The responses to these questions should represent, as best as practicable, a general consensus on treatment at your centre, rather than individual management preferences. Consequently, you should provide responses that describe not what you would do personally, but how the majority of patients would generally be treated in your centre.

There are no 'right' or 'wrong' answers so please give us a realistic and honest view of how the care in your hospital is organized. Your answers will only be used to answer the scientific questions in CENTER TBI and no information in any form will be reported on individual centre level. Some of the questions may seem similar, but please answer all questions.

If you have any questions or problem, please contact:
Maryse Cnossen, PhD student (m.c.cnossen@erasmusmc.nl)

Information about the completer of the questionnaire

Other than the CENTER-TBI investigator, which of the following individuals was involved in completion of this questionnaire?
Select all that apply

- ☐ Neurologist
- ☐ Neurosurgeon
- ☐ Trauma Surgeon
- ☐ Emergency Department (ED) physician
- ☐ Administrative staff member / data manager / financial department
- ☐ Rehabilitation physician
- ☐ Other, please specify…………….
- ☐ NA. The questionnaire was completed solely by the CENTER TBI local investigator

The Local investigator is the senior clinician(s) at your hospital involved in supervision of CENTER TBI

In-hospital care

1.What rehabilitation facilities are available at your institution
Select all that apply

- ☐ The hospital does not have an inhospital rehabilitation ward to which patients can be admitted for clinical rehabilitation
- ☐ The hospital has a rehabilitation unit where TBI patients can be referred to after Intensive Care Unit (ICU) or ward admission
- ☐ The hospital has structural connections with rehabilitation settings outside the hospital; patients are referred after Intensive Care Unit (ICU) or ward admission
- ☐ The hospital has an outpatient rehabilitation facility
- ☐ Other, please specify………………………..

Please note: When responding to this question, the term "rehabilitation facility" refers to a full multi-disciplinary rehabilitation service, not isolated physiotherapy provision

2. Can you consult rehabilitation specialists (e.g. physical therapists, occupational therapists, dieticians, psychologists, psychiatrists, rehabilitation physicians, speech therapists, social workers, nurses) for patients in your Intensive Care Unit (ICU) or hospital ward?
Select all that apply

	ICU	Hospital ward
No	☐	☐
We can consult rehabilitation specialists on an individual basis	☐	☐
We can consult a multidisciplinary rehabilitation team	☐	☐

2b. if the second and/or third option is ticked: what rehabilitation disciplines are available to treat patients in your Intensive Care Unit (ICU) or acute hospital ward?
Select all that apply

	ICU	Hospital ward

Physical therapist	☐	☐
Occupational therapist	☐	☐
Dietician	☐	☐
(Neuro-) psychologist	☐	☐
Psychiatrist	☐	☐
Rehabilitation physician	☐	☐
Speech therapist	☐	☐
Social worker	☐	☐
Nurse	☐	☐
Other, please specify……………………. …………………………………………………	☐	☐

2c. If a rehabilitation physician is available (if this box is ticked)
When is the rehabilitation physician consulted?

ICU	Hospital ward
○ In every patient	○ In every patient
○ The rehabilitation physician is consulted on indication (not standard)	○ The rehabilitation physician is consulted on indication (not standard)

What is the task of the rehabilitation physician?
Select all that apply

ICU	Hospital ward
☐ Triage (where should the patient be referred to)	☐ Triage (where should the patient be referred to)
☐ Making a treatment plan for initial in-hospital rehabilitation	☐ Making a treatment plan for initial in-hospital rehabilitation
☐ Part of multidisciplinary consultation (determining treatment policy)	☐ Part of multidisciplinary consultation (determining treatment policy)
☐ Not defined	☐ Not defined

3. Are acute medical rehabilitation guidelines or protocols used for patients with Traumatic Brain Injury (TBI) at the Intensive Care Unit (ICU) or the acute hospital ward?
- ○ No. we do not have acute rehabilitation guidelines regarding TBI patients
- ○ Yes, we have acute rehabilitation guidelines for TBI patients

If you do not know the answer to this question yourself, please contact the rehabilitation facility in your hospital

3b. If yes: can you provide us your protocol as pdf / internet link
4. Is coma stimulation (for example pharmacological, neurophysiological or psychological stimulation) used in comatose Traumatic Brain Injury (TBI) patients?
- ○ No
- ○ Yes

4b. If yes: What kind of stimulations are used?
Select all that apply

- ☐ Pharmacologic stimulation
- ☐ Sensory stimulation (Visual, auditory, touch, smell, taste)
- ☐ Mobility stimulation (movement, position)
- ☐ Other, please specify…………………………..

Referral

5. Where are Traumatic Brain Injury (TBI) patients with the following clinical characteristics generally referred to?

> You can select multiple centres here, but only select those that are part of your general policy. For example if you always refer a particular patient group to a rehabilitation centres and some exceptions to a nursing home, only tick rehabilitation centre here.
> If approximately 70% of the patient in the particular category is referred to a rehabilitation centre and the other 30% to an outpatient rehabilitation facility, you can select both.
> The response that you provide should represent, as best as practicable, a general consensus on treatment at your centre, rather than individual thoughts or preferences.

Select all that apply

	Rehabilitation centre	Nursing home	Psychiatric hospital	Outpatient rehab facilities	General practitioner / Health Centre	Local / Regional Hospital	Coma care	Other, please specify
Young patient, not obeying commands	☐	☐	☐	☐	☐	☐	☐	☐
Elderly patient (> 65) not obeying commands	☐	☐	☐	☐	☐	☐	☐	☐
Young patients obeying commands but still in PTA and with severe behavioral problems	☐	☐	☐	☐	☐	☐	☐	☐
Elderly patients (>65) obeying commands but still in PTA and with severe behavioral problems	☐	☐	☐	☐	☐	☐	☐	☐
Non-native language speaking patients	☐	☐	☐	☐	☐	☐	☐	☐

6. Where are Traumatic Brain Injury (TBI) patients without a health care insurance <u>generally</u> referred to?
Select all that apply.

- ☐ N/A in our country (everyone has a health care insurance)
- ☐ Not important in the referral decision
- ☐ Rehabilitation centre

- ☐ Nursing home
- ☐ Psychiatric hospital
- ☐ Outpatient rehabilitation facilities
- ☐ General practitioner / Health centre
- ☐ Local / Regional Hospital
- ☐ Coma Care
- ☐ Other, please specify…………………………………….

7. Do you have the possibility to refer unconscious or minimally responsive patients to settings with coma stimulation programs?

- o No
- o Yes

8. Does patients' age have a major influence on referral decisions?

- o No
- o Yes

If yes: How?

………..

The responses to this question should represent, as best as practicable, a general consensus on treatment at your centre, rather than individual management preferences.

9. Approximately, what is the average waiting time for realization of discharge to referral institutes?

With waiting time we mean the time between the moment that the patient is ready to be discharged from the hospital and the time he/she is admitted or first visits the referral institutes. The waiting time probably varies per patient, geographic location and also varies over time. Please give us an estimate over the last year here.

	Rehabilitation centre	Nursing home	Psychiatric hospital	Outpatient rehab facilities	General practitioner / Health Centre	Local / Regional Hospital	Coma care	Other institution were we refer TBI rehabilitation patients to, please specify …..
Within a few days	☐	☐	☐	☐	☐	☐	☐	☐
Within one week	☐	☐	☐	☐	☐	☐	☐	☐
Within one month	☐	☐	☐	☐	☐	☐	☐	☐
> one month	☐	☐	☐	☐	☐	☐	☐	☐
> three months	☐	☐	☐	☐	☐	☐	☐	☐
> six months	☐	☐	☐	☐	☐	☐	☐	☐

10. Are any of the following factors important for the acceptance policy of rehabilitation institutes?

	No	Yes	If yes, how does it influence rehabilitation policy *Select all that apply*
Non-native language speaking patient	☐	☐	☐ Patients are less often referred to a rehabilitation centre<br>☐ Patients are more often referred to a nursing home

			☐ Patients are more often referred home / to the GP<br>☐ Follow-up appointments in the hospital are less often scheduled<br>☐ Other, please specify………………………………………………
Uninsured patient	☐	☐	☐ Patients are less often referred to a rehabilitation centre<br>☐ Patients are more often referred to a nursing home<br>☐ Patients are more often referred home / to the GP<br>☐ Follow-up appointments in the hospital are less often scheduled<br>☐ Other, please specify………………………………………………
Illegal foreigner	☐	☐	☐ Patients are less often referred to a rehabilitation centre<br>☐ Patients are more often referred to a nursing home<br>☐ Patients are more often referred home / to the GP<br>☐ Follow-up appointments in the hospital are less often scheduled<br>☐ Other, please specify………………………………………………
Legal foreigner	☐	☐	☐ Patients are more often referred to a nursing home<br>☐ Patients are more often referred home / to the GP<br>☐ Follow-up appointments in the hospital are less often scheduled<br>☐ Other, please specify………………………………………………

The responses to this question should represent, as best as practicable, a general consensus on treatment at your centre, rather than individual thoughts.

11. Information communication between acute care providers and rehabilitation facilities is generally by:
Select all that apply

- ☐ Personal interaction (telephone or otherwise)
- ☐ Sending full medical report and images
- ☐ Access to reports via a shared region wide patient management system
- ☐ Discharge letter

12. Is there a form of coordination or structured collaboration between your hospital and one or more rehabilitation institutes and/or nursing homes in your region?

- No
- Yes

13. Which factors are considered in deciding on rehabilitation choice for a patient? Please rank the following factors from most to least important (1 to 5)

☐ Quality of care	1. …………………………….
☐ Distance to patients home	2. …………………………….
☐ Availability at short notice	3. …………………………….
☐ Specialized neuro-rehabilitation	4. …………………………….
☐ Funding / financial reason	5………………………………

The responses to this question should represent, as best as practicable, a general consensus in your center.

14. Please rank the satisfaction of your team on how these factors are met in your network (1 not satisfied at all – 5 completely satisfied).

☐ Quality of care	(1, 2, 3, 4, 5)
☐ Distance to patients home	(1, 2, 3, 4, 5)
☐ Availability at short notice	(1, 2, 3, 4, 5)
☐ Specialized neuro-rehabilitation	(1, 2, 3, 4, 5)

The responses to this question should represent, as best as practicable, a general consensus in your center.

Provider Profiling Questionnaire

Questionnaire 11: Country

This questionnaire can be completed by the CENTER-TBI local investigator with support from national organizations in the traumatic brain injury field

This questionnaire is about your country and not about your hospital only!
For the completion of this questionnaire, we advise you to ask support form national organizations in the traumatic brain injury field and/or other health organizations.

There are no 'right' or 'wrong' answers so please give us a realistic and honest view of how the care in your country is organized. Your answers will only be used to answer the scientific questions in CENTER TBI.

If you have any questions or problem, please contact:
Maryse Cnossen, PhD student (m.c.cnossen@erasmusmc.nl)

Information about the completer of the questionnaire

Other than the CENTER-TBI investigator, which of the following individuals was involved in completion of this questionnaire?

Select all that apply

- ☐ Neurologist
- ☐ Neurosurgeon
- ☐ Trauma Surgeon
- ☐ Emergency Department (ED) physician
- ☐ Administrative staff member / data manager / financial department
- ☐ National organization in the traumatic brain injury field
- ☐ Other, please specify……………..
- ☐ NA. The questionnaire was completed solely by the CENTER TBI local investigator

The Local investigator is the senior clinician(s) at your hospital involved in supervision of CENTER TBI

The following questions are about your country and not about your hospital only! You may find this information on websites of professional groups in your country and websites of insurance companies / government rapports.

Health Care Policy in your Country

1.Does your country have a policy to promote centralization of care for severe trauma patients?

- o No
- o Yes

2. Is intensive care a recognized specialism in your country?

- o No
- o Yes

In some countries, they do not use the word 'intensivist'. Instead, 'intensivists' may be referred to as "Anesthesia and Resuscitation" or another name. You can answer 'yes' here if there is a specialty in your country that educates towards intensive care doctor, regardless of how you call it. You can answer 'no' if there is no unique intensive care education program (doctors working at the ICU do for example have the same education as general anesthesiologists)

2b. If yes, is the training for ICU doctors additional to another specialism (e.g. anesthesia), or is it "stand alone"?

- ☐ Follow another specialty
- ☐ Stand alone

3. Is ER physician a recognized specialism in your country?

- ☐ No
- ☐ Yes

3b. If yes, is the training additional to another specialism (e.g. anesthesia), or is it "stand alone"?

- ☐ Follow another specialty
- ☐ Stand alone

4. What Glasgow Coma Scale (GCS) scores are considered as mild, moderate and severe Traumatic Brain Injury (TBI) in your country?

	Lowest GCS value	Highest GCS values
Mild TBI	……………………..	……………………..
Moderate TBI	……………………..	……………………..
Severe TBI	……………………..	……………………..

There are differences between countries in how they classify mild, moderate and severe TBI. Please give the lowest and highest GCS values that you consider as mild, moderate and severe. For example: severe TBI might have a lowest value of 3 and a highest value of 8.
You can use national guidelines here.

5. What specialty is most often responsible (in charge) for the treatment of Traumatic Brain Injury (TBI) patients in your country. Distinguish the following strata: Emergency Department (ED), admission to the hospital ward (the ward where TBI patients are generally admitted to in your country) and Intensive Care Unit (ICU)?

	ED	Hospital ward	ICU
ED physician	☐	☐	☐
Neurologist	☐	☐	☐
Neurosurgeon	☐	☐	☐
Intensivist	☐	☐	☐
Trauma Surgeon	☐	☐	☐
General / Orthopaedic Surgeon	☐	☐	☐
Other, please specify……………………….	☐	☐	☐
N/A. Care for TBI patients is always multidisciplinary in our country	☐	☐	☐

Please specify what specialism is most often <u>in charge in your country</u>. This does not automatically have to be the person with the most contact hours with the patients.

In this response, the term 'trauma surgeon' refers to an individual who specializes in trauma surgery, not a general surgeon or orthopaedic surgeon who happens to perform damage control surgery as part of wider responsibilities

Dispatch system in your country

6. Is there a central telephone number for all emergency services or are separate calls required for ambulance, fire brigade and police?

- ☐ Central telephone number. Please specify number: …………………………………….
- ☐ No central telephone number

7. Which system is used for dispatching ambulances?

- ☐ Automatic dispatching (ambulance stationary on post closest to the scene is mobilized)
- ☐ Dynamic ambulance management (ambulance which is vacant closest to the scene is mobilized)
- ☐ Selective dispatching (depends on the nature and urgency of the reported incidence).
- ☐ Others: ………………………….

8. What type of training is required for the dispatching personnel in your country?

- ☐ No special training
- ☐ Nursing
- ☐ Specialized training for medical dispatching
- ☐ Medical training
- ☐ Nursing + specialized training
- ☐ Other…………………

Insurance system in your country

9. Is a health care insurance compulsory for all inhabitants in your country (part of a social security system)?

- ☐ No
- ☐ Yes

9b. What is the percentage of people in your country who were uninsured in the year 2013?
….%

If there is no information available for the year 2013. You can use data from 2012 or 2011. If you do so, please note this

10. Do patients have to pay a part of the delivered care themselves (either acute care or postacute care)?

- ☐ No
- ☐ Yes

This can refer to a co-payment (patients have to pay a percentage of the care themselves, for example 3% of the hospital stay) or a deductible (patients have to pay for example the first 350 euro of all care costs a year themselves)

10b. If yes: do they pay a percentage of the care costs themselves (co-payment)?

- ☐ No
- ☐ Yes

A copayment relates to the situation in which patients have to pay a percentage of the care themselves, for example they have to pay 3% of the hospital stay.

10c. If yes (question about paying for delivered care): Do patients have to pay a deductible for care in your country?

- ☐ No
- ☐ Yes

10d. If yes: what is the standard deductible in your country?
……………………euro

10e. If yes: do inhabitants have the possibility to choose a higher deductible?

- ☐ No
- ☐ Yes. The maximum deductible is …………euro

A deductible relates to a situation in which patients have to pay the first for example 350 euro of all care costs a year themselves. In this situation it does not matter if the total care costs are 350 euro or 10 000 euro.<br>In some countries there is a minimum deductible that is applicable for all inhabitants.<br>People can however choose to pay a higher deductible (and a lower monthly premium).<br>If this is not applicable for your country (deductible does not vary) fill in your standard deductible twice.

11. When a hospital gets reimbursed from insurance companies, are all the cost items (e.g. CT scans, lab, interventions) charged separately or do you claim one all-in amount?

- ☐ All items separately

One total amount
