# Supplementary material for: Variation in Structure and Process of Care in Traumatic Brain Injury: Provider Profiles of European Neurotrauma Centers Participating in the CENTER-TBI Study
Source: PLoS One. 2016 Aug 29;11(8):e0161367. doi: 10.1371/journal.pone.0161367 (PMC5003388; doi:10.1371/journal.pone.0161367)
Supplement: S2 File — Note. Table presents all definitions used in the paper in the order that they are used in the results section of the paper. TBI = traumatic brain injury; ICU = intensive care unit (PDF) [file pone.0161367.s003.pdf]

Level I trauma center	A regional resource center that generally serves large cities or population-dense areas. A level I trauma center is expected to manage large numbers of severely injured patients (at least 1,200 trauma patients annually or have 240 admissions with an Injury Severity Score of more than 14). It is characterized by 24-hour in-house availability of an attending surgeon and the prompt availability of other specialties (e.g. neurosurgeon, trauma surgeon).
Level II trauma center	A level II trauma center provides comprehensive trauma care in either a population-dense area in which a level II trauma center may supplement the clinical activity and expertise of a level I institution or occur in less population-dense areas. In the latter case, the level II trauma center serves as the lead trauma facility for a geographic area when a level I institution is not geographically close enough to do so. It is characterized by 24-hour in-house availability of an attending surgeon and the prompt availability of other specialties (e.g. neurosurgeon, trauma surgeon).
Level III trauma center	A level III trauma center has the capacity to initially manage the majority of injured patients and have transfer agreements with a level I or II trauma center for seriously injured patients whose needs exceed the facility's resources.
Urban location	An hospital location very near to a city and situated in a crowded area.
Rural location	An hospital location in or very near to the countryside in an area that is not crowded. A suburban location was defined as in between urban and rural.
Acute trauma team	A multidisciplinary team that is alerted when a serious trauma victim is expected. The function of this team is early triage and treatment.
Dedicated neuro ICU	An ICU that is equipped to treat patients with neurological or neurosurgical injury
Closed ICU organization	An ICU organization in which critical care physicians (intensivists) assume primary responsibility for delivery of intensive care for TBI patients.
Open ICU organization	An ICU organization in which the admitting surgeon (neurosurgeon / trauma surgeon) assumed primary responsibility for care of TBI patients, including the

	provision of critical care services. This model of care may include elective consultation of an intensivist.
Mixed ICU organization	An ICU organization in which the admitting surgeon (neurosurgeon / trauma surgeon) assumes primary responsibility of care of TBI patients. A certified physician in critical care (intensivist) coordinates the delivery of care.
Step down beds	A facility in-between ICU and ward. It is often used for patients who improved at the intensive care and no longer need the intensity of ICU care, but are also not well enough to be cared for at the ward. The care provided in step down beds is less intensive than the care provided at the ICU but more intensive than ward care.
Damage control	A management strategy in patients with severe TBI and extremity fractures with a complete focus on the TBI rather than on the extremity fractures. Extremity fractures are stabilized but definitive treatment is delayed in this case.
Definitive care	A management strategy in patients with severe TBI and extremity fractures in which doctors try to operate or fixate the extremity fractures as soon as possible.
Co-payment	A system in which patients have to pay a percentage of the care themselves.
Deductible	A system in which patients have to pay a fixed amount of care costs a year if they use any care.
