# Supplementary material for: Variation in Structure and Process of Care in Traumatic Brain Injury: Provider Profiles of European Neurotrauma Centers Participating in the CENTER-TBI Study
Source: PLoS One. 2016 Aug 29;11(8):e0161367. doi: 10.1371/journal.pone.0161367 (PMC5003388; doi:10.1371/journal.pone.0161367)
Supplement: S1 Table — A P-value for the difference between high/middle and low income countries B P-value for the difference between North-West and South-East Europe and Israel (PDF) [file pone.0161367.s004.pdf]

Characteristic	Middle and high income countries (n = 58)	Low income countries (n =13)	P-value[A]	North and West Europe (n = 46)	East and South Europe and Israel (n = 25)	P-value[B]
The availability of a dedicated neuro ICU	35 (61%)	5 (39%)	.13	26 (58%)	14 (56%)	.89
Organization of the ICU			-			-
- Closed	40 (70%)	5 (39%)		27 (60%)	18 (72%)	
- Open	3 (5%)	0 (0%)		2 (4%)	1 (4%)	
- Mixed	14 (25%)	8 (61%)		16 (36%)	6 (24%)	
The availability of an in-hospital rehabilitation unit	28 (49%)	8 (62%)	.42	23 (51%)	13 (52%)	.94
Separate 24/7 emergency operation rooms	41 (71%)	12 (92%)	.16*	32 (70%)	21 (84%)	.18
Lab turnaround times			-			.34*
- 0-30 minutes	19 (35%)	6 (46%)		13 (30%)	12 (48%)	
- >30 minutes	21 (38%)	5 (39%)		18 (42%)	8 (32%)	
- Na. No alb SOP at the ED	15 (27%)	2 (15%)		12 (28%)	5 (20%)	
Step down beds	41 (72%)	9 (69%)	.99*	36 (80%)	14 (56%)	.03
In-hospital coma stimulation	27 (47%)	7 (54%)	.67	25 (56%)	9 (36%)	.12
Location TBI facilities			-			-
- Different buildings	15 (26%)	5 (38%)		14 (31%)	6 (24%)	
- Same building, different floors	38 (65%)	7 (54%)		30 (65%)	15 (60%)	
- Same building, same floor	5 (9%)	1 (8%)		2 (4%)	4 (16%)	
Night coverage ICU			-			-
- Certified intensivist / ICU physician	32 (58%)	12 (92%)		22 (51%)	22 (88%)	

-	Trainee	19 (35%)	1 (8%)	17 (40%)	3 (12%)
-	Fellow in training for ICU	4 (7%)	0 (0%)	4 (9%)	0 (0%)
