# Supplementary material for: Variation in Structure and Process of Care in Traumatic Brain Injury: Provider Profiles of European Neurotrauma Centers Participating in the CENTER-TBI Study
Source: PLoS One. 2016 Aug 29;11(8):e0161367. doi: 10.1371/journal.pone.0161367 (PMC5003388; doi:10.1371/journal.pone.0161367)
Supplement: S2 Table — A P-value for the difference between high/middle and low income countries B P-value for the difference between North-West and South-East Europe and Israel (PDF) [file pone.0161367.s005.pdf]

Characteristic	Middle and high income countries (n = 58)	Low income countries (n =13)	P-value[A]	North and West Europe (n = 46)	East and South Europe and Israel (n = 25)	P-value[B]
Patients with moderate TBI (GCS 9-12) without CT abnormalities are admitted to the ICU			.09*			.11
- No or only in the presence of other risk factors	38 (68%)	12 (92%)		29 (66%)	21 (82%)	
- General policy	18 (32%)	1 (8%)		15 (34%)	4 (16%)	
Patients with moderate TBI (GCS 9-12) with CT abnormalities are admitted to the ICU			.05*			.13
- No or only in the presence of other risk factors	17 (30%)	8 (62%)		13 (30%)	12 (48%)	
- General policy	39 (70%)	5 (38%)		31 (70%)	13 (52%)	
Patients with mild TBI (GCS 13-15) using anti-coagulant therapy			.27*			.29
- No or only in the presence of other risk factors	41 (73%)	12 (92%)		32 (73%)	21 (84%)	
- General policy	15 (27%)	1 (8%)		12 (27%)	4 (16%)	
ICP monitoring is performed in patients with GCS<9 without CT abnormalities			.43*			.05
- No or only in the presence of other risk factors	42 (75%)	10 (91%)		31 (70%)	21 (91%)	
- General policy	14 (25%)	1 (9%)		12 (30%)	2 (9%)	
ICP monitoring is performed in patients			.30*			.12

with intraventricular hemorrhages						
- No or only in the presence of other risk factors	40 (71%)	6 (55%)		33 (75%)	13 (56%)	
- General policy	16 (29%)	5 (45%)		11 (25%)	10 (44%)	
ICP sensors that are used at the ICU:			-			-
- Parenchymal	20 (36%)	1 (9%)		14 (32%)	7 (31%)	
- Ventricular	3 (5%)	3 (27%)		2 (4%)	4 (17%)	
- Both	33 (59%)	7 (64%)		28 (64%)	12 (52%)	
Threshold for decompressive craniotomy in elevated ICP			-			-
- >20mmHg	6 (12%)	1 (10%)		6 (16%)	1 (4%)	
- >25mmHg	28 (55%)	7 (70%)		23 (61%)	12 (52%)	
- >30mmHg	17 (33%)	2 (20%)		9 (24%)	10 (44%)	
Structural variation between (neuro)surgeons with regard to their decision to place an ICP sensor	25 (45%)	8 (62%)	.27	23 (52%)	10 (40%)	.33
